# Insulin enhances RAGE ectodomain shedding by inducing Rab14-dependent ADAM10 cell surface trafficking in human aortic endothelial cells

**DOI:** 10.1371/journal.pone.0358445

**Published:** 2026-09-18

**Authors:** Chung Hee Baek, Hyosang Kim, Soo Young Moon, Eun Kyoung Lee, Won Seok Yang

**Affiliations:** 1 Division of Nephrology, Department of Internal Medicine, Asan Medical Center, University of Ulsan College of Medicine, Seoul, Republic of Korea; 2 Asan Institute for Life Sciences, Seoul, Republic of Korea; 3 Division of Nephrology, Department of Internal Medicine, Dankook University Hospital, Dankook University College of Medicine, Cheonan, Republic of Korea; China Medical University, TAIWAN

## Abstract

Insulin attenuates the effects of advanced glycation end products (AGEs) by inducing a disintegrin and metalloprotease 10 (ADAM10)-mediated cleavage of the receptor for AGEs (RAGE); however, the molecular mechanism underlying this process remains incompletely understood. We investigated the mechanism by which insulin promotes ADAM10-mediated RAGE shedding in cultured human aortic endothelial cells (HAECs). AGE-modified bovine serum albumin (AGE-BSA) increased intercellular adhesion molecule-1 (ICAM-1) expression, whereas insulin pretreatment (0.1–100 nM) attenuated this effect. Mechanistically, insulin activated AKT1, AKT2, and AKT3, promoted ADAM10 translocation to the cell surface, and enhanced RAGE ectodomain shedding. In contrast, treatment with GI254023X (an ADAM10 inhibitor) or ADAM10 knockdown using siRNA abolished insulin-induced RAGE ectodomain shedding. Likewise, knockdown of AKT1, AKT2, or AKT3 using siRNA, as well as treatment with the pan-AKT inhibitor MK-2206, inhibited insulin-induced ADAM10 cell surface translocation and RAGE ectodomain shedding. Co-immunoprecipitation analysis further demonstrated an interaction between Rab14 and ADAM10. Insulin enhanced this interaction and promoted the translocation of both Rab14 and ADAM10 to the cell surface. Conversely, Rab14 knockdown blocked insulin-induced ADAM10 cell surface translocation and RAGE ectodomain shedding, thereby abolishing the protective effect of insulin against AGE-BSA-induced ICAM-1 expression. Collectively, these findings demonstrate that insulin promotes Rab14-mediated trafficking of ADAM10 to the cell surface through AKT activation in HAECs, resulting in enhanced RAGE ectodomain shedding.

## 1. Introduction

Diabetes mellitus is a major risk factor for atherosclerosis [1]. Advanced glycation end products (AGEs), which are generated under hyperglycemic conditions, contribute to the development of diabetic atherosclerosis by promoting inflammatory responses in the arterial wall [2]. The biological effects of AGEs are mediated primarily through the receptor for AGEs (RAGE), a cell surface receptor composed of an extracellular domain, a transmembrane region, and a short cytoplasmic tail [3].

Insulin lowers blood glucose levels by promoting glucose uptake in skeletal muscle cells and adipocytes [4]; however, it also exhibits anti-atherogenic properties. For example, insulin has been shown to reduce aortic atherosclerotic lesions in apolipoprotein E knockout mice [5] and to slow the progression of carotid intima-media thickness in patients with type 1 diabetes [6]. The anti-atherogenic effects of insulin may be partly attributed to its ability to counteract AGE-mediated responses. Insulin induces ectodomain shedding of RAGE through a disintegrin and metalloprotease 10 (ADAM10) [7], thereby reducing RAGE-mediated inflammatory responses to AGEs. However, the mechanism by which insulin enhances ADAM10 activity remains controversial. In macrophages, insulin has been reported to increase ADAM10 activity by upregulating ADAM10 mRNA and protein expression [7]. In contrast, insulin enhances ADAM10 activity in COS-7 cells without altering ADAM10 mRNA or protein levels [8].

ADAM10 is synthesized as pro-ADAM10 in the endoplasmic reticulum (ER). In the Golgi apparatus, furin or other proprotein convertases remove the prodomain of pro-ADAM10, generating the mature, active form of ADAM10 [9]. Under basal conditions, ADAM10 is predominantly localized in the Golgi apparatus, particularly the trans-Golgi network [10,11]. To function as a sheddase, ADAM10 must translocate to the cell surface [12]. In human aortic endothelial cells (HAECs), we previously demonstrated that extracellular calcium influx- or 5-aminoimidazole-4-carboxamide ribonucleoside (AICAR)-induced AMP-activated protein kinase (AMPK) activation, as well as SC79 (a cell-permeable AKT activator)-induced AKT (protein kinase B) activation, promotes ADAM10 translocation to the cell surface [13–15].

Rab14, a small GTPase, plays a critical role in ADAM10 cell surface translocation induced by AMPK and AKT activation [14,15]. TBC1D1 and TBC1D4 (AS160) function as Rab GTPase-activating proteins that stimulate GTP hydrolysis on Rab14, thereby maintaining Rab14 in its inactive GDP-bound state [16]. However, phosphorylation of TBC1D1 and TBC1D4 by activated AMPK or AKT diminishes their inhibitory effects on Rab14, allowing Rab14 activation [16].

AKT is a serine/threonine protein kinase comprising three isoforms, AKT1, AKT2, and AKT3, which are encoded by distinct genes [17]. In our previous study, SC79 activated all three AKT isoforms in HAECs, promoting ADAM10 cell surface translocation and enhancing its shedding activity. Insulin is also known to activate all three AKT isoforms [18]. Therefore, insulin may enhance ADAM10 shedding activity by promoting Rab14-dependent ADAM10 cell surface translocation through AKT activation. However, this hypothesis has not yet been experimentally tested.

This study aimed to elucidate how insulin promotes ADAM10 shedding activity in cultured HAECs. We first examined whether insulin induces ADAM10-mediated RAGE ectodomain shedding. We then investigated whether insulin promotes ADAM10 cell surface translocation and whether AKT is required for this process. Finally, we examined the role of Rab14 in insulin-induced ADAM10 cell surface translocation.

## 2. Materials and methods

### 2.1. HAEC culture and treatments

Primary HAECs, obtained from Lonza Walkersville (Walkersville, MD, USA), were cultured in endothelial growth medium-2 (Lonza Walkersville) for 3–5 passages. Cells from each culture dish were cryopreserved separately in individual cryovials. For experiments, cells were thawed, seeded onto tissue culture plates, and cultured for 24 h. The cells were then incubated for 16 h in Medium 199 supplemented with Hank’s salts (Thermo Fisher Scientific) and 2% fetal bovine serum to reduce variations in basal cellular conditions. Before treatment, the culture medium was replaced with serum-free Medium 199 supplemented with Hank’s salts (Ca² ⁺ 1.26 mM) to minimize the effects of undefined serum components and potential background interference.

The following reagents were used for cell treatments: insulin (MedChemExpress, HY-P0035), AGE-modified bovine serum albumin (AGE-BSA; Cayman Chemical), MK-2206 (MedChemExpress, HY-10358), GI254023X (Sigma-Aldrich), and dimethyl sulfoxide (DMSO; Sigma-Aldrich). Insulin was dissolved in phosphate-buffered saline (PBS), whereas MK-2206 and GI254023X were dissolved in DMSO. AGE-BSA was used at a concentration of 100 µg/mL, as this concentration did not significantly affect HAEC viability and effectively induced ICAM-1 expression, as demonstrated in our previous study [13]. To inhibit AKT and ADAM10 activity, HAECs were treated with 1 µM MK-2206 and 2 µM GI254023X, respectively. These concentrations were selected based on previously reported conditions [19,20], and neither treatment altered cell morphology or total protein abundance under the experimental conditions.

### 2.2. Small interfering RNA (siRNA) transfection

The following siRNAs were used: ADAM10-siRNA (Ambion®), Rab14-siRNA (Ambion®), control siRNA (Ambion®), AKT1-siRNA (Santa Cruz Biotechnology, sc-29195), AKT2-siRNA (Santa Cruz Biotechnology, sc-29197), AKT3-siRNA (Santa Cruz Biotechnology, sc-38911), and control siRNA (Santa Cruz Biotechnology, sc-37007). HAECs were seeded and cultured in EBM-2 endothelial growth basal medium for 24 h. siRNA–Lipofectamine complexes were prepared by mixing siRNAs with Lipofectamine (Life Technologies/Thermo Fisher Scientific) diluted in Opti-MEM medium (Life Technologies/Thermo Fisher Scientific) and incubating the mixture at room temperature for 15 min. The culture medium was then replaced with serum-free Medium 199 supplemented with Hank’s salts, and cells were transfected with the siRNA–Lipofectamine complexes for 6 h. After transfection, cells were maintained in EBM-2 endothelial growth basal medium for an additional 18 h before subsequent experiments.

### 2.3. Western blot analysis

Western blotting was performed using whole-cell lysates and conditioned media as indicated. Whole-cell lysates were prepared as follows: cells were lysed with cold RIPA buffer (50 mM Tris-HCl, pH 7.4, 150 mM NaCl, 0.25% sodium deoxycholate, 1% NP-40, and protease and phosphatase inhibitors) and incubated on ice for 10 min. Lysates were collected, transferred to microcentrifuge tubes, and centrifuged at 10,000 × g for 5 min at 4°C. The resulting supernatants were collected as whole-cell lysates. Protein concentrations were determined using the Bradford method.

Equal amounts of protein from whole-cell lysates were separated by sodium dodecyl sulfate–polyacrylamide gel electrophoresis (SDS-PAGE) and transferred to Immobilon-P membranes (EMD Millipore, Bedford, MA, USA). Membranes were incubated with primary antibodies, followed by horseradish peroxidase (HRP)-conjugated secondary antibodies. The primary antibodies used were as follows: anti-RAGE (Santa Cruz Biotechnology, sc-80652; mouse monoclonal antibody recognizing a truncated extracellular domain of RAGE), anti-RAGE (JF0975; NBP2–67095, R&D Systems; rabbit monoclonal antibody recognizing amino acids 350–390 corresponding to the C-terminal region of human RAGE), anti-ADAM10 (Santa Cruz Biotechnology, sc-28358), anti-Rab14 (Santa Cruz Biotechnology, sc-271401), anti-ICAM-1 (Santa Cruz Biotechnology, sc-7891), anti-actin (Santa Cruz Biotechnology, sc-47778), anti-AKT1 (ABclonal, A11016), anti-AKT2 (Cell Signaling Technology, #3063), anti-AKT3 (ABclonal, A12909), anti-p-AKT1 (Ser473) (ABclonal, AP0140), anti-p-AKT2 (Ser474) (ABclonal, AP0005), and anti-p-AKT3 (Ser472) (Abnova Corporation, PAB8141). After washing, membranes were incubated with Luminata Forte Western HRP Substrate (EMD Millipore), and chemiluminescent signals were detected by exposure to X-ray film. Films were scanned, and band intensities were quantified using ImageJ software (National Institutes of Health, Bethesda, MD, USA).

For Western blot analysis of conditioned media, equal volumes of samples were concentrated using Amicon® Ultra centrifugal filters (Ultracel®-10K, EMD Millipore) before loading onto SDS-PAGE gels. Following electrophoretic separation, proteins were transferred to Immobilon-P membranes and incubated with an antibody against the truncated extracellular domain of RAGE (Santa Cruz Biotechnology, sc-80652).

### 2.4. Cell surface ADAM10 immunofluorescence staining

Cells were cultured in 60-mm dishes containing coverslips placed on the bottom of each dish. Following experimental treatments, the coverslips were removed, and cells were fixed with 4% paraformaldehyde for 10 min. To detect cell surface ADAM10, immunofluorescence staining was performed without membrane permeabilization. Cells were blocked with 1% bovine serum albumin in PBS for 60 min to prevent nonspecific antibody binding. The cells were then incubated overnight at 4°C with an antibody against the extracellular domain of ADAM10 (ABclonal, A10438; rabbit antibody recognizing amino acids 214–500 of ADAM10). After washing with PBS, cells were incubated with an Alexa Fluor 488-conjugated secondary antibody. Nuclei were subsequently stained with 4′,6-diamidino-2-phenylindole (DAPI; Sigma-Aldrich). Immunofluorescence images were acquired using a Zeiss LSM710 laser-scanning confocal microscope (Carl Zeiss, Oberkochen, Germany).

Fluorescence intensities were quantified in cells completely contained within the field of view, excluding cells partially captured at the image borders. The fluorescence intensity of each cell was measured using ImageJ software (National Institutes of Health) and calculated using the following formula: Corrected cell fluorescence = integrated density − (cell area × mean background fluorescence).

Cells remaining in the culture dishes outside the coverslips were collected and used to determine the levels of ADAM10, AKT1, AKT2, AKT3, or Rab14 by Western blot analysis.

### 2.5. Co-immunoprecipitation

Co-immunoprecipitation was performed to analyze the interaction between Rab14 and ADAM10. Briefly, cells were seeded in 100-mm culture dishes and incubated with or without insulin for 20 min. Cells were then lysed in IP lysis buffer (50 mM Tris-HCl, pH 7.4, 150 mM NaCl, 1% NP-40, and protease inhibitors), and whole-cell lysates were prepared. For preclearing, 500 µg of lysate was incubated with 20 µL of protein A agarose (Santa Cruz Biotechnology, sc-2001) and 1 µg of rabbit control IgG (Cell Signaling Technology, #2729) for 30 min. After centrifugation, the supernatants were collected and incubated overnight with 4 µg of rabbit control IgG or rabbit anti-ADAM10 monoclonal antibody (JM32–11; Thermo Fisher Scientific, MA5–32616), followed by incubation with 20 µL of protein A agarose beads for 4 h. The protein A agarose beads were collected, washed, and incubated with 5 × SDS loading buffer to elute the immunoprecipitated proteins. Western blot analysis was performed on whole-cell lysates (input) and immunoprecipitated samples using mouse monoclonal antibodies against ADAM10 (Santa Cruz Biotechnology, sc-28358) and Rab14 (Santa Cruz Biotechnology, sc-271401).

### 2.6. Isolation of cell surface and intracellular proteins

The Pierce™ Cell Surface Protein Biotinylation and Isolation Kit (Thermo Fisher Scientific) was used to evaluate the cell surface localization of Rab14 and ADAM10 following insulin treatment. Briefly, cells were seeded and cultured in 100-mm culture dishes. After incubation with or without insulin for 20 min, cells were treated with the membrane-impermeable sulfo-NHS-SS-biotin reagent for 10 min at room temperature to biotinylate cell surface proteins. Whole-cell lysates were then prepared and incubated with NeutrAvidin agarose beads to capture biotinylated proteins. After centrifugation, the supernatants were collected as the intracellular protein fraction. Equal amounts of intracellular proteins were concentrated using Amicon® Ultra centrifugal filters (Ultracel®-10K, EMD Millipore). The NeutrAvidin agarose bead-bound biotinylated surface proteins were washed and incubated with dithiothreitol-containing elution buffer to release cell surface proteins. The levels of Rab14 and ADAM10 in whole-cell lysates, intracellular fractions, and cell surface fractions were determined by Western blot analysis.

### 2.7. Statistical analysis

Data are presented as mean ± SEM. The Shapiro–Wilk test was used to assess data normality, and no significant deviations from a normal distribution were observed. Comparisons between two independent groups were performed using Student’s *t*-test. Differences among three or more groups were analyzed using one-way analysis of variance (ANOVA), followed by Tukey’s multiple comparisons test. Data involving two independent variables (siRNA knockdown and cell treatment) were analyzed using two-way ANOVA, followed by Šidák-adjusted multiple comparisons. Statistical analyses were performed using GraphPad Prism version 11.0.2 for Windows (GraphPad Software, Boston, MA, USA). A p value < 0.05 was considered statistically significant.

## 3. Results

### 3.1. Insulin pretreatment inhibits AGE-BSA-induced ICAM-1 expression

**Fig 1:**
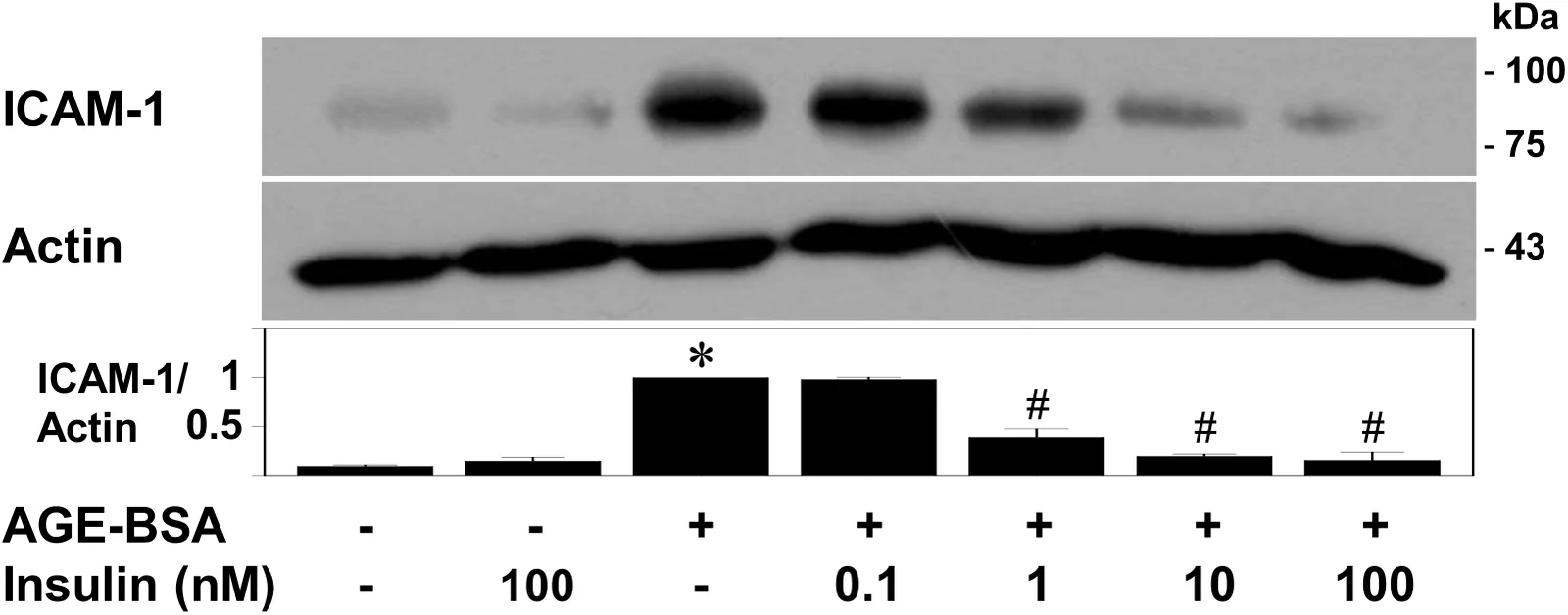
Insulin pretreatment inhibits AGE-BSA-induced ICAM-1 expression. HAECs were pretreated with the indicated concentrations of insulin for 30 min, followed by incubation with AGE-BSA (100 μg/mL) for 24 h. Cell lysates were analyzed by Western blot using antibodies against ICAM-1 and actin. (n = 3 independent experiments, ^*^p < 0.05 vs. control; ^#^p < 0.05 vs. AGE-BSA).

In HAECs, AGE-BSA increased ICAM-1 expression; however, insulin pretreatment attenuated AGE-BSA-induced ICAM-1 expression in a concentration-dependent manner at concentrations ranging from 0.1 to 100 nM (Fig 1). Our previous study demonstrated that AGE-BSA induces ICAM-1 expression through RAGE-mediated signaling, as RAGE knockdown by siRNA attenuated this response [13].

### 3.2. Insulin promotes RAGE ectodomain shedding

Ectodomain cleavage of RAGE by metalloproteases occurs near the cell surface, generating two fragments: an N-terminal ectodomain fragment and a C-terminal membrane-associated fragment. The N-terminal fragment is released into the culture medium, whereas the C-terminal fragment remains within the cells. HAECs were treated with 100 nM insulin for 5, 10, 30, or 60 min or with the indicated concentrations of insulin (0.1, 1, 10, or 100 nM) for 30 min. Cell lysates and conditioned media were analyzed by Western blotting. The shed N-terminal fragment was detected using a monoclonal antibody against the RAGE extracellular domain, whereas the C-terminal fragment was detected using a monoclonal antibody recognizing human RAGE amino acids 350–390.

**Fig 2:**
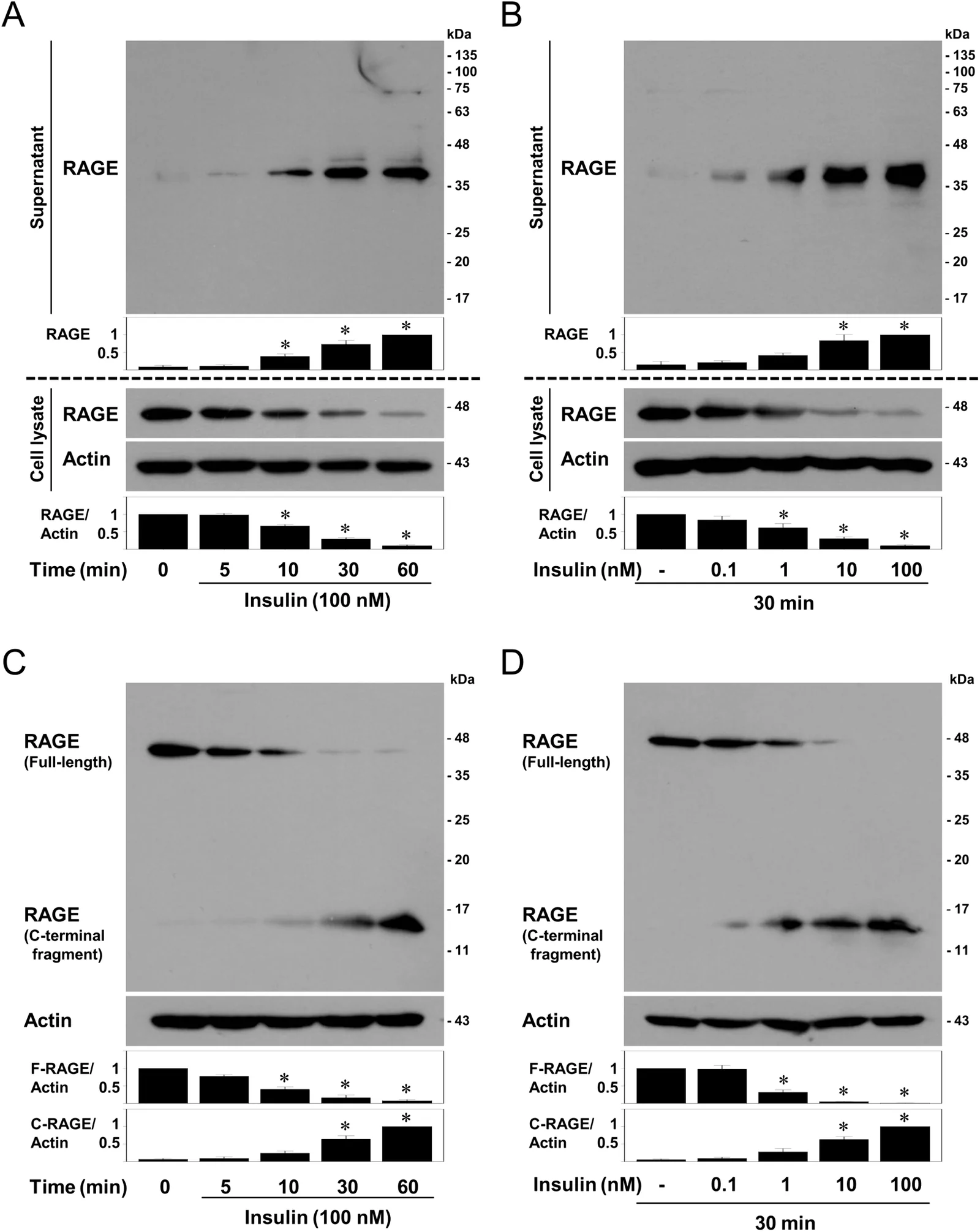
Insulin promotes RAGE ectodomain shedding. (A, B) HAECs were treated with 100 nM insulin for 5, 10, 30, or 60 min (n = 3 independent experiments) or with the indicated concentrations of insulin (0.1, 1, 10, or 100 nM) for 30 min (n = 3 independent experiments). Cell lysates and culture supernatants were analyzed by Western blot using antibodies against the RAGE extracellular domain and actin.(C, D) HAECs were treated with 100 nM insulin for 5, 10, 30, or 60 min (n = 4 independent experiments) or with the indicated concentrations of insulin (0.1, 1, 10, or 100 nM) for 30 min (n = 4 independent experiments). Cell lysates were analyzed by Western blot using antibodies against the RAGE C-terminal domain and actin. (^*^p < 0.05 vs. control).

Insulin treatment reduced full-length RAGE levels in cell lysates while increasing the levels of the shed RAGE ectodomain fragment in conditioned media in a time- and concentration-dependent manner (Fig 2A and 2B). In addition, insulin induced a time- and concentration-dependent increase in the intracellular C-terminal fragment of RAGE, accompanied by a reduction in full-length RAGE (Fig 2C and 2D). Together, these findings demonstrate that insulin induces RAGE ectodomain shedding.

### 3.3. AKT or ADAM10 inhibition abolishes insulin-induced RAGE ectodomain shedding

**Fig 3:**
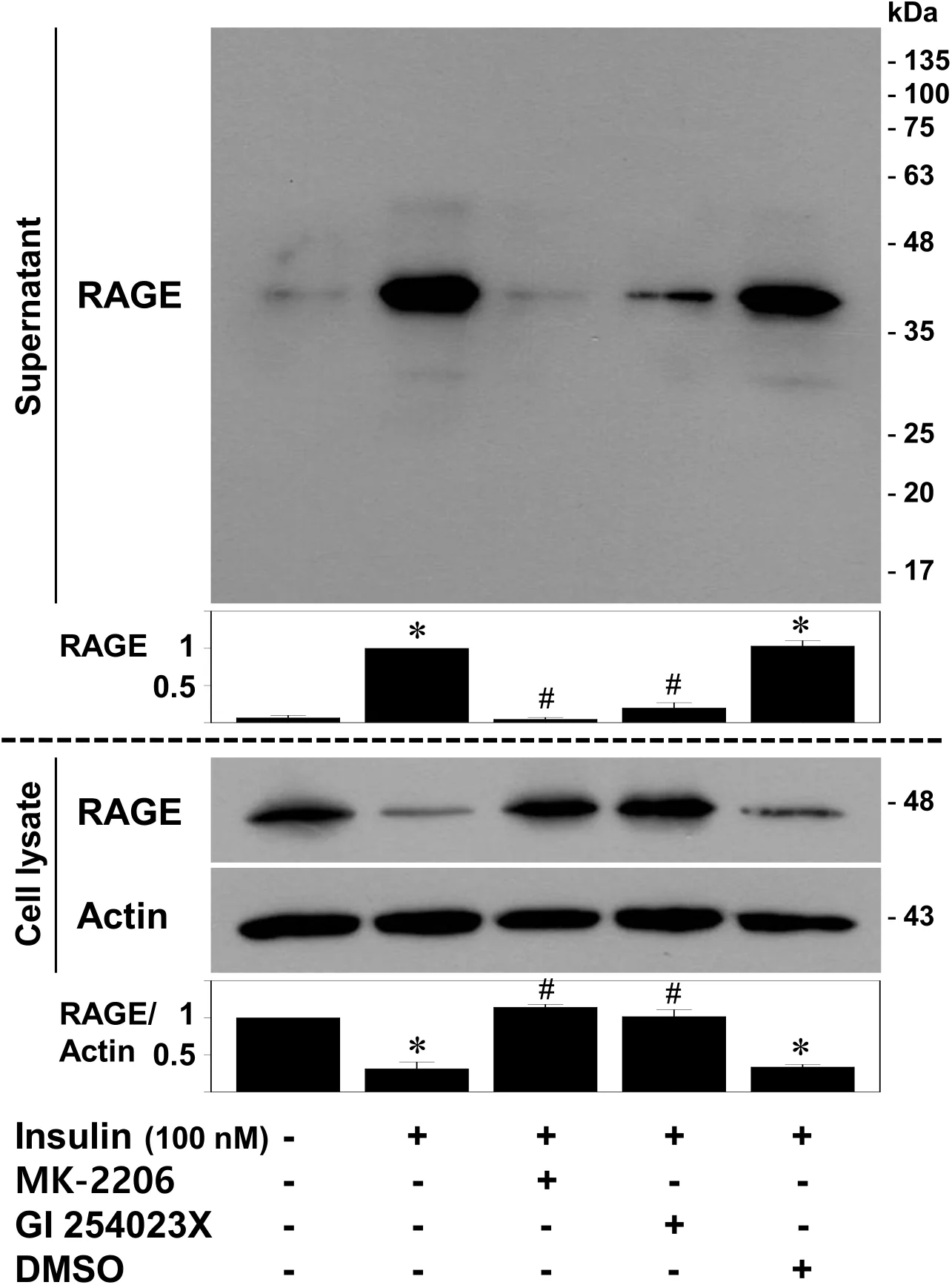
AKT or ADAM10 inhibition abolishes insulin-induced RAGE ectodomain shedding. HAECs were pretreated with MK-2206 (1 μM), GI254023X (2 μM), or DMSO (vehicle) for 60 min, followed by treatment with or without insulin (100 nM) for 30 min. Cell lysates and culture supernatants were analyzed by Western blot using antibodies against the RAGE extracellular domain and actin. (n = 3 independent experiments, ^*^p < 0.05 vs. control, ^#^p < 0.05 vs. insulin treatment alone).

In the following experiments, 100 nM insulin was used to investigate the mechanism underlying insulin-induced RAGE ectodomain shedding. To examine the involvement of AKT and ADAM10, we treated HAECs with their respective pharmacological inhibitors. MK-2206 is a pan-AKT inhibitor, whereas GI254023X is an ADAM10 inhibitor. Both inhibitors attenuated insulin-induced RAGE ectodomain shedding (Fig 3).

### 3.4. Involvement of AKT isoforms in insulin-induced RAGE ectodomain shedding

**Fig 4:**
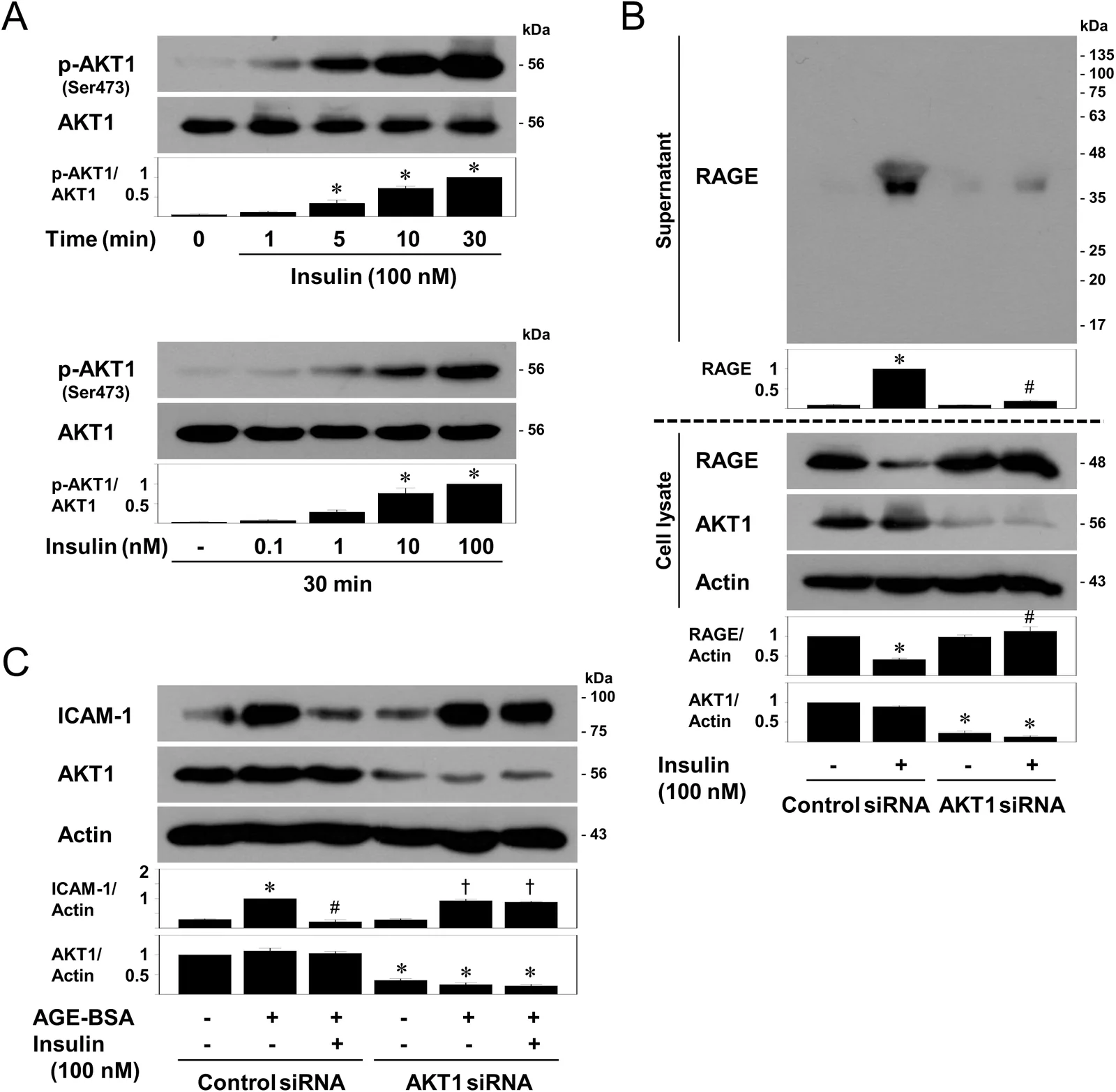
Involvement of AKT1 in insulin-induced RAGE ectodomain shedding. (A) Insulin activates AKT1. HAECs were treated with 100 nM insulin for 1, 5, 10, or 30 min (upper panel) or with 0.1, 1, 10, or 100 nM insulin for 30 min (lower panel). Cell lysates were analyzed by Western blot using antibodies against phospho-AKT1 (Ser473) and total AKT1. (n = 3 independent experiments, ^*^p < 0.05 vs. control). (B) AKT1 depletion abolishes insulin-induced RAGE ectodomain shedding. HAECs transfected with control siRNA or AKT1 siRNA were treated with or without 100 nM insulin for 30 min. Cell lysates and culture supernatants were analyzed by Western blot using antibodies against the RAGE extracellular domain, AKT1, and actin. (n = 3 independent experiments, ^*^p < 0.05 vs. control cells transfected with control siRNA; ^#^p < 0.05 vs. insulin-treated cells following control siRNA transfection). (C) AKT1 depletion abolishes the inhibitory effect of insulin on AGE-BSA-induced ICAM-1 expression. HAECs transfected with control siRNA or AKT1 siRNA were pretreated with or without 100 nM insulin for 30 min, followed by incubation with AGE-BSA (100 μg/mL) for 24 h. Cell lysates were analyzed by Western blot using antibodies against ICAM-1, AKT1, and actin. (n = 4 independent experiments, ^*^p < 0.05 vs. control; ^#^p < 0.05 vs. AGE-BSA; ^†^p < 0.05 vs. control cells transfected with AKT1-siRNA).

**Fig 5:**
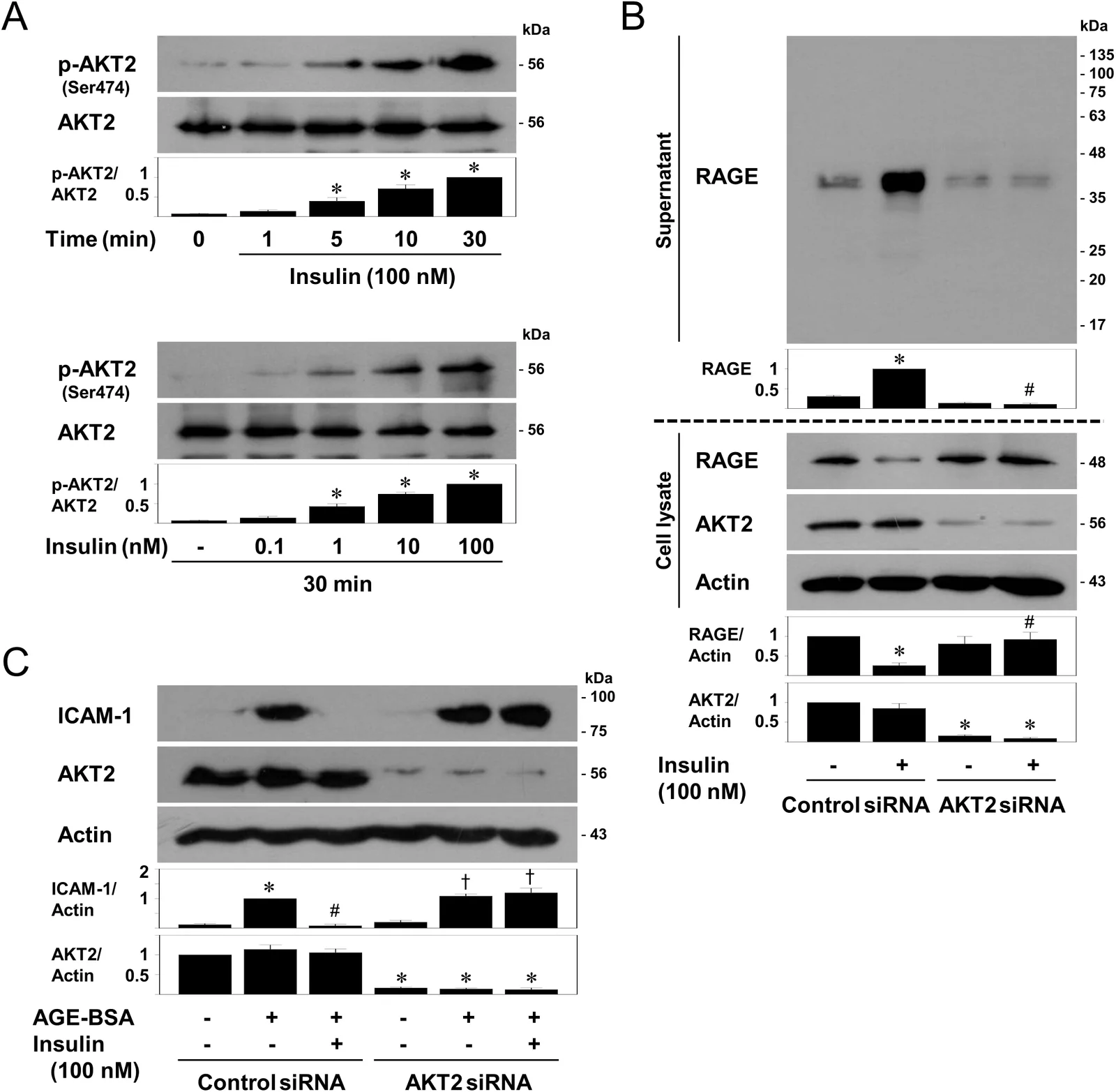
Involvement of AKT2 in insulin-induced RAGE ectodomain shedding. (A) Insulin activates AKT2. HAECs were treated with 100 nM insulin for 1, 5, 10, or 30 min (upper panel) or with 0.1, 1, 10, or 100 nM insulin for 30 min (lower panel). Cell lysates were analyzed by Western blot using antibodies against phospho-AKT2 (Ser474) and total AKT2. (n = 3 independent experiments, ^*^p < 0.05 vs. control). (B) AKT2 depletion abolishes insulin-induced RAGE ectodomain shedding. HAECs transfected with control siRNA or AKT2 siRNA were treated with or without 100 nM insulin for 30 min. Cell lysates and culture supernatants were analyzed by Western blot using antibodies against the RAGE extracellular domain, AKT2, and actin. (n = 3 independent experiments, ^*^p < 0.05 vs. control cells transfected with control siRNA; ^#^p < 0.05 vs. insulin-treated cells following control siRNA transfection). (C) AKT2 depletion abolishes the inhibitory effect of insulin on AGE-BSA-induced ICAM-1 expression. HAECs transfected with control siRNA or AKT2 siRNA were pretreated with or without 100 nM insulin for 30 min, followed by incubation with AGE-BSA (100 μg/mL) for 24 h. Cell lysates were analyzed by Western blot using antibodies against ICAM-1, AKT2, and actin. (n = 4 independent experiments, ^*^p < 0.05 vs. control; ^#^p < 0.05 vs. AGE-BSA; ^†^p < 0.05 vs. control cells transfected with AKT2-siRNA).

**Fig 6:**
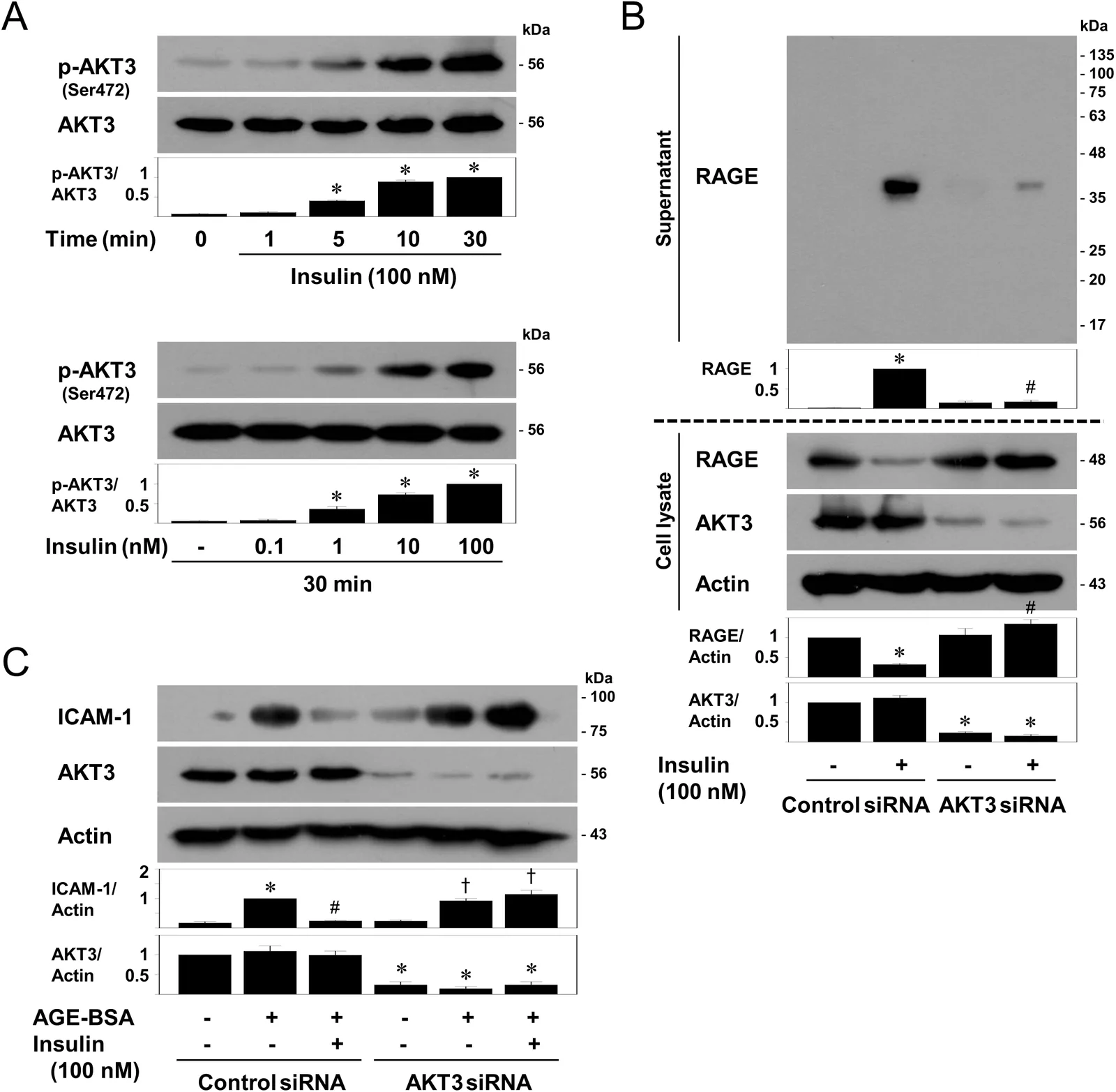
Involvement of AKT3 in insulin-induced RAGE ectodomain shedding. (A) Insulin activates AKT3. HAECs were treated with 100 nM insulin for 1, 5, 10, or 30 min (upper panel) or with 0.1, 1, 10, or 100 nM insulin for 30 min (lower panel). Cell lysates were analyzed by Western blot using antibodies against phospho-AKT3 (Ser472) and total AKT3. (n = 3 independent experiments, ^*^p < 0.05 vs. control). (B) AKT3 depletion abolishes insulin-induced RAGE ectodomain shedding. HAECs transfected with control siRNA or AKT3 siRNA were treated with or without 100 nM insulin for 30 min. Cell lysates and culture supernatants were analyzed by Western blot using antibodies against the RAGE extracellular domain, AKT3, and actin. (n = 3 independent experiments, ^*^p < 0.05 vs. control cells transfected with control siRNA; ^#^p < 0.05 vs. insulin-treated cells following control siRNA transfection). (C) AKT3 depletion abolishes the inhibitory effect of insulin on AGE-BSA-induced ICAM-1 expression. HAECs transfected with control siRNA or AKT3 siRNA were pretreated with or without 100 nM insulin for 30 min, followed by incubation with AGE-BSA (100 μg/mL) for 24 h. Cell lysates were analyzed by Western blot using antibodies against ICAM-1, AKT3, and actin. (n = 4 independent experiments, ^*^p < 0.05 vs. control; ^#^p < 0.05 vs. AGE-BSA; ^†^p < 0.05 vs. control cells transfected with AKT3-siRNA).

Because MK-2206 inhibited insulin-induced RAGE ectodomain shedding, we next investigated which AKT isoforms are involved in this process. Insulin increased the phosphorylation of all three AKT isoforms, AKT1, AKT2, and AKT3, indicating their activation (Figs 4A, 5A, and 6A). Conversely, siRNA-mediated knockdown of AKT1, AKT2, or AKT3 attenuated insulin-induced RAGE ectodomain shedding (Figs 4B, 5B, and 6B). In addition, knockdown of AKT1, AKT2, or AKT3 abolished the inhibitory effect of insulin on AGE-BSA-induced ICAM-1 expression (Figs 4C, 5C, and 6C).

### 3.5. Insulin promotes ADAM10 cell surface translocation, leading to RAGE ectodomain shedding

Under basal conditions, the majority of ADAM10 is localized in intracellular compartments, including the trans-Golgi network. To interact with its substrates, ADAM10 must translocate to the cell surface. To determine whether insulin promotes ADAM10 cell surface translocation, we examined cell surface ADAM10 levels before and after insulin treatment. HAECs were cultured in dishes containing coverslips. Cell surface ADAM10 was evaluated by immunofluorescence staining of cells grown on the coverslips using an antibody against the extracellular domain of ADAM10. Total cellular ADAM10 levels were measured by Western blot analysis using cells cultured outside the coverslips.

**Fig 7:**
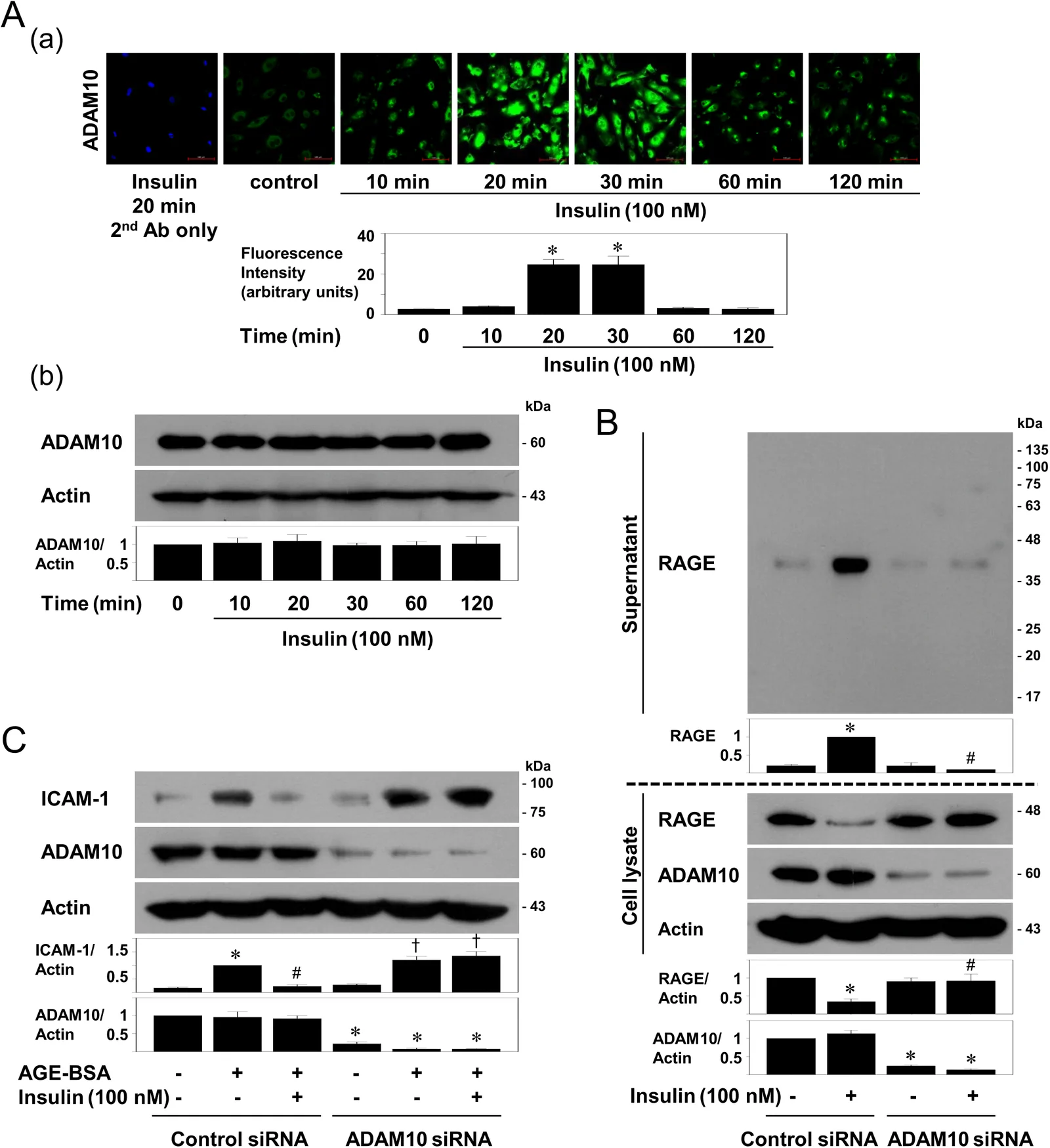
Insulin promotes ADAM10 cell surface translocation, leading to RAGE ectodomain shedding. (A) Insulin promotes ADAM10 cell surface translocation. HAECs were cultured on glass coverslips placed in cell culture dishes and treated with 100 nM insulin for up to 120 min. (a) Cells grown on coverslips were subjected to immunofluorescence staining to assess cell surface ADAM10. Representative images and the relative fluorescence intensities are shown (scale bar, 100 μm). (b) Cells cultured in the same dishes but outside the coverslips were analyzed by Western blot to determine total ADAM10 expression. (n = 3 independent experiments, ^*^p < 0.05 vs. control). (B) ADAM10 depletion abolishes insulin-induced RAGE ectodomain shedding. HAECs transfected with control siRNA or ADAM10 siRNA were treated with or without 100 nM insulin for 30 min. Cell lysates and culture supernatants were analyzed by Western blot using antibodies against the RAGE extracellular domain, ADAM10, and actin. (n = 3 independent experiments, ^*^p < 0.05 vs. control cells transfected with control-siRNA; ^#^p < 0.05 vs. insulin-treated cells following control siRNA transfection). (C) ADAM10 depletion abolishes the inhibitory effect of insulin on AGE-BSA-induced ICAM-1 expression. HAECs transfected with control siRNA or ADAM10 siRNA were pretreated with or without 100 nM insulin for 30 min, followed by incubation with AGE-BSA (100 μg/mL) for 24 h. Cell lysates were analyzed by Western blot using antibodies against ICAM-1, ADAM10, and actin. (n = 4 independent experiments, ^*^p < 0.05 vs. control; ^#^p < 0.05 vs. AGE-BSA; ^†^p < 0.05 vs. control cells transfected with ADAM10-siRNA).

As shown in Fig 7A, cell surface ADAM10 staining was weak in control cells. In contrast, insulin markedly increased cell surface ADAM10 levels after 20–30 min, followed by a rapid decline thereafter. Although the amount of accumulated shed RAGE fragments in the culture medium continued to increase over time (Fig 2), cell surface ADAM10 levels returned to baseline by 60 min (Fig 7A). Insulin treatment for up to 120 min did not alter total cellular ADAM10 levels (Fig 7A). These findings suggest that insulin promotes the translocation of intracellular ADAM10 to the cell surface rather than increasing ADAM10 protein levels.

To determine whether ADAM10 mediates insulin-induced RAGE ectodomain shedding, HAECs were transfected with ADAM10 siRNA. As shown in Fig 7B, ADAM10 knockdown attenuated insulin-induced RAGE ectodomain shedding, demonstrating that ADAM10 is required for this process.

We next examined whether ADAM10 is required for the protective effect of insulin against AGE-BSA-induced ICAM-1 expression. As shown in Fig 7C, ADAM10 knockdown abolished the inhibitory effect of insulin on AGE-BSA-induced ICAM-1 expression.

### 3.6. Involvement of AKT isoforms in insulin-induced ADAM10 cell surface translocation

**Fig 8:**
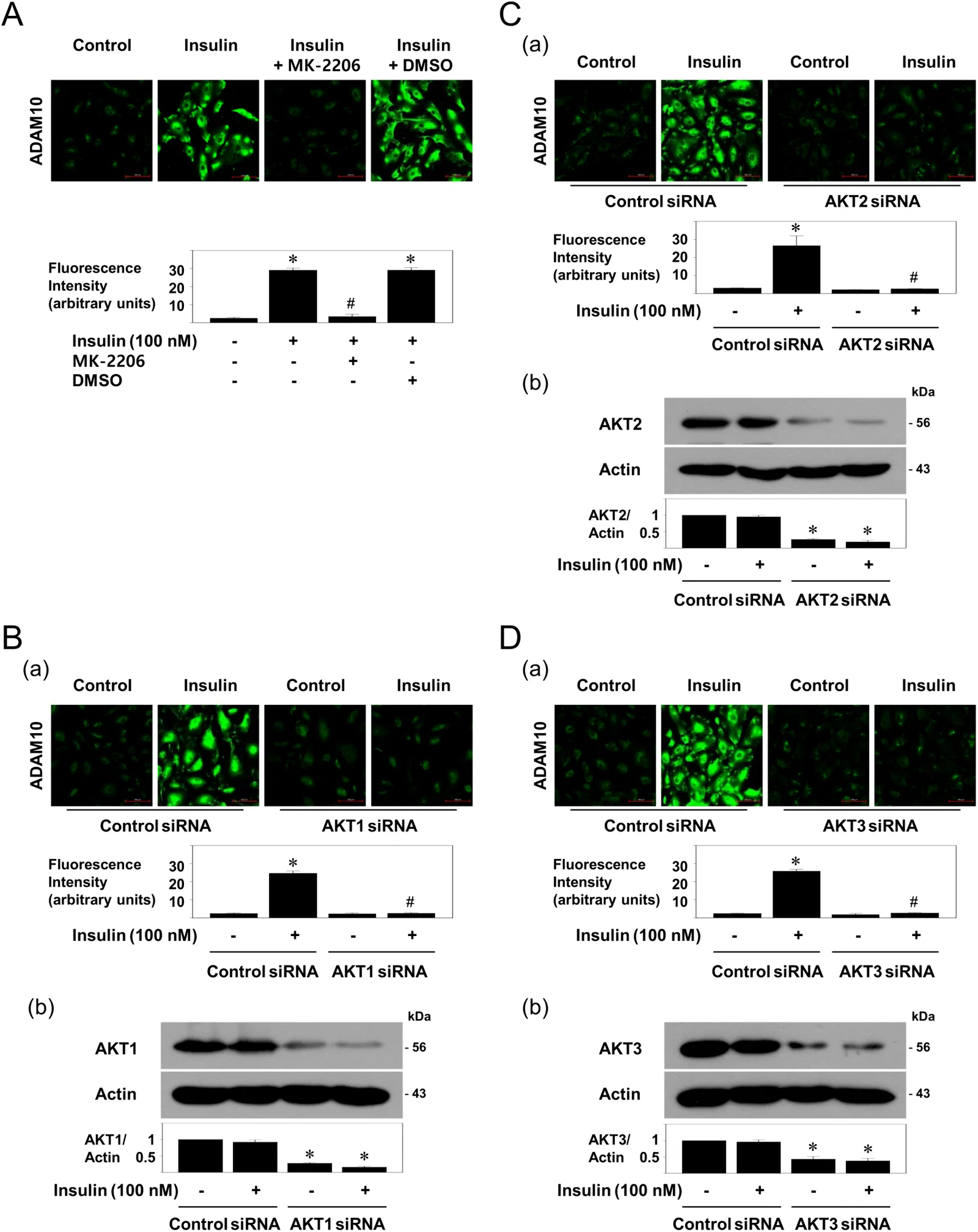
Involvement of AKT isoforms in insulin-induced ADAM10 cell surface translocation. HAECs were cultured on glass coverslips placed in cell culture dishes. (A) Cells were pretreated with MK-2206 (1 μM) or DMSO (vehicle) for 60 min, followed by treatment with or without insulin (100 nM) for 20 min. Cell surface ADAM10 was assessed by immunofluorescence staining. Representative images and the relative fluorescence intensities are shown (scale bar, 100 μm). (n = 3 independent experiments, ^*^p < 0.05 vs. control; ^#^p < 0.05 vs. insulin). (B-D) HAECs were transfected with control siRNA, AKT1 siRNA, AKT2 siRNA, or AKT3 siRNA and then treated with or without insulin (100 nM) for 20 min. (a) Cells grown on coverslips were subjected to immunofluorescence staining to assess cell surface ADAM10. Representative images and the relative fluorescence intensities are shown (scale bar, 100 μm).(b) Cells cultured in the same dishes but outside the coverslips were analyzed by Western blot to confirm knockdown of AKT1, AKT2, or AKT3. (n = 3 independent experiments, ^*^p < 0.05 vs. control cells transfected with control siRNA; ^#^p < 0.05 vs. insulin-treated cells following control siRNA transfection).

Because siRNA-mediated depletion of AKT1, AKT2, or AKT3 inhibited insulin-induced ADAM10-mediated RAGE ectodomain shedding, we investigated whether these AKT isoforms regulate ADAM10 cell surface translocation. As shown in Fig 8, siRNA-mediated knockdown of AKT1, AKT2, or AKT3, as well as pharmacological inhibition of AKT with MK-2206, attenuated the insulin-induced increase in cell surface ADAM10 levels.

### 3.7. Insulin enhances Rab14–ADAM10 interaction and promotes their translocation to the cell surface

In our previous study [15], Rab14 was shown to interact with ADAM10 and to be required for AKT activation-induced ADAM10 cell surface trafficking. To determine the involvement of Rab14 in insulin-induced ADAM10 cell surface translocation, we examined whether insulin regulates the interaction between Rab14 and ADAM10 and promotes Rab14 translocation to the cell surface.

**Fig 9:**
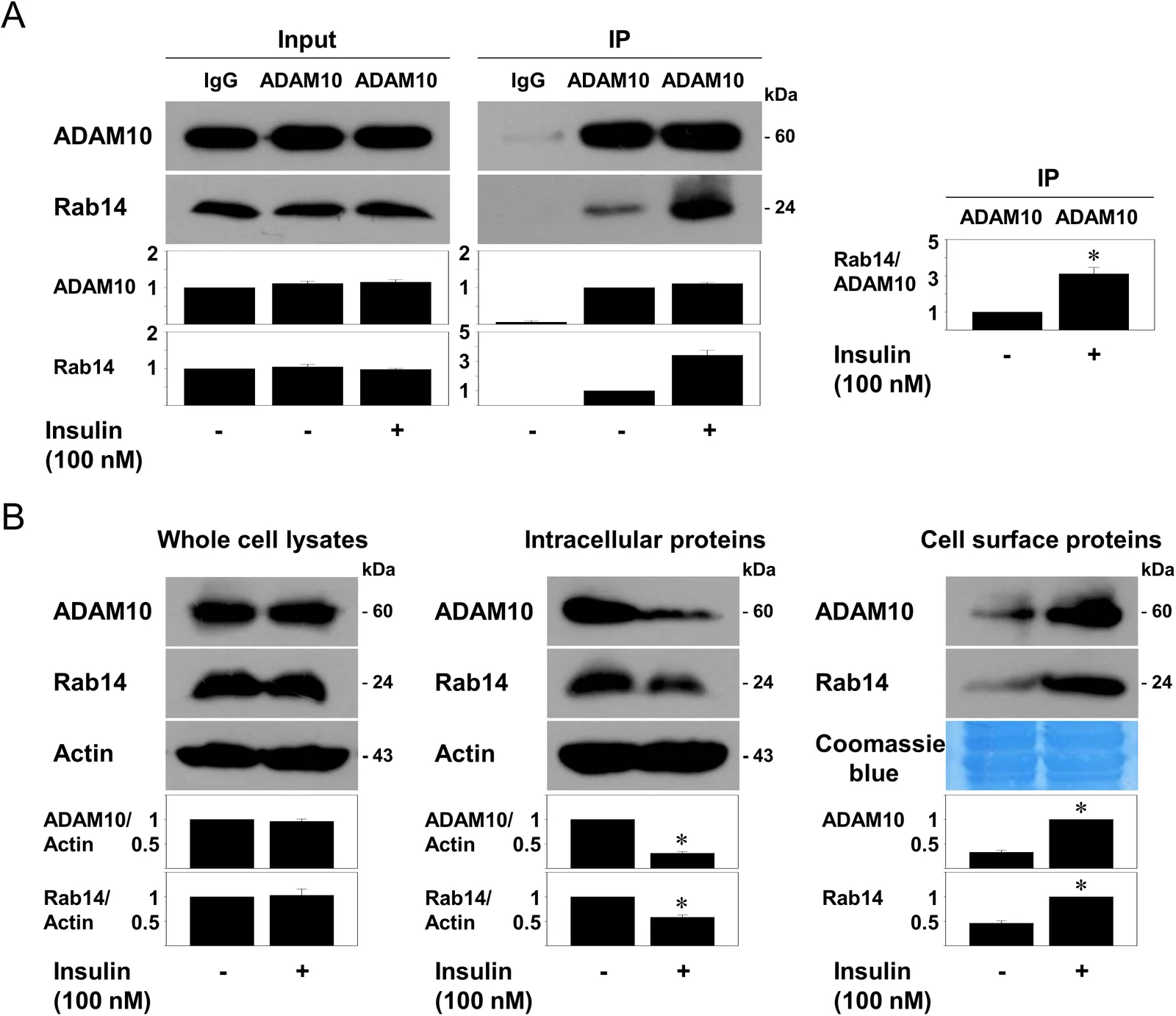
Insulin enhances Rab14–ADAM10 interaction and promotes their translocation to the cell surface. (A) Insulin enhances the interaction between Rab14 and ADAM10. HAECs were treated with or without insulin (100 nM) for 20 min. Cell lysates were incubated overnight at 4°C with control IgG or an anti-ADAM10 antibody, followed by incubation with protein A-agarose. Input lysates and immunoprecipitates were analyzed by Western blot using antibodies against ADAM10 and Rab14. (n = 6 independent experiments, ^*^p < 0.05 vs. control). (B) Insulin promotes the translocation of Rab14 and ADAM10 to the cell surface. HAECs were treated with or without insulin (100 nM) for 20 min. Cell surface proteins were labeled with biotin and isolated from intracellular proteins using avidin-coated agarose beads. Whole-cell lysates, intracellular protein fractions, and cell surface protein fractions were analyzed by Western blot using antibodies against ADAM10, Rab14, and actin. (n = 6 independent experiments, ^*^p < 0.05 vs. control).

Rab14 was detected in ADAM10 immunoprecipitates from untreated cells (Fig 9A). Insulin treatment for 20 min further increased the amount of Rab14 associated with ADAM10.

To examine the effects of insulin on the subcellular distribution of ADAM10 and Rab14, cell surface proteins were biotinylated and separated from intracellular proteins using avidin-coated agarose beads. Insulin treatment for 20 min significantly increased cell surface levels of both ADAM10 and Rab14, accompanied by a decrease in their intracellular levels (Fig 9B). In contrast, insulin did not alter the total cellular levels of ADAM10 or Rab14 (Fig 9B).

Together, these findings indicate that insulin promotes the translocation of both ADAM10 and Rab14 from intracellular compartments to the cell surface.

### 3.8. Rab14 is required for insulin-induced ADAM10 cell surface translocation

**Fig 10:**
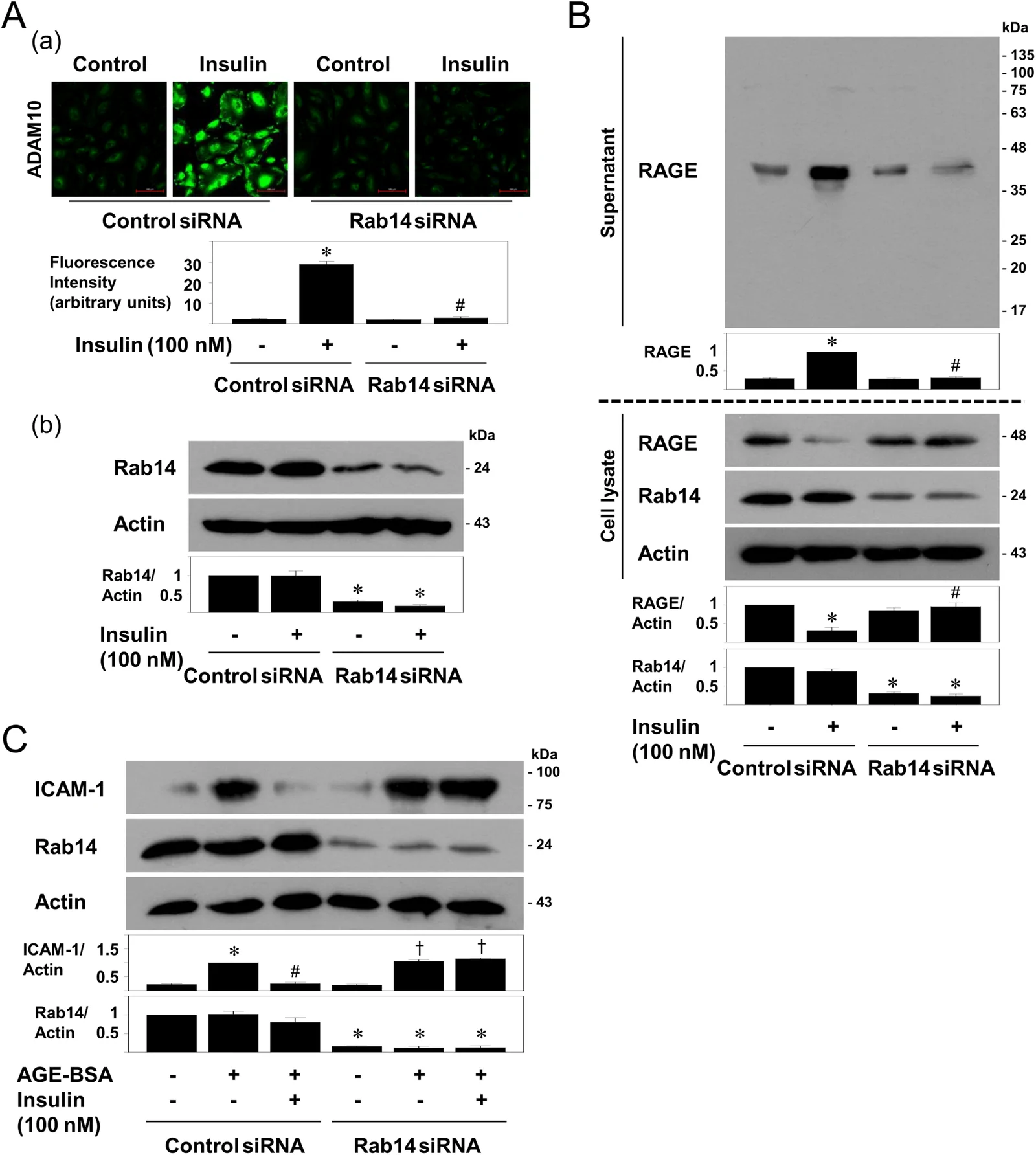
Rab14 is required for insulin-induced ADAM10 cell surface translocation. (A) Rab14 depletion abolishes insulin-induced ADAM10 cell surface translocation. HAECs were cultured on glass coverslips placed in cell culture dishes. Cells were transfected with control siRNA or Rab14 siRNA and then treated with or without 100 nM insulin for 20 min. (a) Cells grown on coverslips were subjected to immunofluorescence staining to assess cell surface ADAM10. Representative images and the relative fluorescence intensities are shown (scale bar, 100 μm). (b) Cells cultured in the same dishes but outside the coverslips were analyzed by Western blot to confirm Rab14 knockdown. (n = 3 independent experiments, ^*^p < 0.05 vs. control cells transfected with control siRNA; ^#^p < 0.05 vs. insulin-treated cells following control siRNA transfection). (B) Rab14 depletion abolishes insulin-induced RAGE ectodomain shedding. HAECs transfected with control siRNA or Rab14 siRNA were treated with or without 100 nM insulin for 30 min. Cell lysates and culture supernatants were analyzed by Western blot using antibodies against the RAGE extracellular domain, Rab14, and actin. (n = 3 independent experiments, ^*^p < 0.05 vs. control cells transfected with control siRNA; ^#^p < 0.05 vs. insulin-treated cells following control siRNA transfection). (C) Rab14 depletion abolishes the inhibitory effect of insulin on AGE-BSA-induced ICAM-1 expression. HAECs transfected with control siRNA or Rab14 siRNA were pretreated with or without 100 nM insulin for 30 min, followed by incubation with AGE-BSA (100 μg/mL) for 24 h. Cell lysates were analyzed by Western blot using antibodies against ICAM-1, Rab14, and actin. (n = 4 independent experiments, ^*^p < 0.05 vs. control; ^#^p < 0.05 vs. AGE-BSA; ^†^p < 0.05 vs. control cells transfected with Rab14-siRNA).

Finally, we investigated whether Rab14 is involved in insulin-induced ADAM10 cell surface translocation. To knock down Rab14 expression, HAECs were transfected with Rab14 siRNA. Rab14 knockdown attenuated insulin-induced ADAM10 cell surface translocation and RAGE ectodomain shedding (Fig 10A and 10B). In addition, Rab14 knockdown abolished the inhibitory effect of insulin on AGE-BSA-induced ICAM-1 expression (Fig 10C).

## 4. Discussion

Insulin enhances ADAM10-mediated RAGE ectodomain shedding, thereby attenuating cellular responses to AGEs; however, the underlying mechanism remains unclear. Although ADAM10 functions as a sheddase at the cell surface, it is predominantly retained in intracellular compartments under basal conditions. Here, we demonstrate that insulin stimulates ADAM10 translocation to the cell surface through a Rab14-dependent mechanism.

Insulin’s stimulatory effect on ADAM10 activity was previously demonstrated in Klotho-transfected COS-7 cells. Insulin treatment for 2 h enhanced ADAM10-mediated Klotho shedding without altering ADAM10 mRNA or protein levels [8]. Thus, insulin was suggested to increase ADAM10 shedding activity; however, the underlying mechanism remained unidentified. RAGE is also an established substrate of ADAM10. In the present study, insulin promoted RAGE ectodomain shedding, as demonstrated by decreased full-length RAGE levels in cell lysates, increased levels of the shed N-terminal fragment in culture supernatants, and accumulation of the C-terminal fragment in cell lysates. ADAM10 mediated insulin-induced RAGE ectodomain shedding, as insulin increased ADAM10 localization at the cell surface, whereas pharmacological inhibition or siRNA-mediated knockdown of ADAM10 abolished RAGE ectodomain shedding. The primary objective of this study was to elucidate the mechanism by which insulin enhances ADAM10 shedding activity. Insulin activated AKT, which was required for insulin-induced ADAM10 shedding activity, because pharmacological inhibition or siRNA-mediated knockdown of AKT suppressed insulin-induced ADAM10 cell surface translocation and RAGE ectodomain shedding. Although insulin did not alter total ADAM10 levels, it increased cell surface ADAM10 levels while reducing intracellular ADAM10 levels. These findings indicate that insulin enhances ADAM10 shedding activity by promoting AKT-dependent translocation of ADAM10 from intracellular compartments to the cell surface.

Rab14 is a member of the Rab GTPase family and participates in the cell surface translocation of glucose transporter 4, thereby facilitating glucose uptake at the plasma membrane [21]. Several studies have also suggested that Rab14 regulates ADAM10 trafficking to the cell surface. In a scratch wound assay using a cell monolayer, cells at the wound edge migrate into the denuded area, a process that requires ADAM10-mediated cleavage of the cell adhesion molecule N-cadherin. Under these conditions, Rab14 was shown to be essential for ADAM10 translocation to the cell surface [22]. We previously demonstrated that Rab14 is also required for AICAR- and SC79-induced ADAM10 cell surface trafficking, resulting in enhanced ADAM10 shedding activity [14,15]. In the present study, co-immunoprecipitation demonstrated an interaction between Rab14 and ADAM10. Although direct evidence of Rab14 activation would require demonstration of increased GTP loading or interaction with downstream effectors, our findings indicate that insulin enhances the Rab14–ADAM10 interaction while promoting the translocation of both proteins to the cell surface. Conversely, siRNA-mediated Rab14 knockdown inhibited insulin-induced ADAM10 cell surface translocation and RAGE ectodomain shedding, thereby attenuating the protective effect of insulin against AGE-BSA-induced endothelial activation.

The TspanC8 subgroup of tetraspanins, which includes Tspan5, Tspan10, Tspan14, Tspan15, Tspan17, and Tspan33, is recognized as a key regulator of ADAM10 trafficking [23]. TspanC8 proteins directly interact with ADAM10, regulate its exit from the ER [24], and promote its maturation in the Golgi apparatus [25]. Furthermore, TspanC8 proteins participate in the localization of mature ADAM10 to distinct subcellular compartments, causing it to adopt distinct conformations and cleave specific substrates [26]. In contrast, Rab14 is localized primarily to the trans-Golgi network and early endosomes [27]. In polarized epithelial cells, Rab14 regulates the trafficking of apical membrane proteins from the trans-Golgi network to the plasma membrane [28]. Therefore, Rab14 may contribute to the transport of ADAM10 from the trans-Golgi network to the cell surface. Collectively, TspanC8 proteins and Rab14 may have distinct but complementary roles in regulating ADAM10 trafficking.

AKT regulates Rab14 activity through phosphorylation of the Rab GTPase-activating proteins TBC1D1 and TBC1D4. Activated Rab14 subsequently facilitates membrane protein trafficking. AKT exists as three isoforms: AKT1, AKT2, and AKT3. In adipocytes and skeletal muscle cells, AKT2 is the predominant isoform responsible for TBC1D1 and TBC1D4 phosphorylation [29]. In neuronal cells, all three AKT isoforms contribute to TBC1D4 phosphorylation, with AKT2 showing the greatest contribution, followed by AKT3 and AKT1 [30]. In our previous study [15] and the present study in HAECs, siRNA-mediated depletion of AKT1, AKT2, or AKT3 inhibited SC79- or insulin-induced Rab14-dependent ADAM10 cell surface translocation. These findings suggest that all three AKT isoforms may contribute to this process. However, the observed effects may also reflect a reduction in overall AKT signaling following individual isoform knockdown, potential off-target effects of siRNA, or decreased pathway robustness [31]. Further studies examining the relationships among individual AKT isoforms, TBC1D1, TBC1D4, and Rab14 are required to clarify how AKT regulates Rab14-dependent ADAM10 trafficking.

Insulin signaling is primarily mediated through two major pathways: the insulin receptor substrate (IRS)/phosphatidylinositol 3-kinase (PI3K)/AKT pathway and the Src/mitogen-activated protein kinase (MAPK) pathway. The AKT pathway mediates many of the anti-atherogenic effects of insulin, whereas activation of the MAPK pathway is often associated with pro-atherogenic effects [4]. Insulin resistance is characterized by impaired AKT signaling accompanied by enhanced MAPK signaling [4]. Loss of insulin actions mediated through the AKT pathway contributes to the progression of atherosclerosis [4]. Our findings suggest that AKT may contribute to the anti-atherosclerotic effects of insulin, at least in part, by promoting ADAM10 translocation to the cell surface and facilitating the shedding of inflammation-related receptors such as RAGE. Consistent with this concept, endothelial ADAM10 has been shown to exert anti-atherosclerotic effects in a mouse model, and endothelial-specific deletion of ADAM10 markedly accelerated atherosclerotic plaque formation [32].

Healthy adults typically have fasting plasma insulin concentrations of approximately 0.025–0.07 nM, which can increase to approximately 0.3–0.8 nM following meal ingestion, depending on carbohydrate content and individual metabolic status [33,34]. In the present study, insulin was administered at 100 nM to achieve near-maximal activation of downstream insulin signaling pathways, representing a supraphysiological concentration commonly used in mechanistic cell culture studies. However, in dose-response experiments, insulin reduced full-length RAGE levels at concentrations as low as 1 nM and significantly inhibited AGE-BSA-induced ICAM-1 expression at the same concentration. Although 1 nM insulin remains higher than the typical peripheral postprandial insulin concentrations in healthy individuals, it is considerably closer to the physiological range than 100 nM. Furthermore, the effective insulin exposure experienced by cultured cells may differ from the nominal concentration because of factors such as degradation or adsorption to culture vessels, as well as the sustained nature of in vitro exposure compared with the dynamic fluctuations in circulating insulin observed *in vivo* [35–37]. Therefore, the observed effects are not restricted to highly supraphysiological insulin concentrations and may also occur at insulin concentrations closer to those encountered under physiological conditions.

There are several limitations to this study. First, we did not evaluate the role of PI3K in insulin-induced ADAM10 trafficking and RAGE ectodomain shedding. Given that PI3K is a major upstream regulator of AKT, further investigation of its role in this process is warranted to strengthen our conclusions. Second, surface protein biotinylation experiments ideally require purity controls for the membrane and cytosolic fractions, such as Na⁺/K⁺-ATPase and GAPDH, to confirm successful fractionation and exclude cross-contamination. These controls were not included in the present study. Third, to further establish the anti-inflammatory relevance of our findings, the effect of insulin on AGE-BSA-induced endothelial activation was assessed solely by measuring ICAM-1 expression. Future studies assessing additional markers of endothelial activation, including interleukin-6, E-selectin, vascular cell adhesion molecule-1, and monocyte chemoattractant protein-1, would further strengthen the biological significance of our findings.

## 5. Conclusion

**Fig 11:**
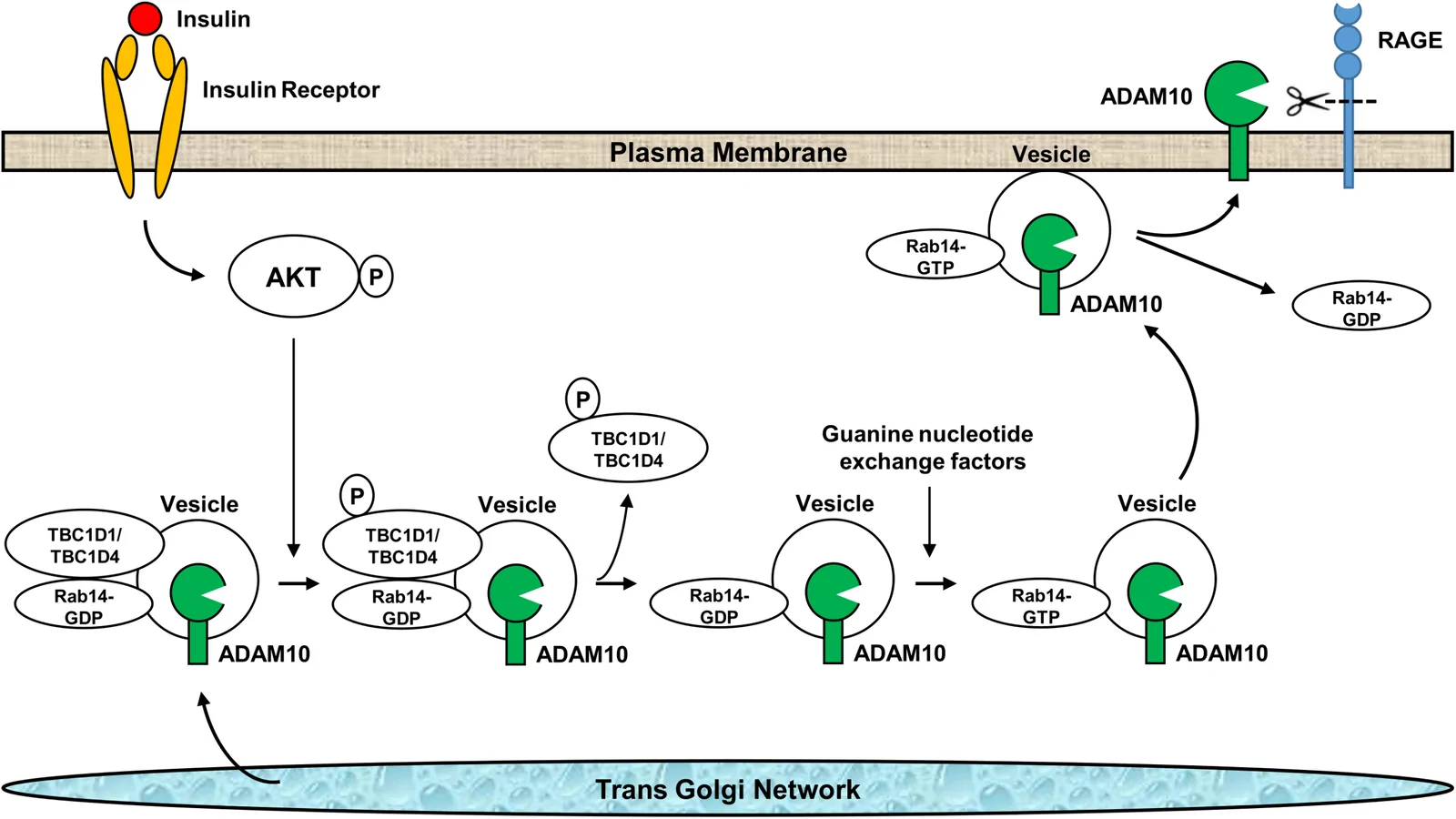
Proposed schematic model of insulin signaling-mediated Rab14-dependent ADAM10 trafficking and RAGE ectodomain shedding in human aortic endothelial cells. Based on existing literature, we propose that TBC1D1 and TBC1D4 may function as Rab GTPase-activating proteins that negatively regulate Rab14, thereby controlling the trafficking of ADAM10-containing vesicles originating from the trans-Golgi network. Upon insulin stimulation, AKT is activated and subsequently phosphorylates TBC1D1 and TBC1D4, thereby reducing their inhibitory effect on Rab14. Rab14 can then be activated by its guanine nucleotide exchange factors, such as DENND6A and DENND6B, promoting the translocation of ADAM10-containing vesicles to the plasma membrane, where ADAM10 cleaves multiple substrates, including RAGE.

The present study demonstrates that insulin promotes AKT-dependent, Rab14-mediated trafficking of ADAM10 to the cell surface in HAECs, thereby enhancing RAGE ectodomain shedding and attenuating AGE-induced endothelial activation (Fig 11). These findings identify a previously unrecognized mechanism by which insulin regulates ADAM10 trafficking and provide new insights into the anti-inflammatory and anti-atherosclerotic actions of insulin in the vascular endothelium.

## Supporting information

S1 Fig Western blot images showing the effect of insulin on AKT, Rab14, ADAM10, RAGE, and ICAM-1 expression. (PDF)

S2 Fig Immunofluorescence staining images showing the effect of insulin on the cell surface expression of ADAM10. (PDF)

S1 Data Densitometry data corresponding to the Western blot bar graphs. (XLSX)

S2 Data Densitometry data corresponding to the immunofluorescence staining bar graphs. (XLSX)
