# Supplementary material for: Insulin enhances RAGE ectodomain shedding by inducing Rab14-dependent ADAM10 cell surface trafficking in human aortic endothelial cells
Source: PLoS One. 2026 Sep 18;21(9):e0358445. doi: 10.1371/journal.pone.0358445 (PMC13588500; doi:10.1371/journal.pone.0358445)
Supplement: Supplementary file 1 [file pone.0358445.s001.pdf]

# Insulin enhances RAGE ectodomain shedding by inducing Rab14-dependent ADAM10 cell surface trafficking in human aortic endothelial cells

Chung Hee Baek[a,1], Hyosang Kim[a,1], Soo Young Moon[b],
Eun Kyoung Lee[c], Won Seok Yang[a,*]

**- Western Blot Images -**

# Figure 1

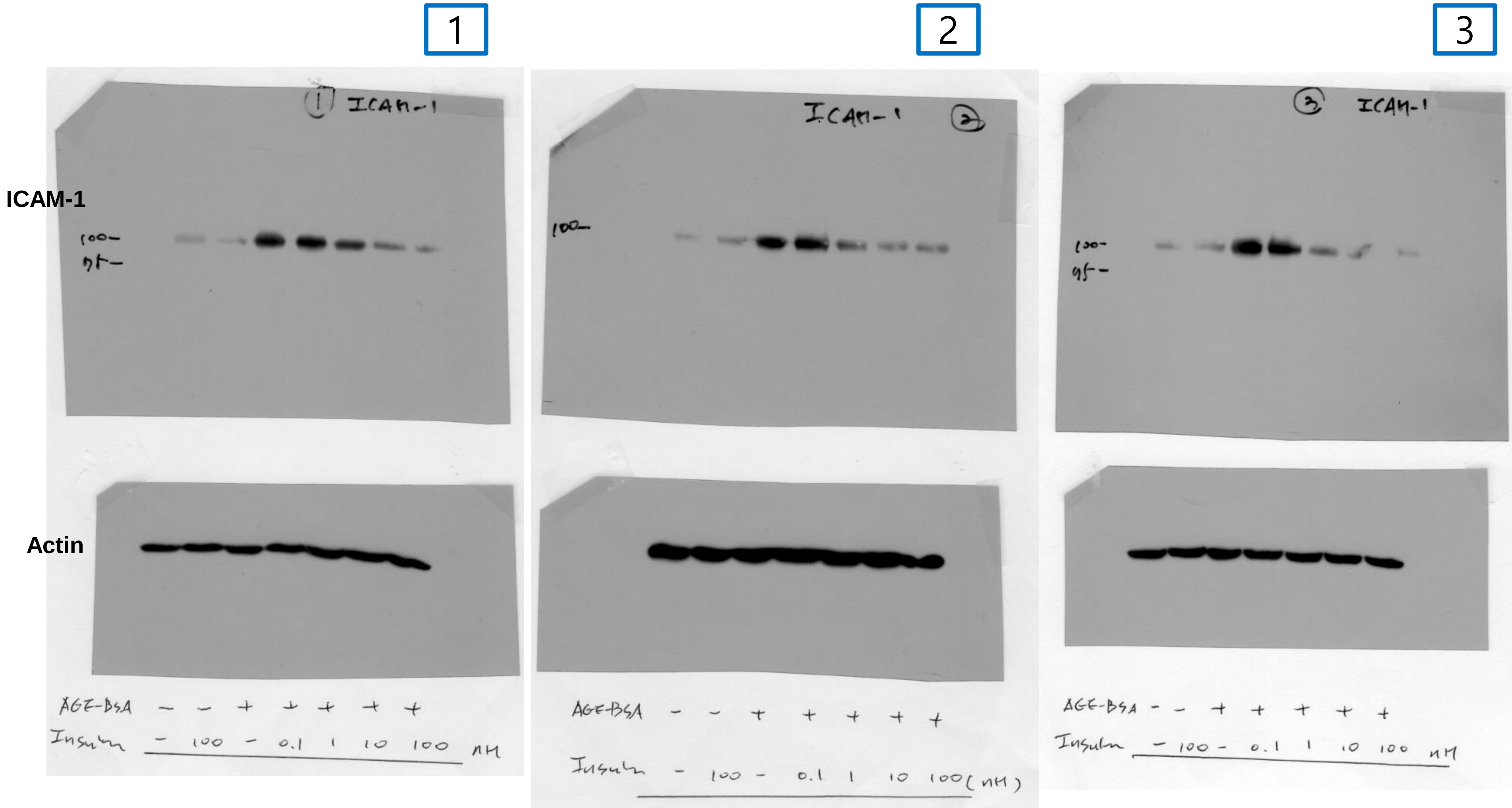

Figure 2A1

1

RAGE

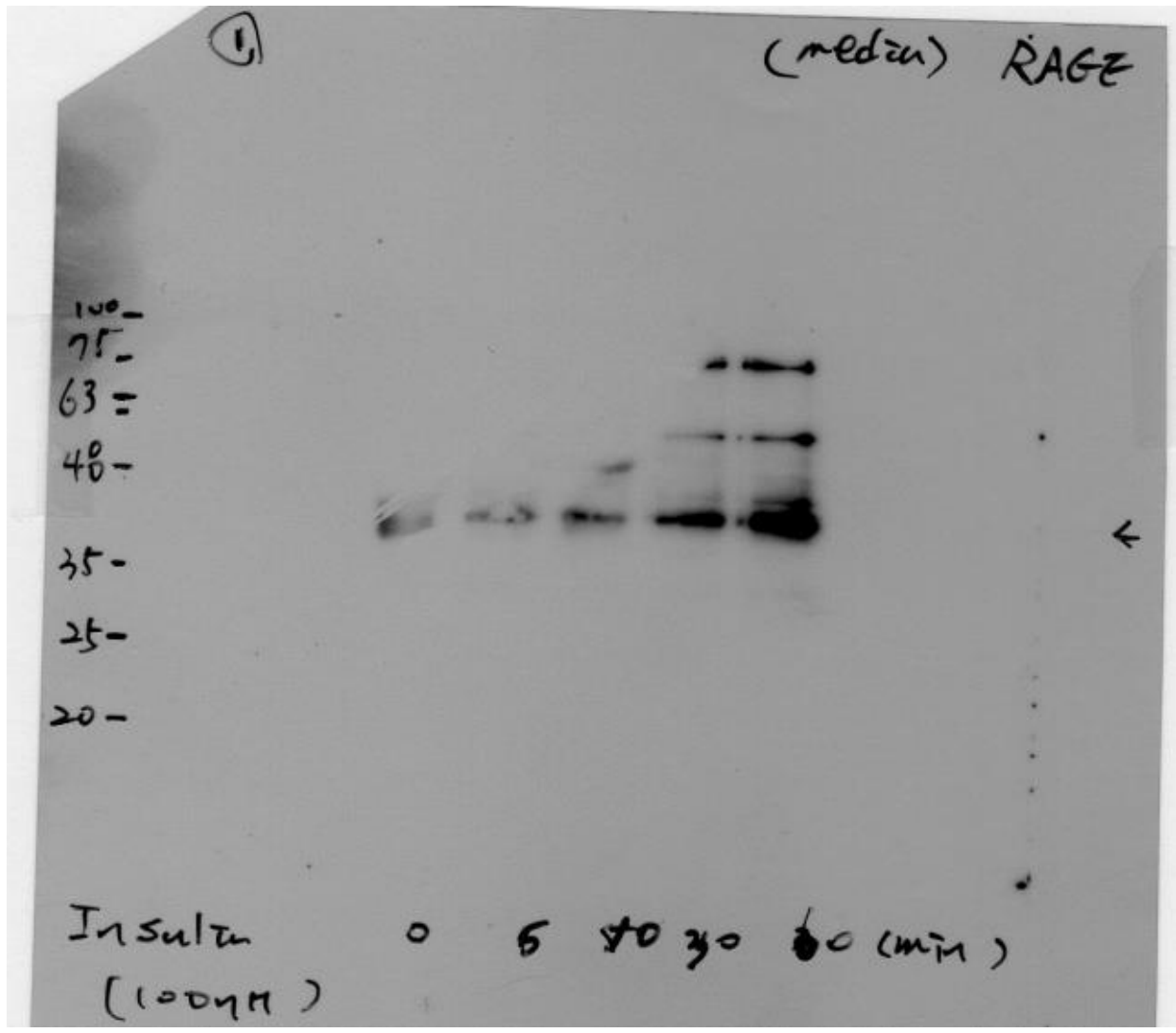


2

RAGE

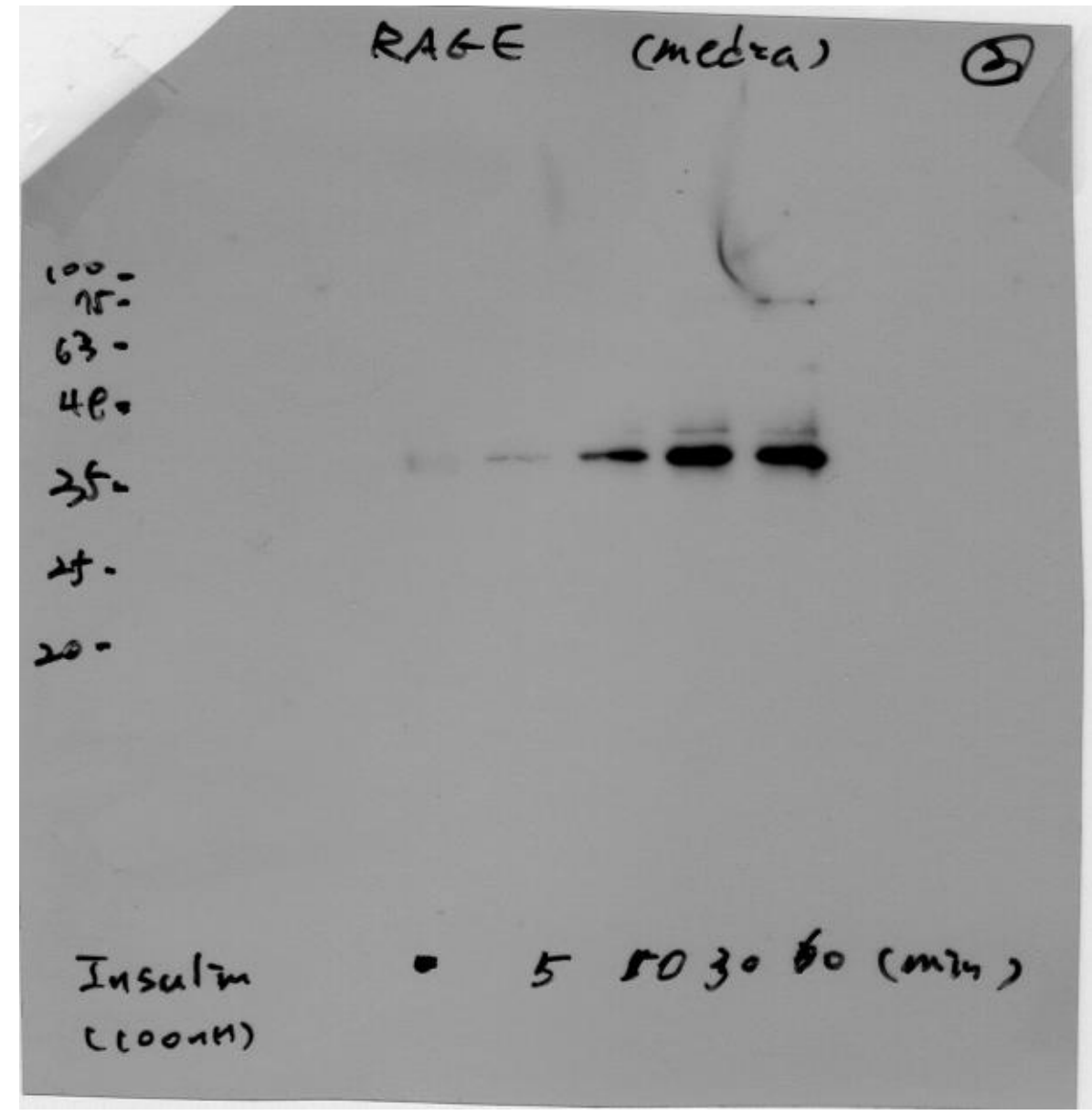


3

RAGE

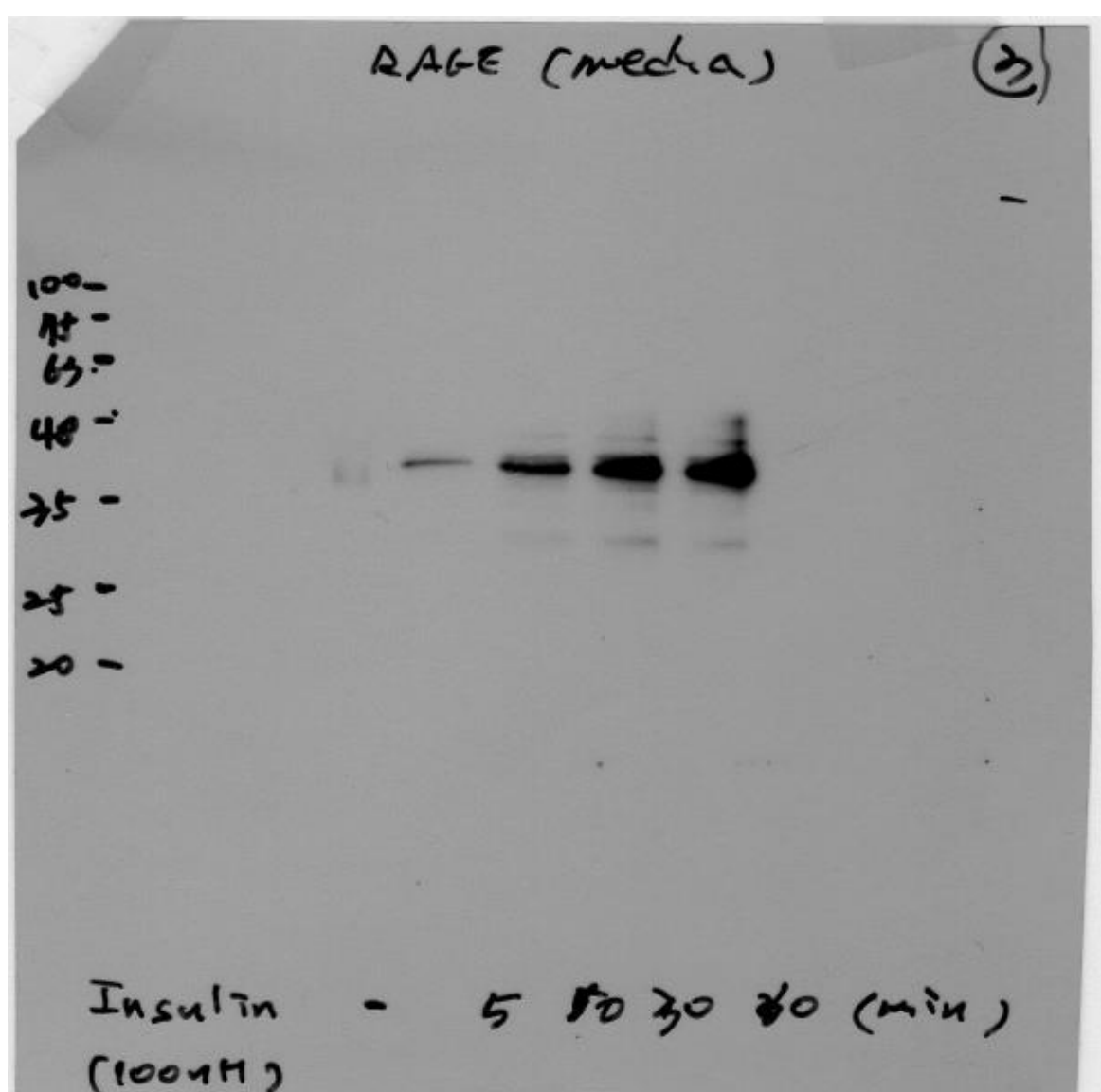

Figure 2A2

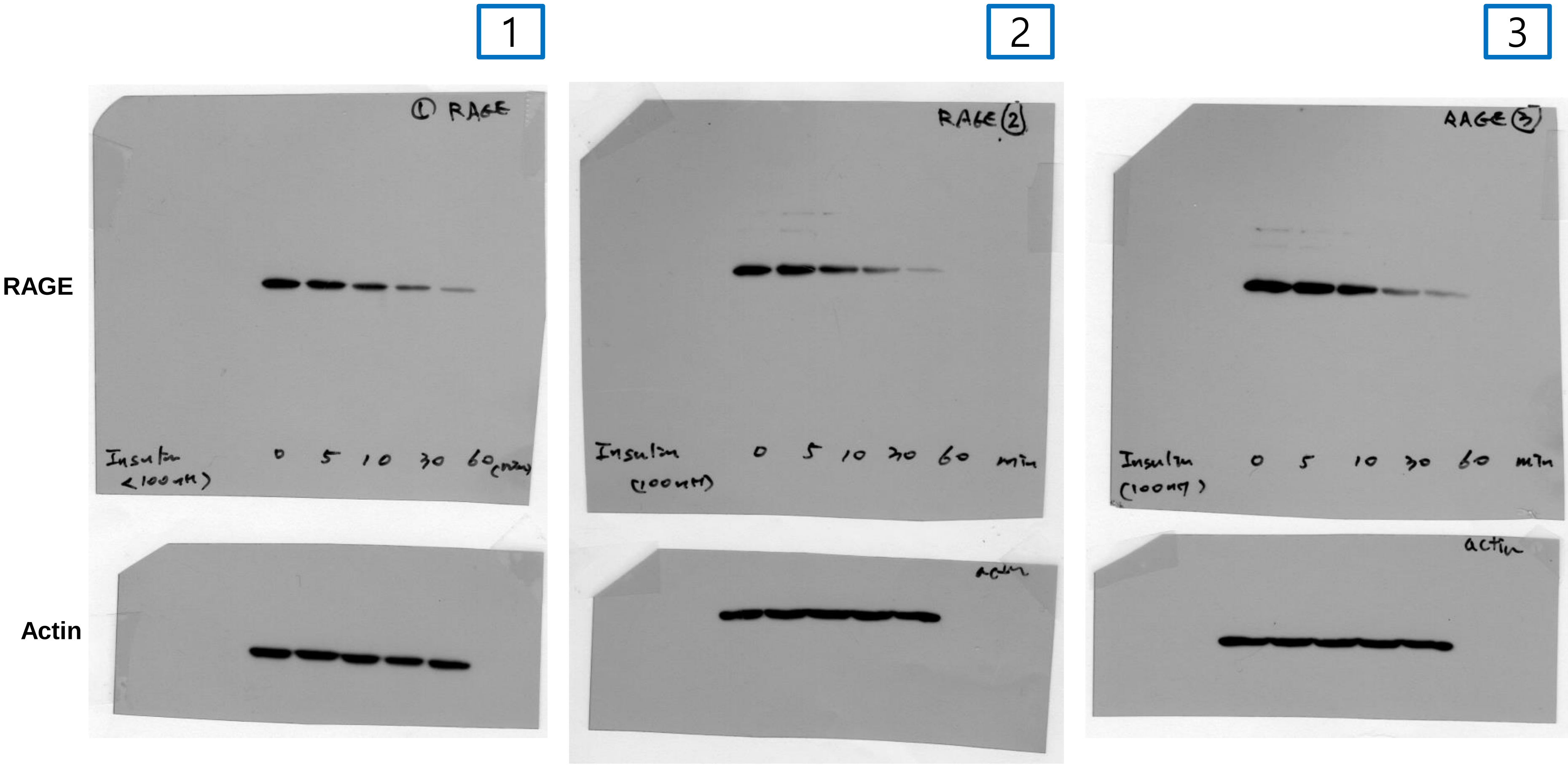
1
2
3
RAGE
Actin
RAGE
Insulin
0 5 10 30 60
RAGE
Insulin (100nM)
0 5 10 30 60 min
actin
RAGE
Insulin (100nM)
0 5 10 30 60 min
actin

Figure 2B1

RAGE

1

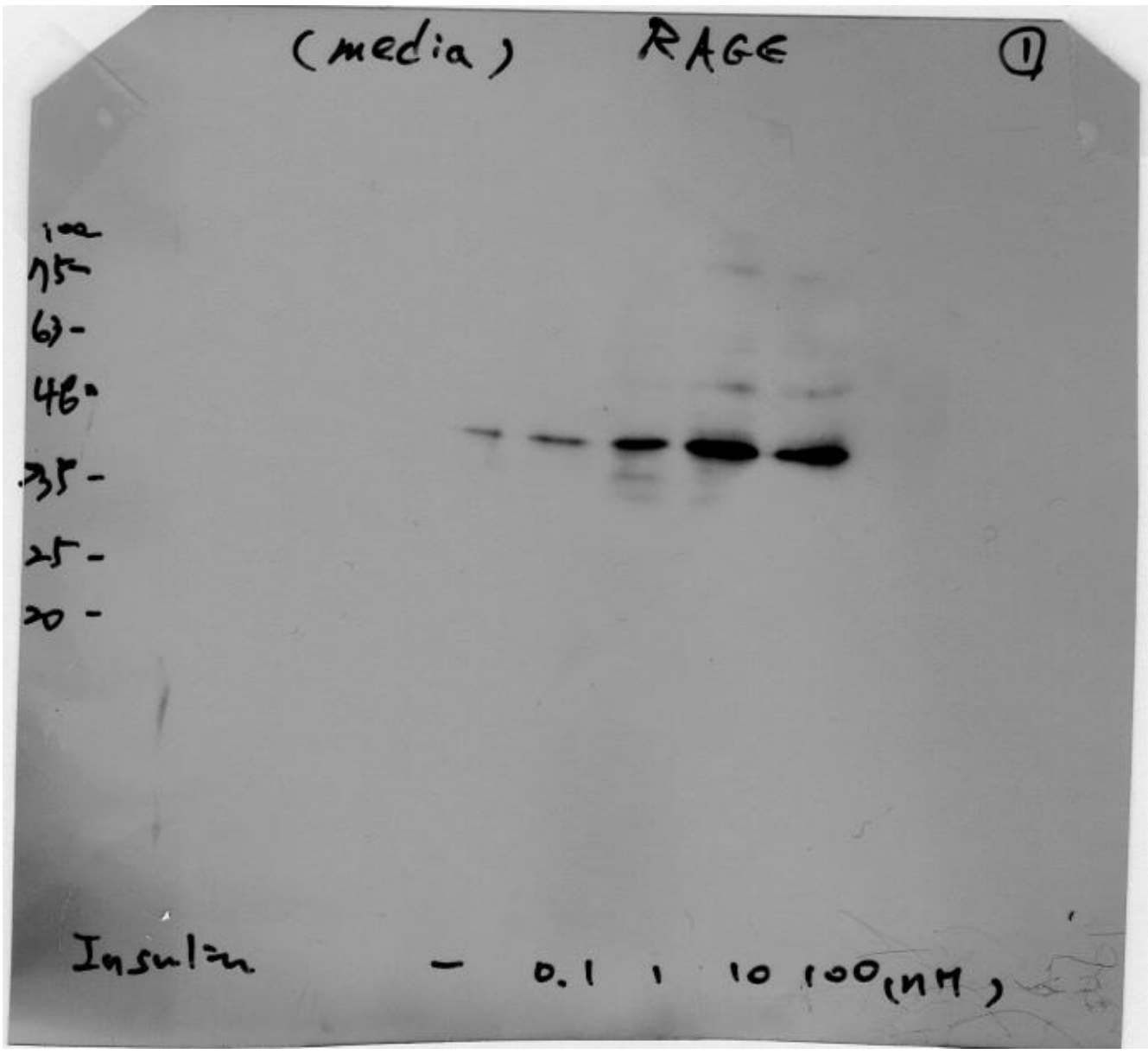


2

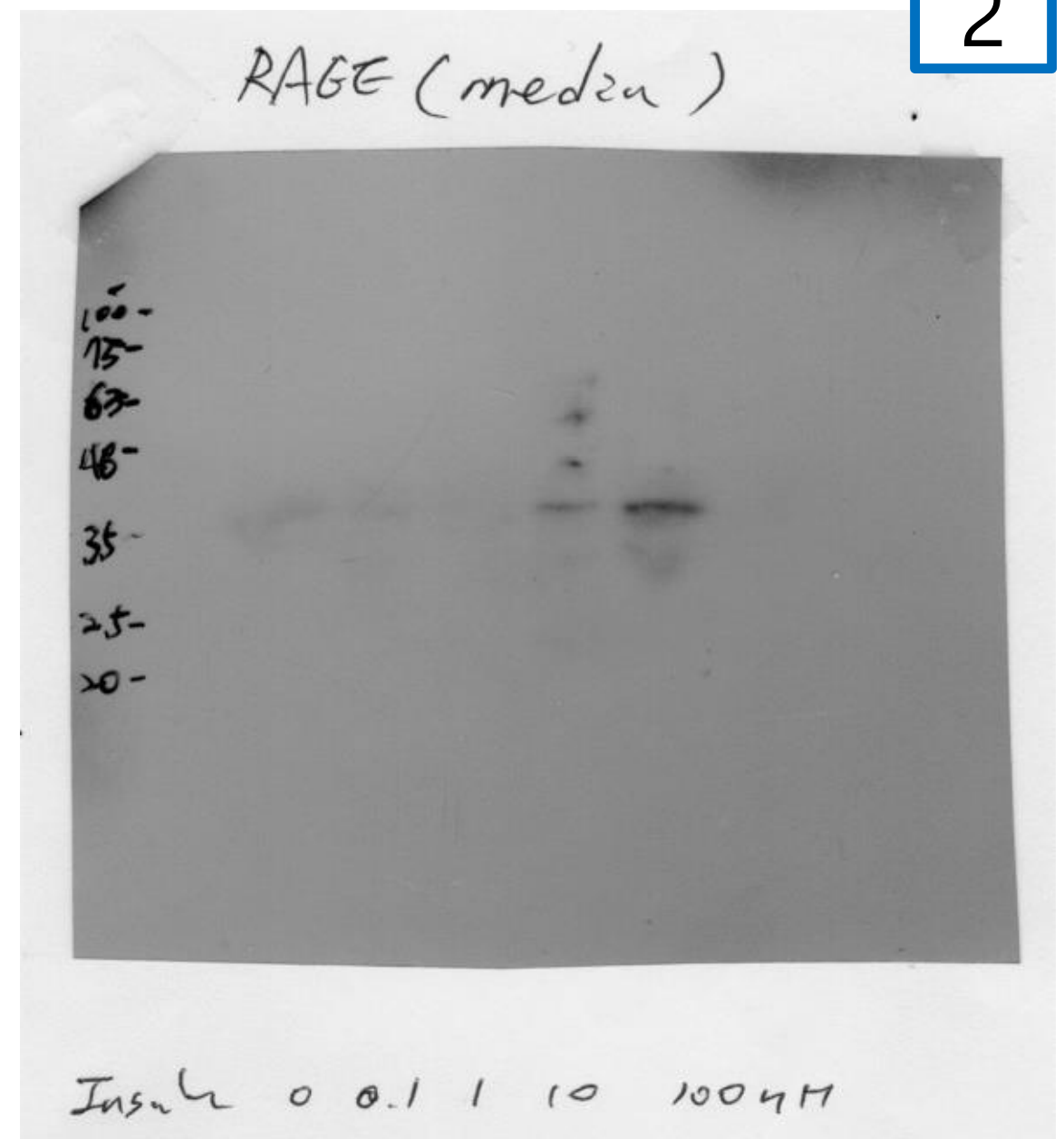


3

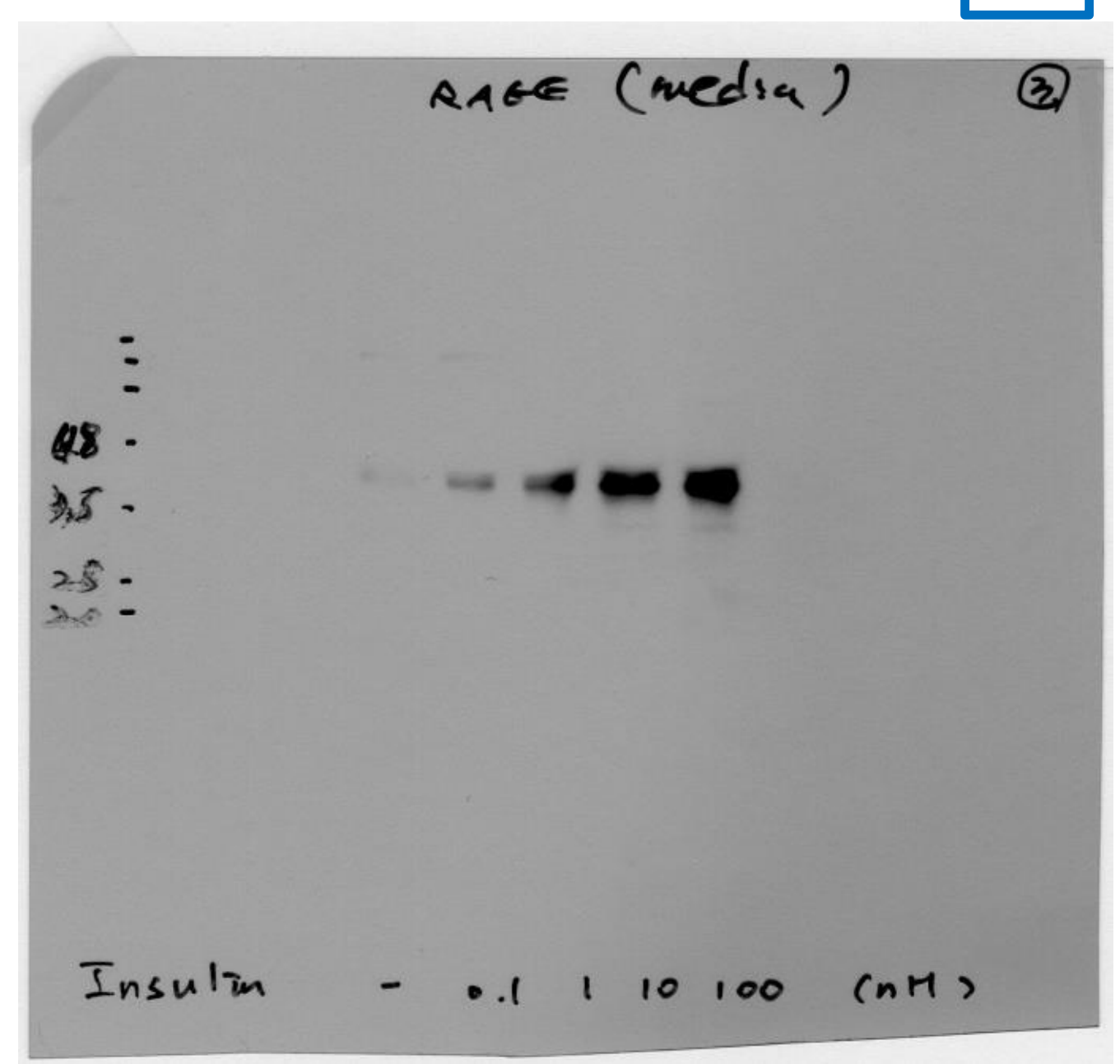

Figure 2B2

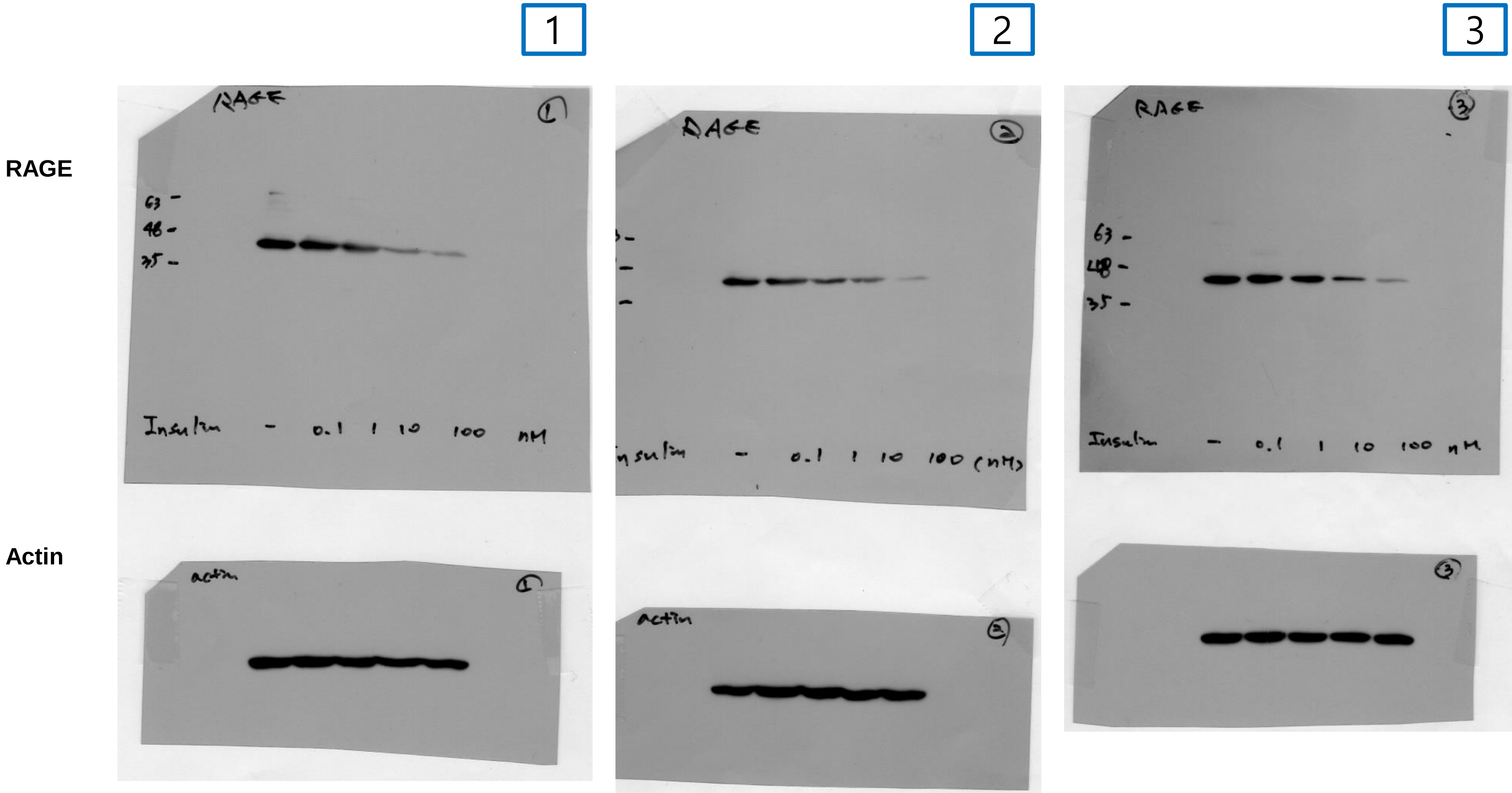

1
2
3
RAGE
Actin
RAGE
63
48
35
Insulin - 0.1 1 10 100 nM
Insulin - 0.1 1 10 100 (nM)
Insulin - 0.1 1 10 100 nM
actin
Actin

# Figure 2C

Full-length RAGE

C-terminal fragmen of RAGE

Actin

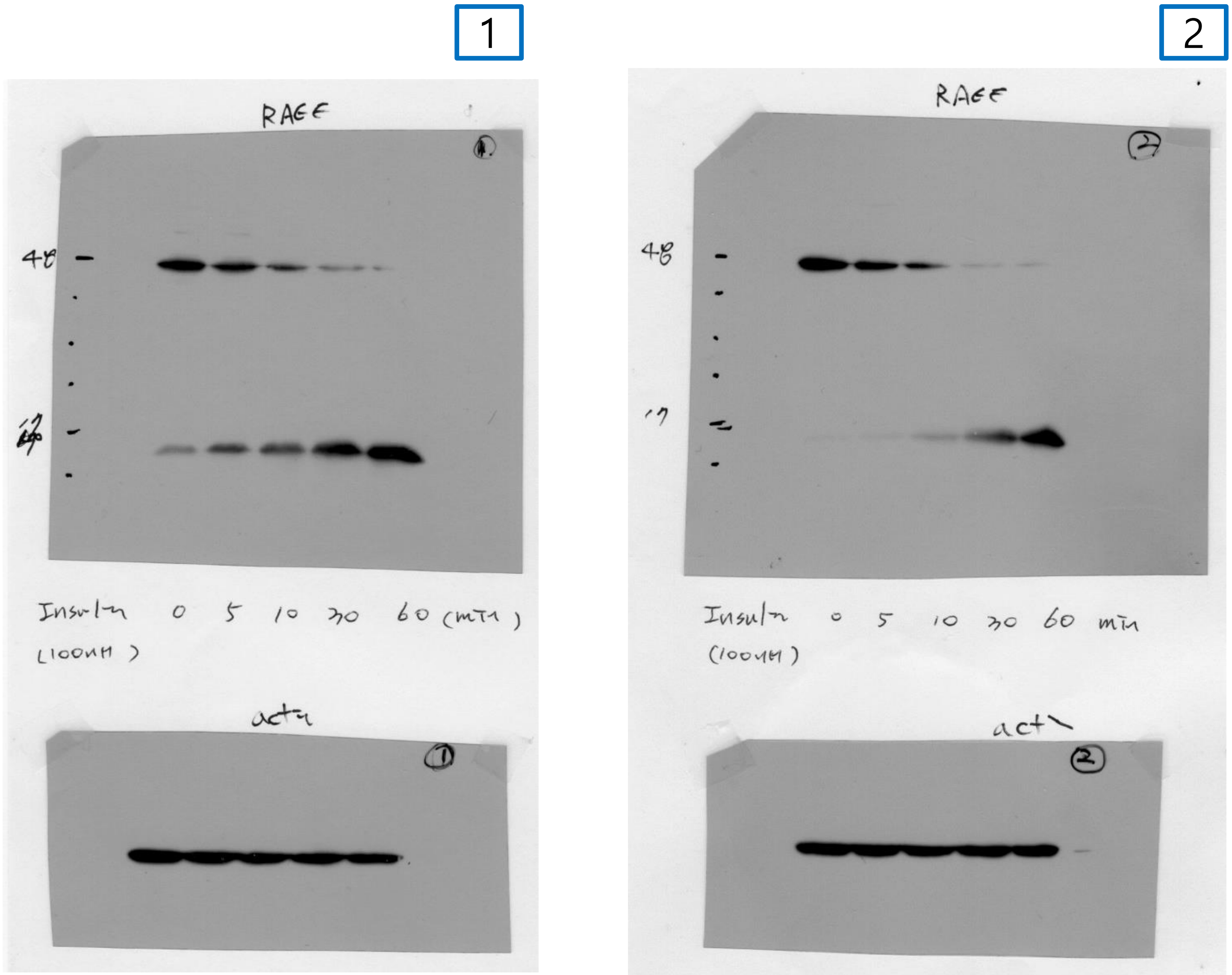

Figure 2C

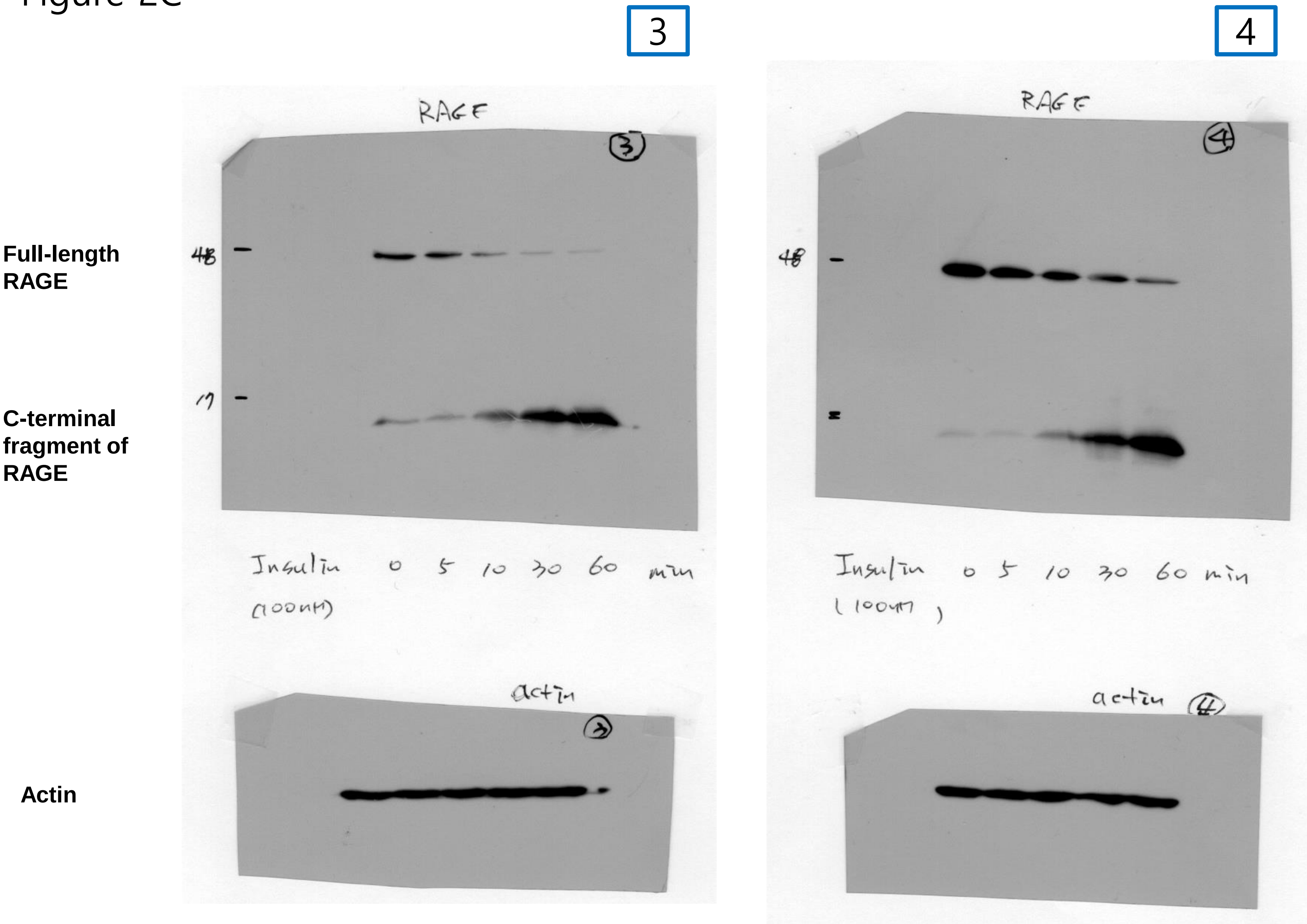
3
4
Full-length RAGE
C-terminal fragment of RAGE
Actin
RAGE
48
17
Insulin (100nM) 0 5 10 30 60 min
actin

Figure 2D

1

2

Full-length RAGE

C-terminal fragmen of RAGE

Actin

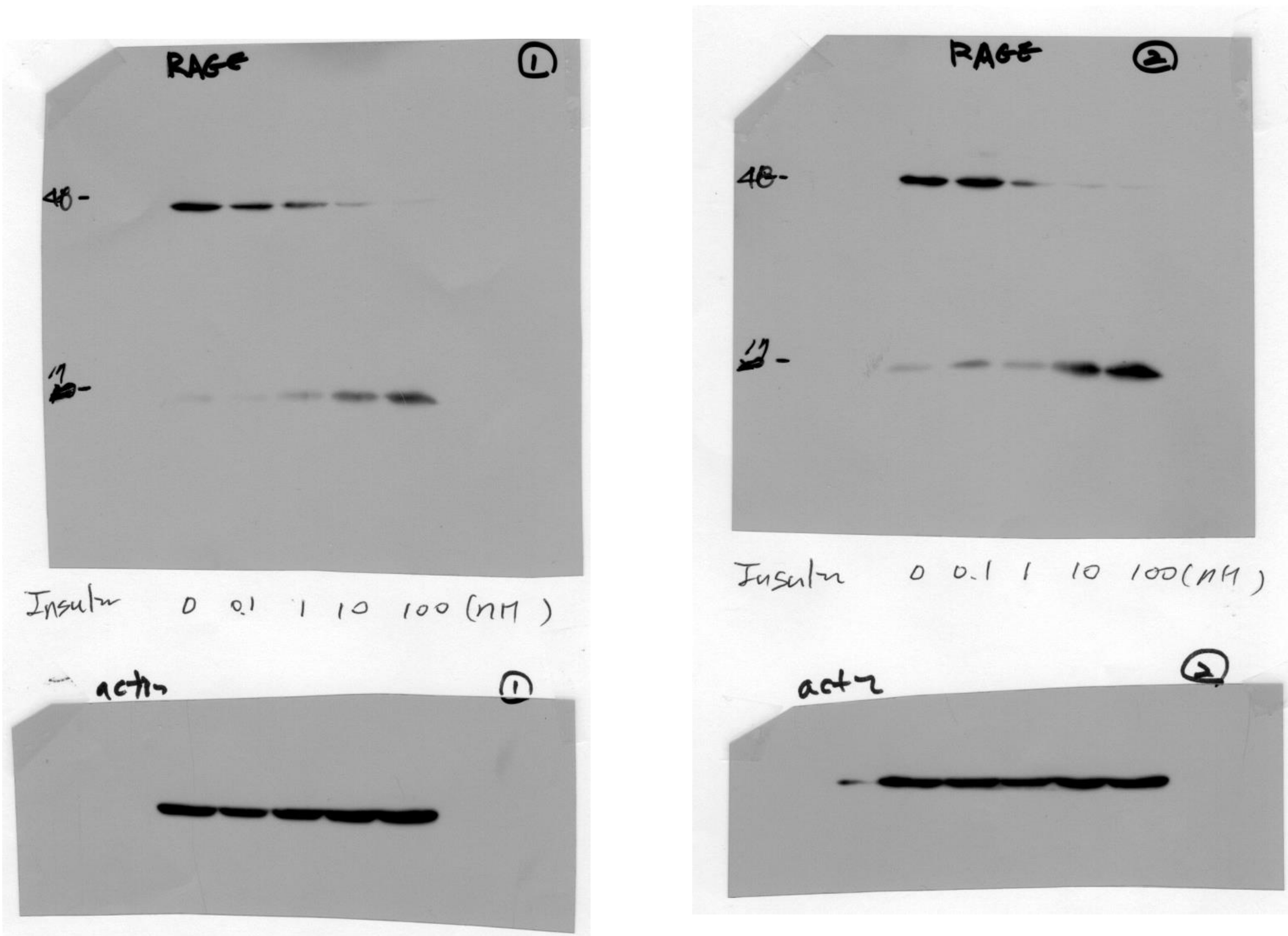

Figure 2D

**Full-length RAGE**

**C-terminal fragment of RAGE**

**Actin**

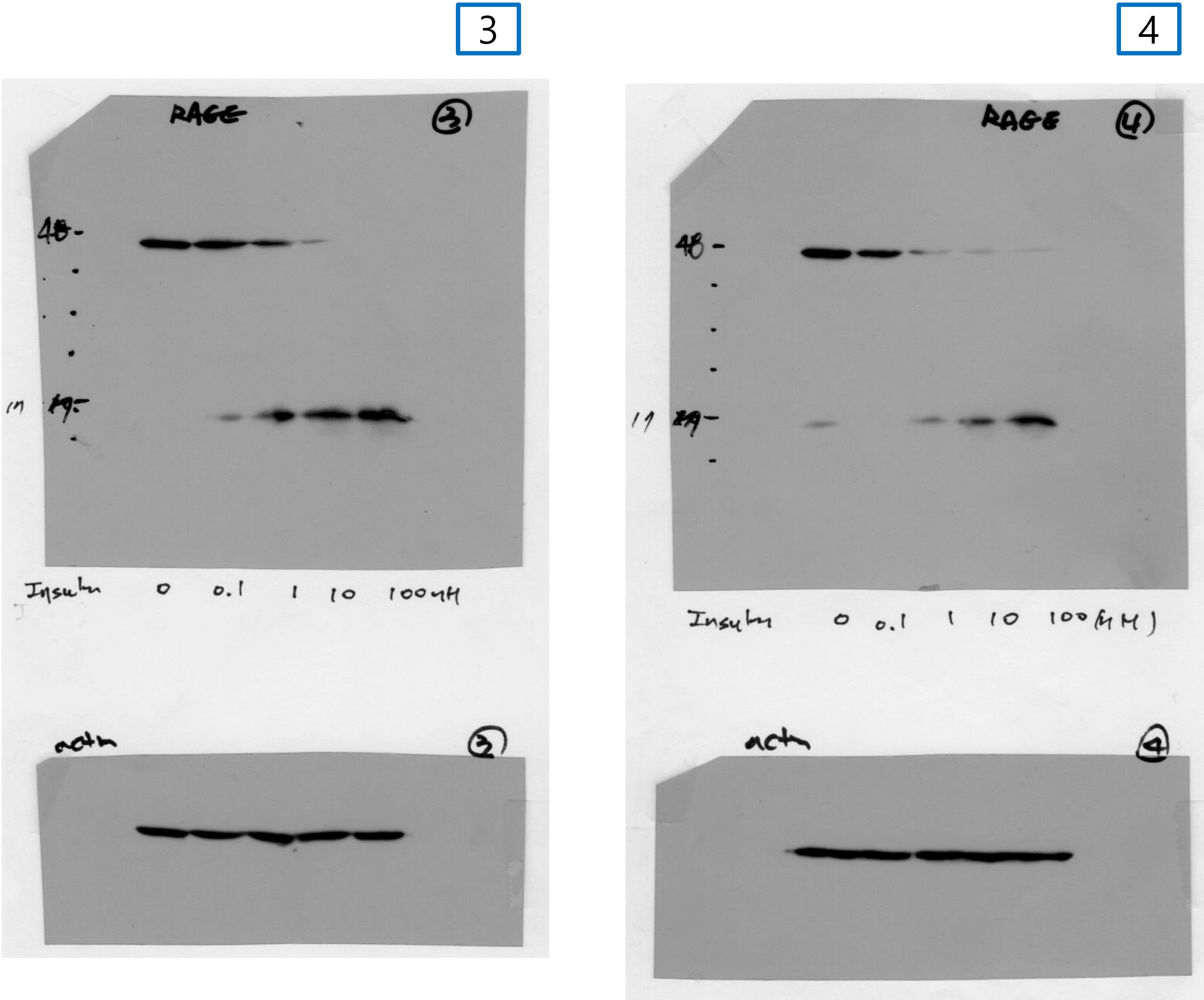

# Figure 3 – upper panel

1

2

3

RAGE

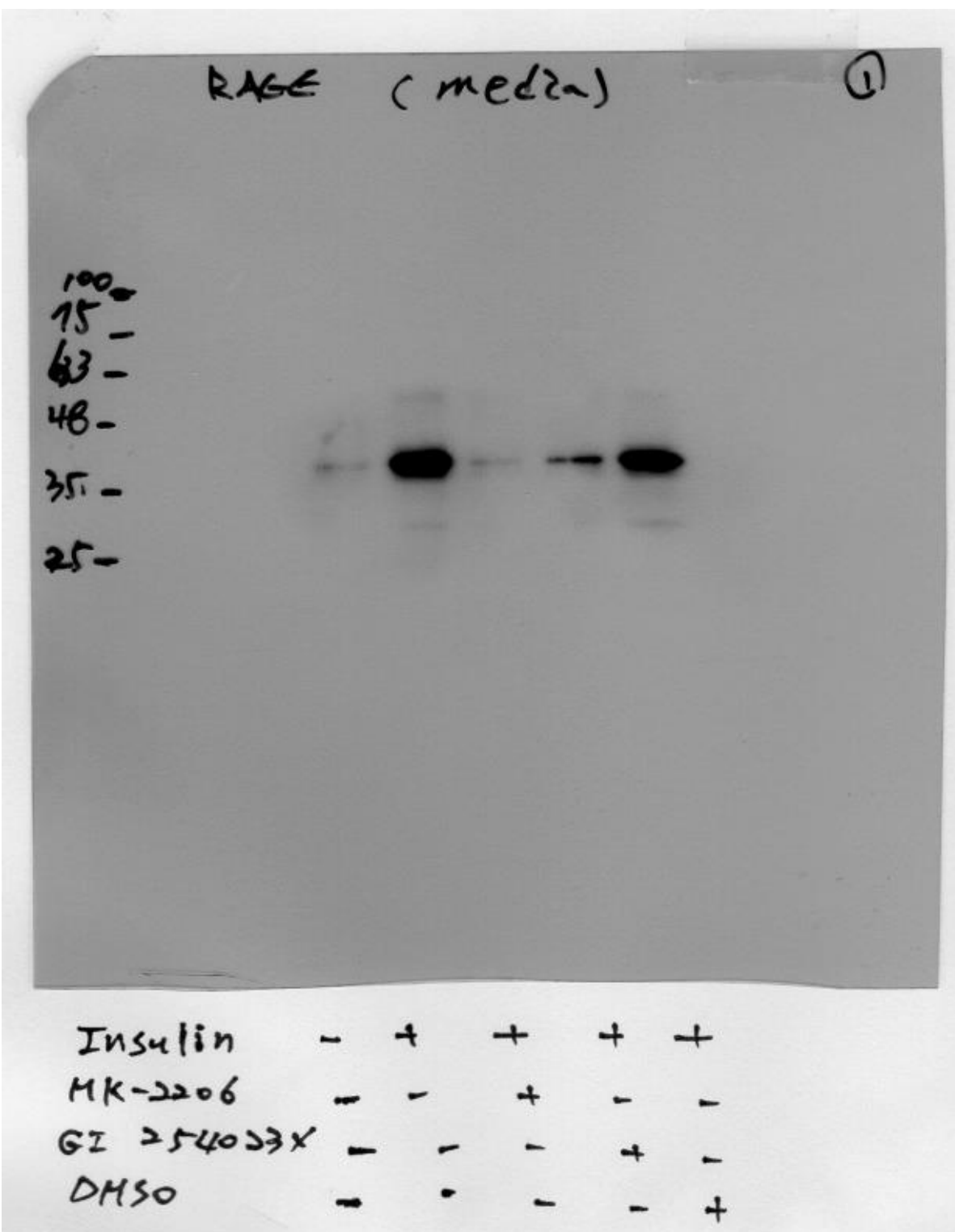


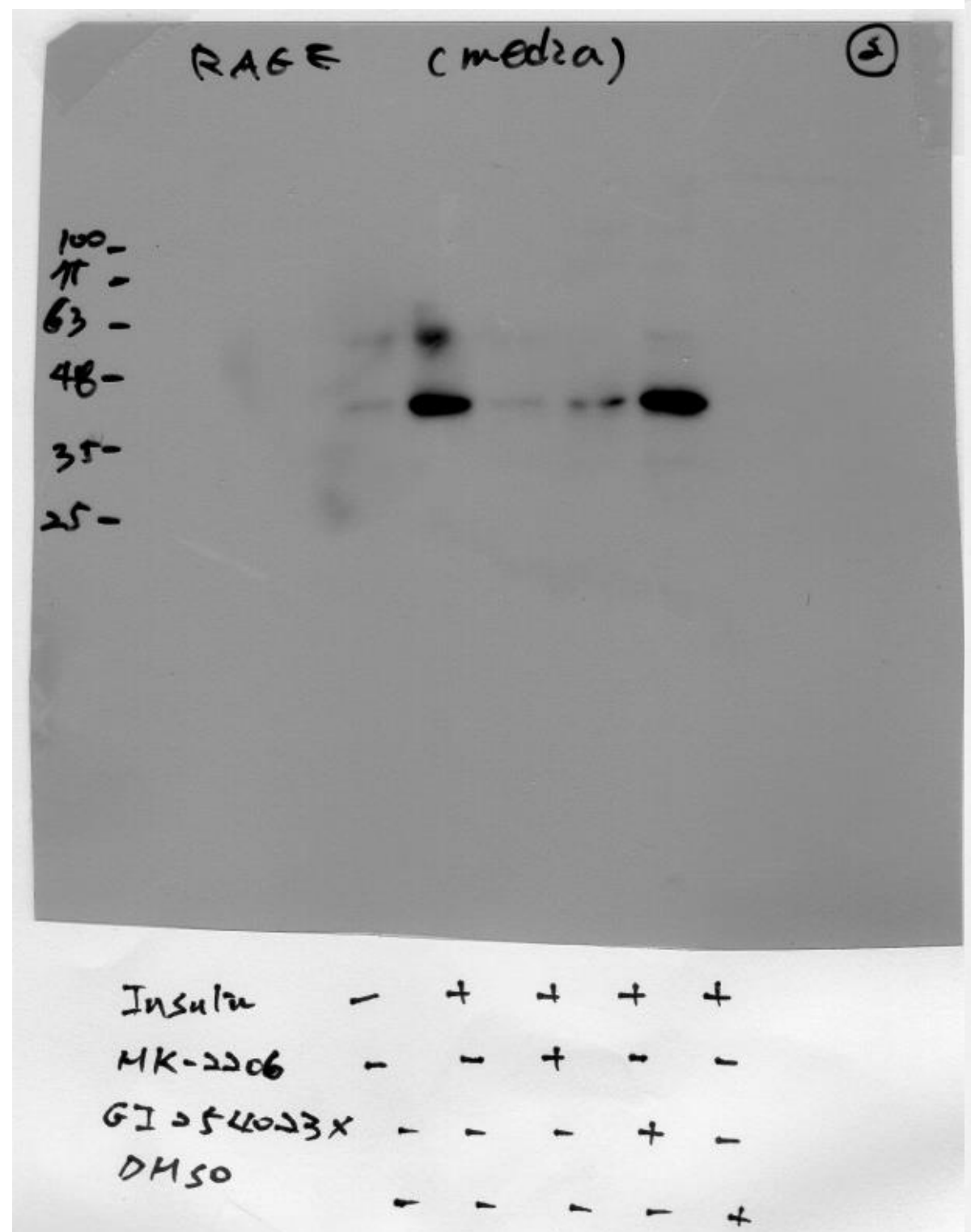


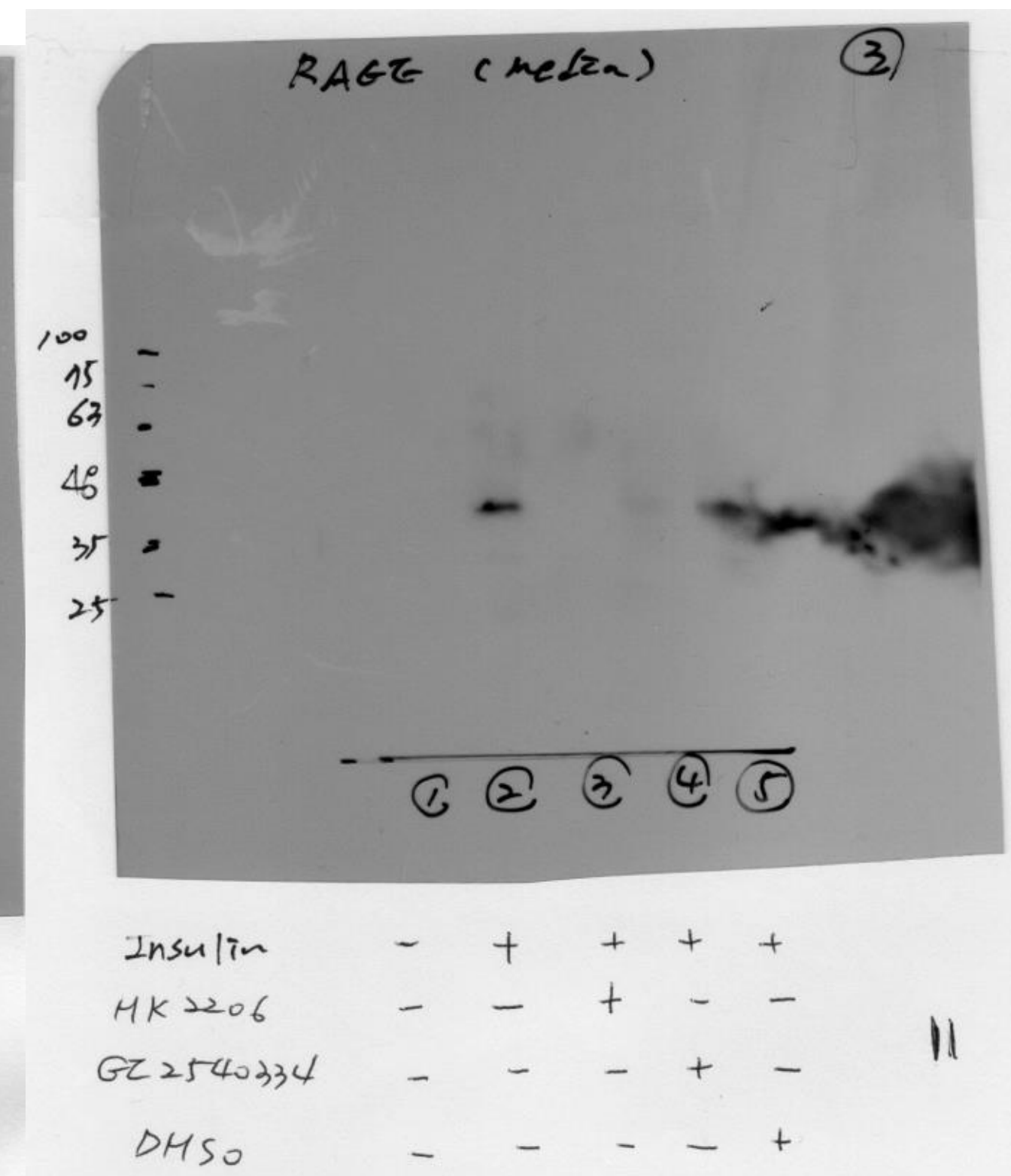

# Figure 3 – lower panel

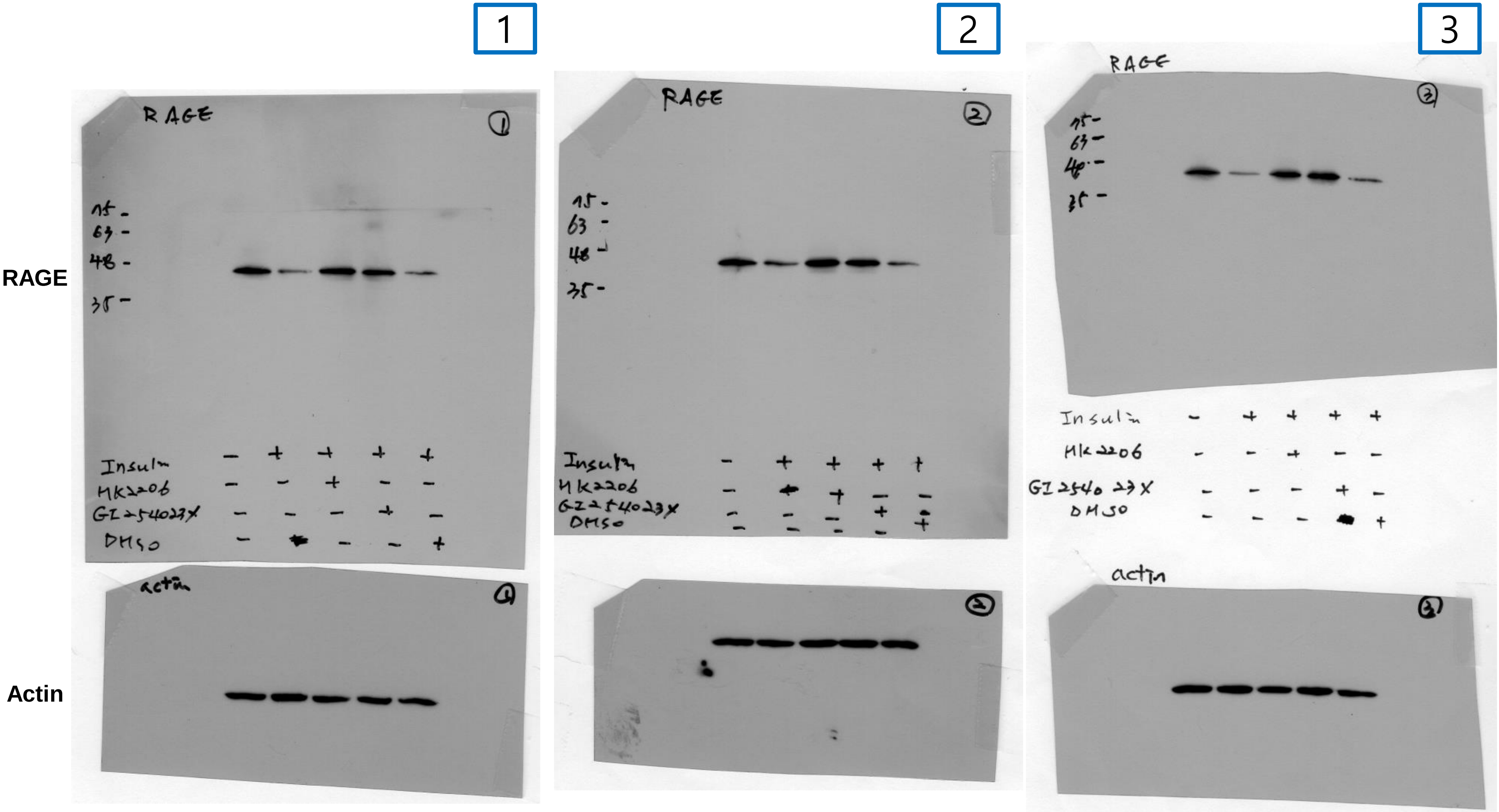

Figure 4A upper panel

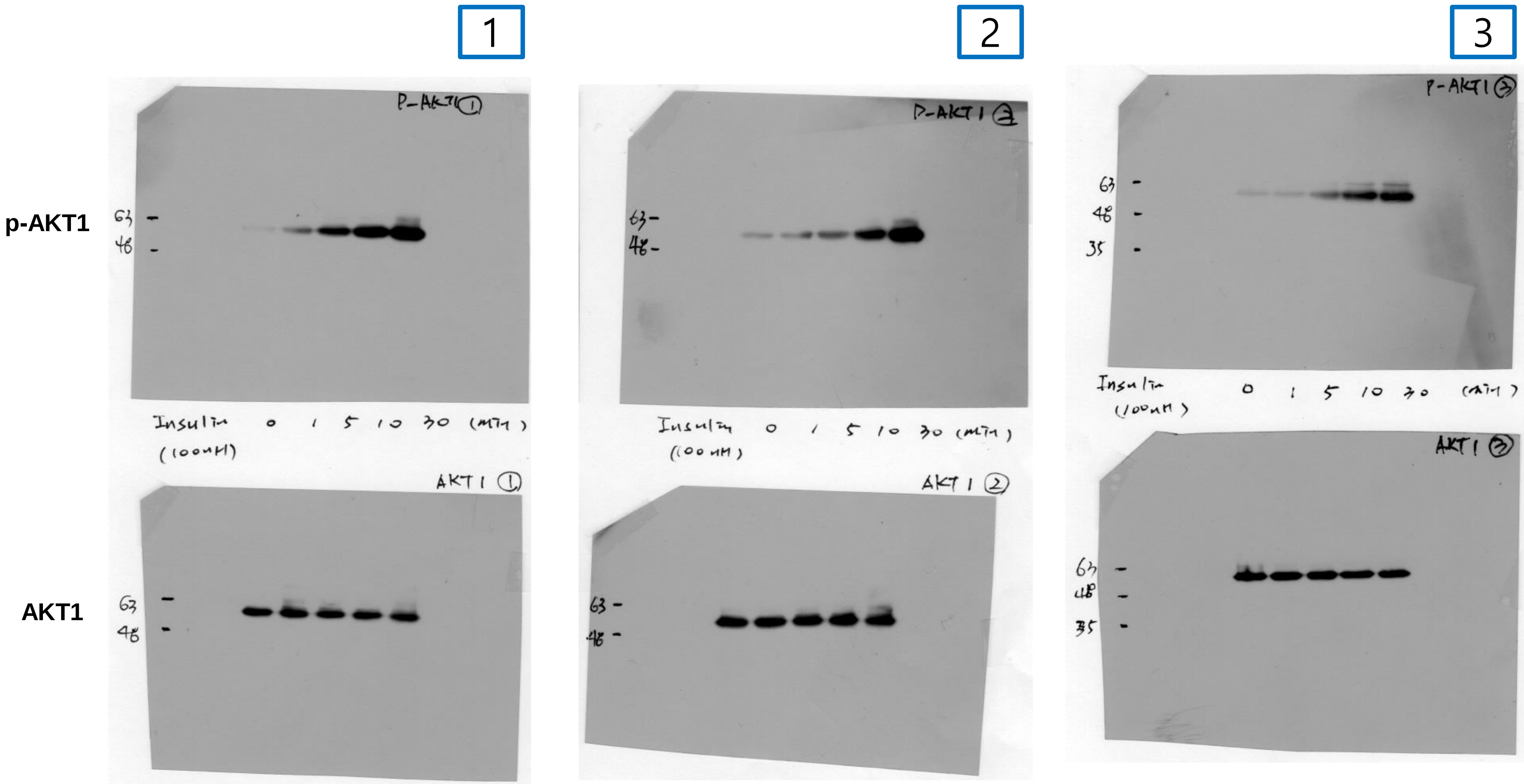

# Figure 4A lower panel

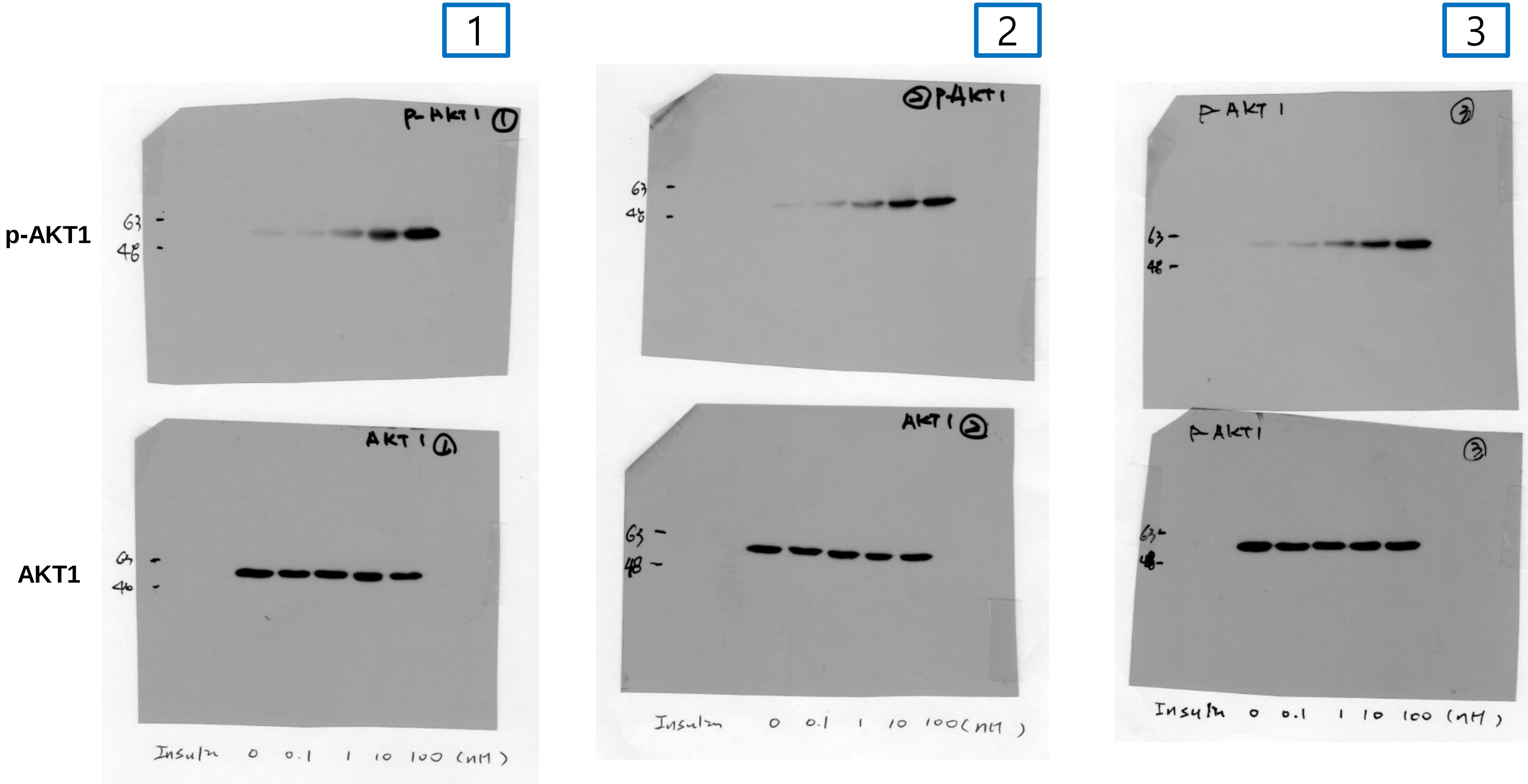

Figure 4B upper panel

1

2

3

RAGE

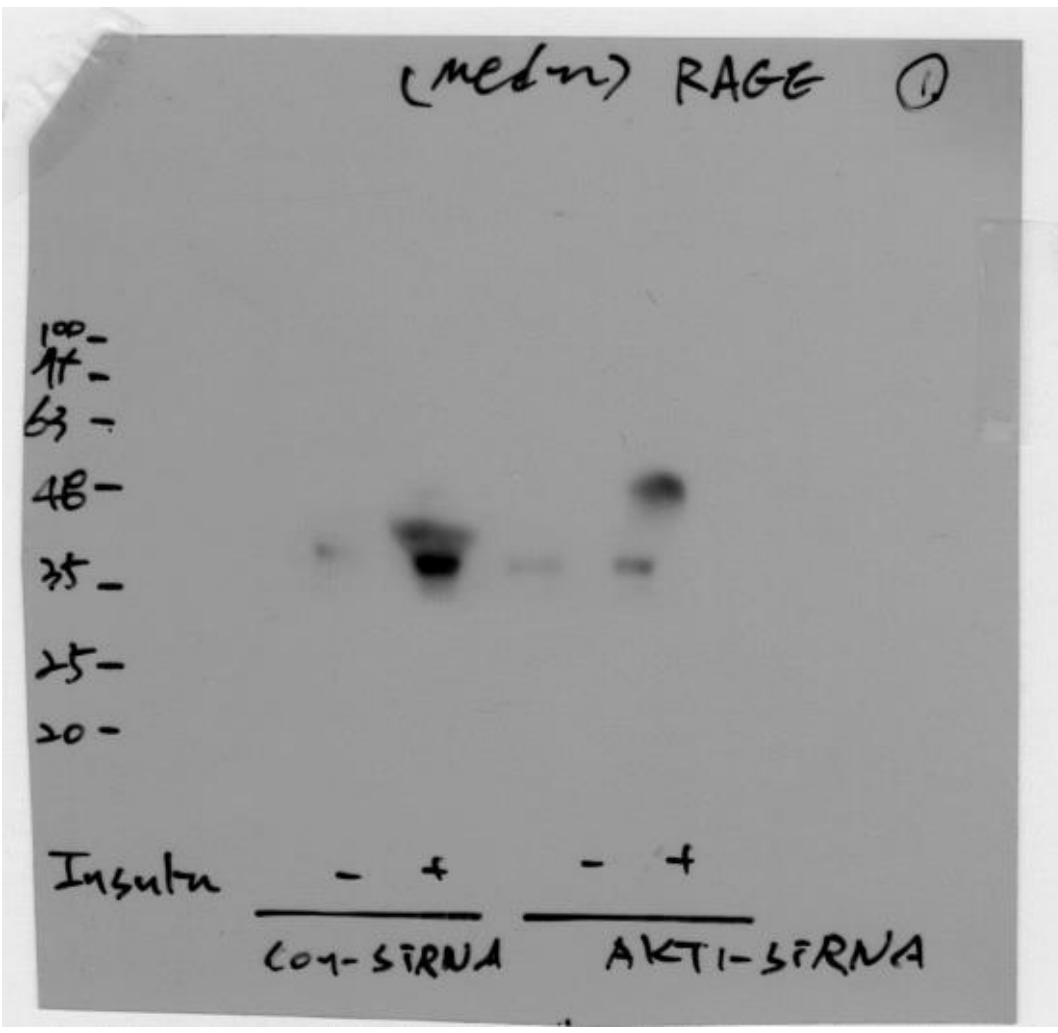


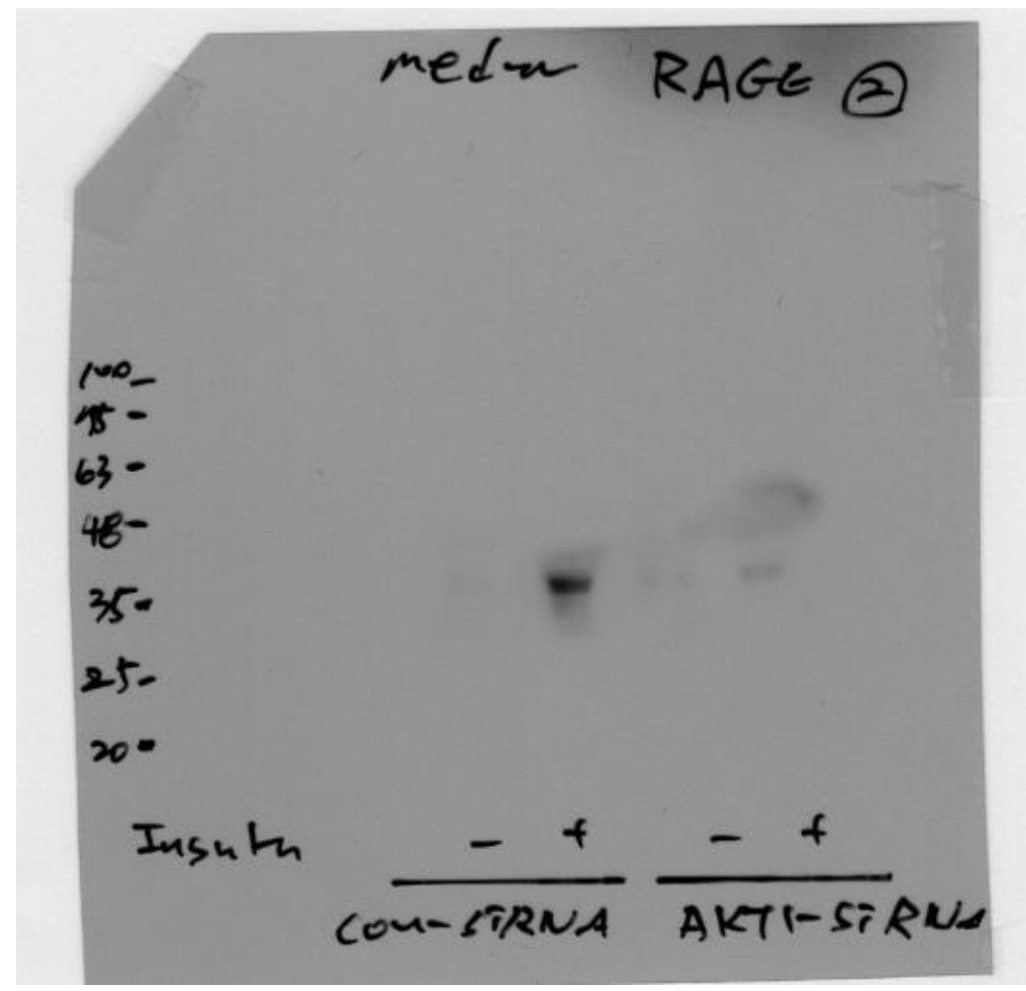


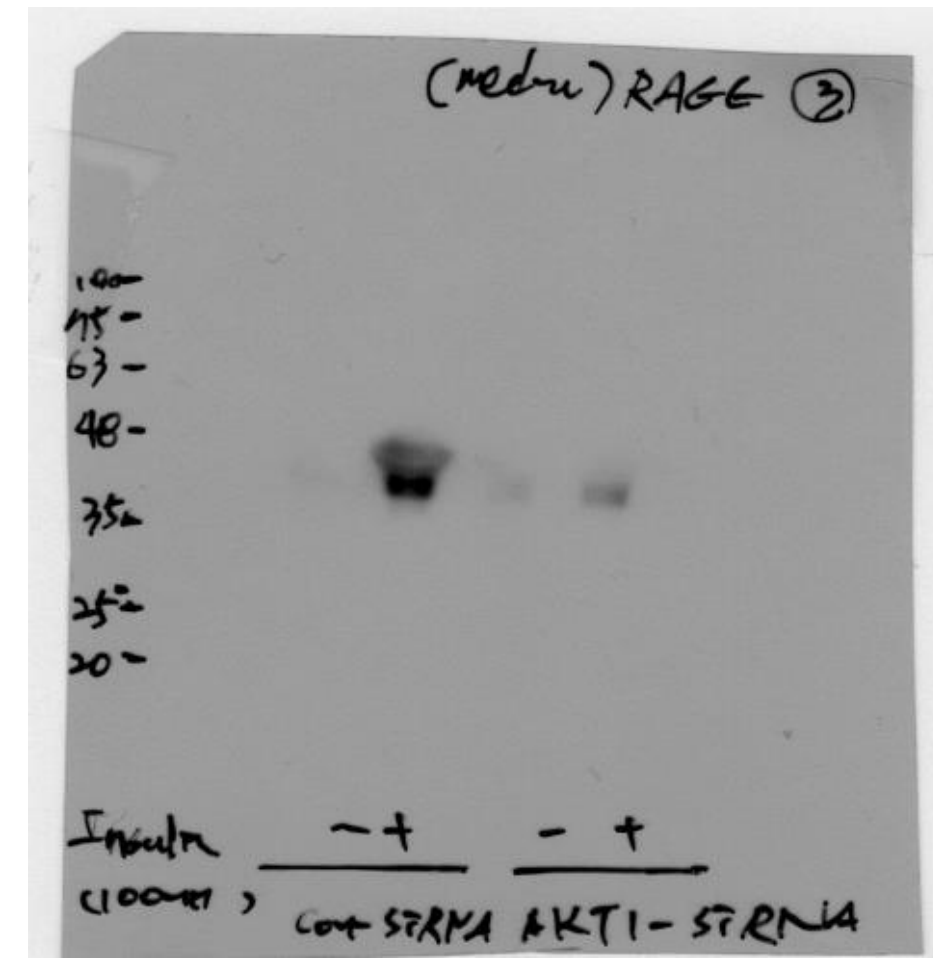

Figure 4B lower panel

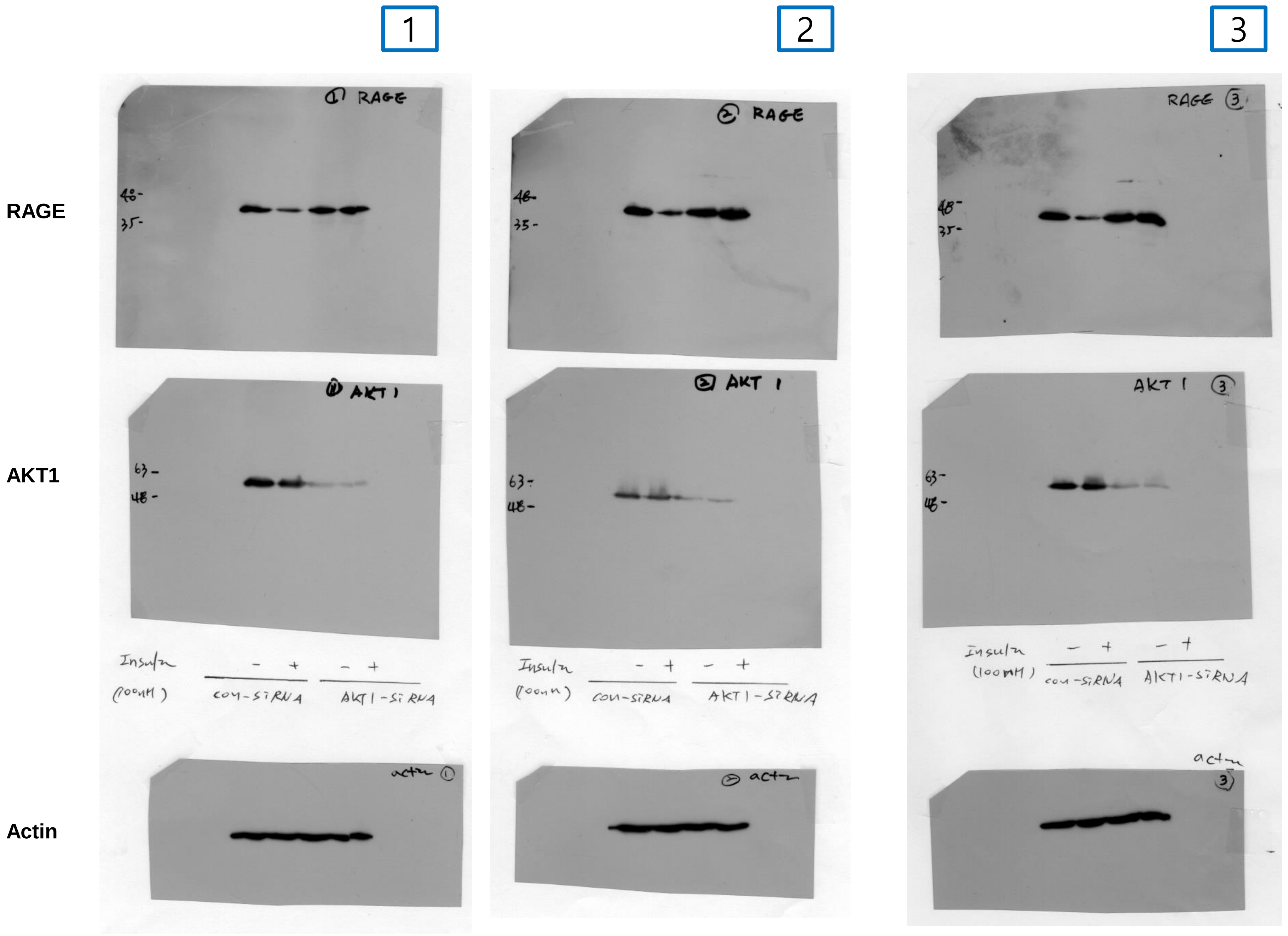

Figure 4C

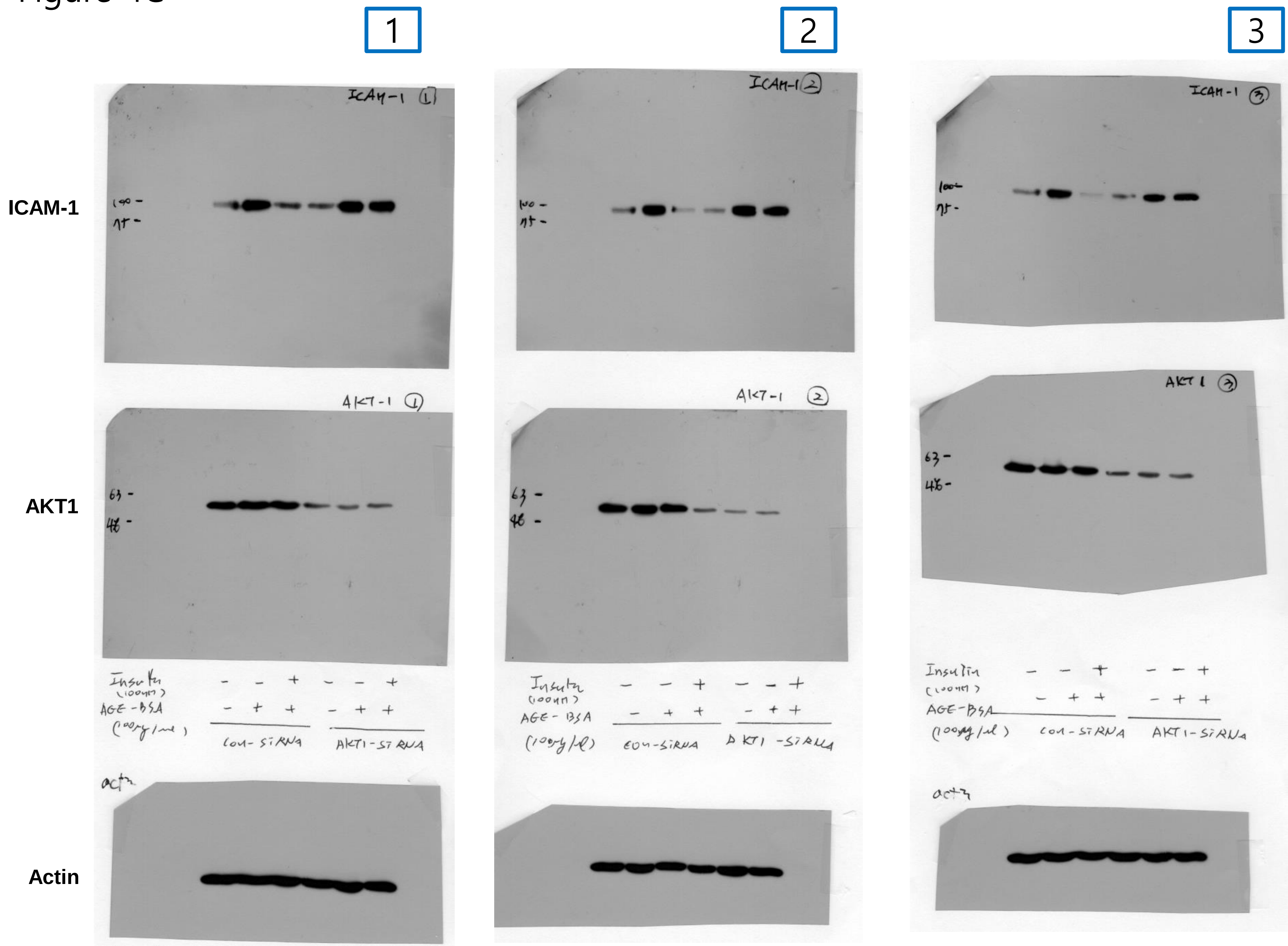

Figure 4C

4

ICAM-1

AKT1

Actin

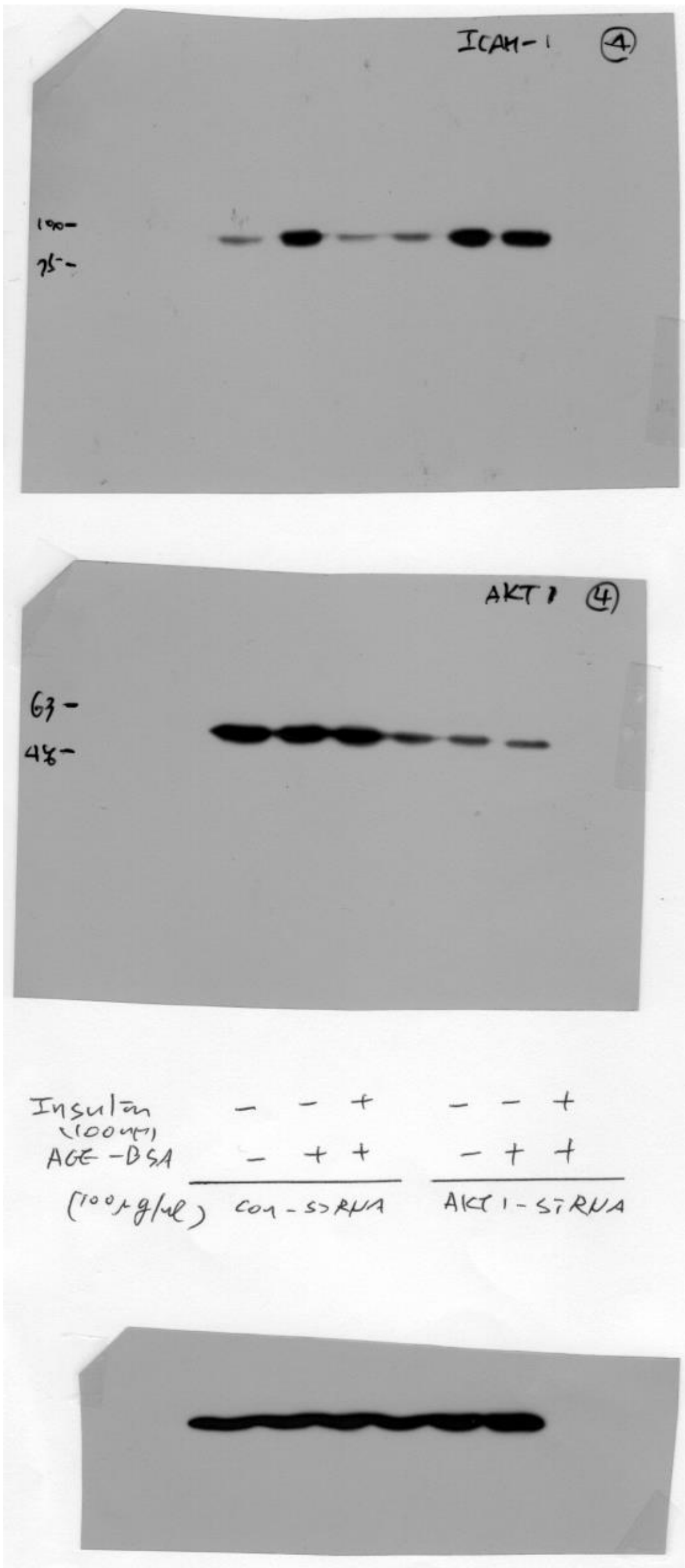

Figure 5A upper panel

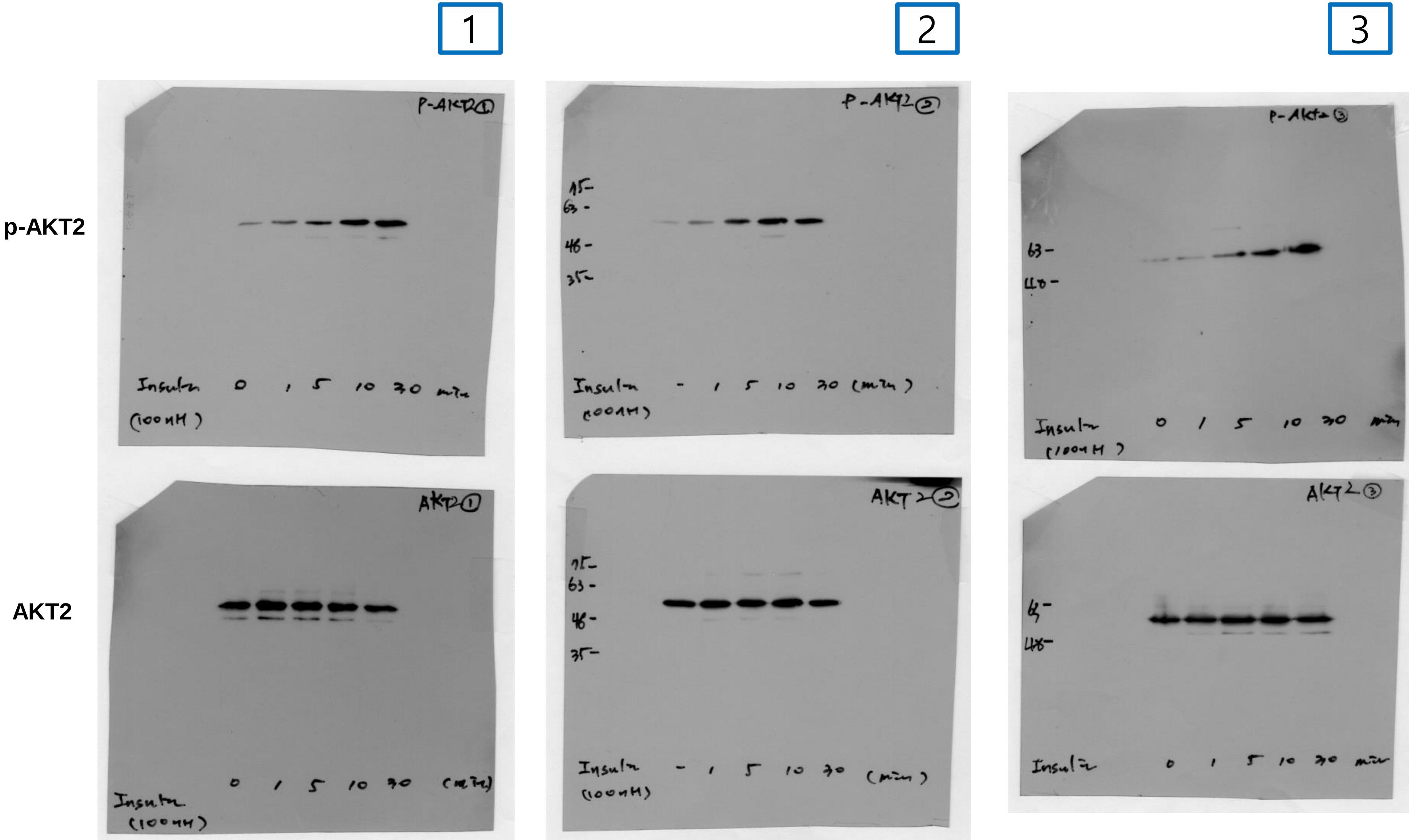

# Figure 5A lower panel

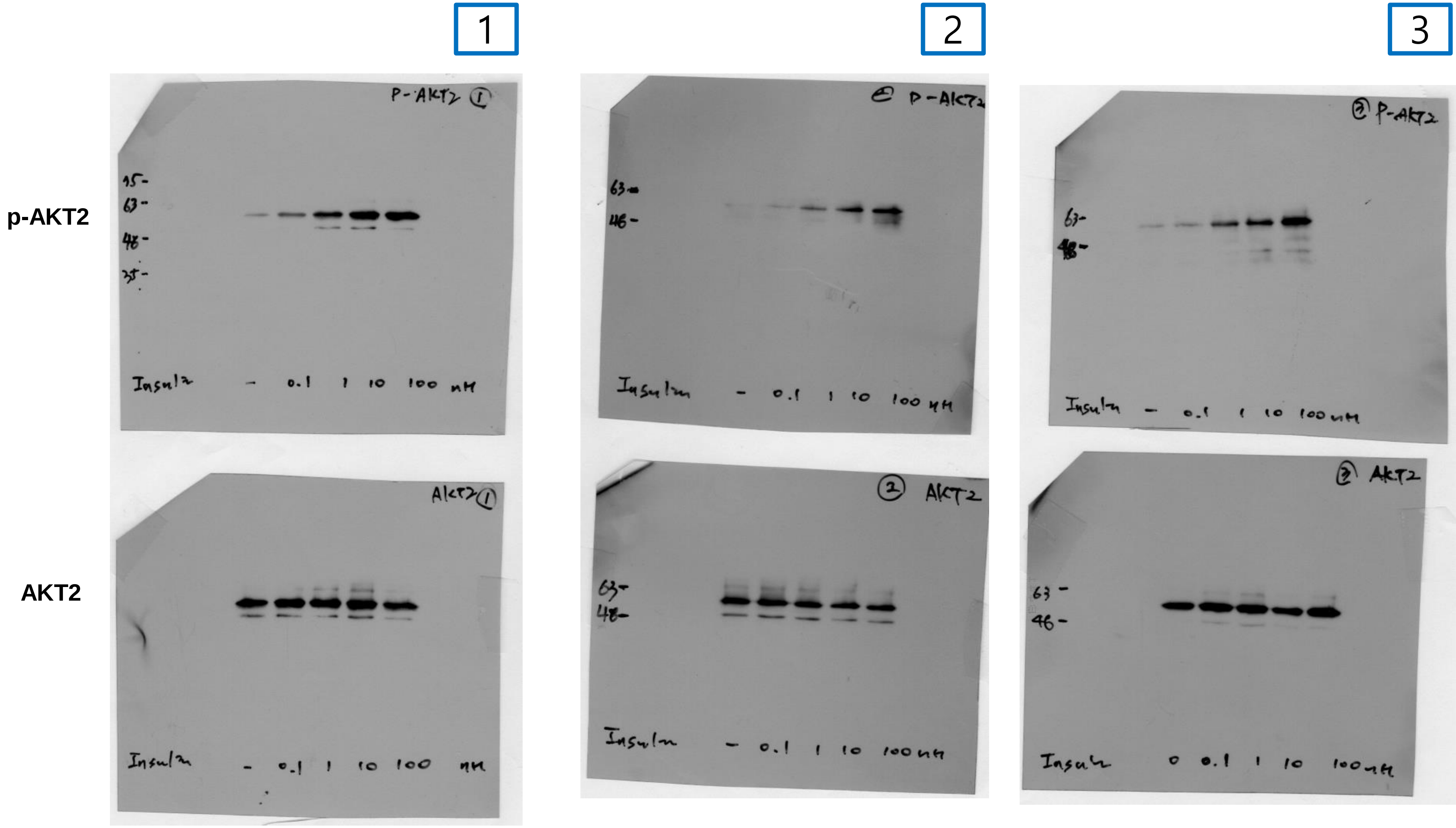

Figure 5B upper panel

1 2 3

RAGE

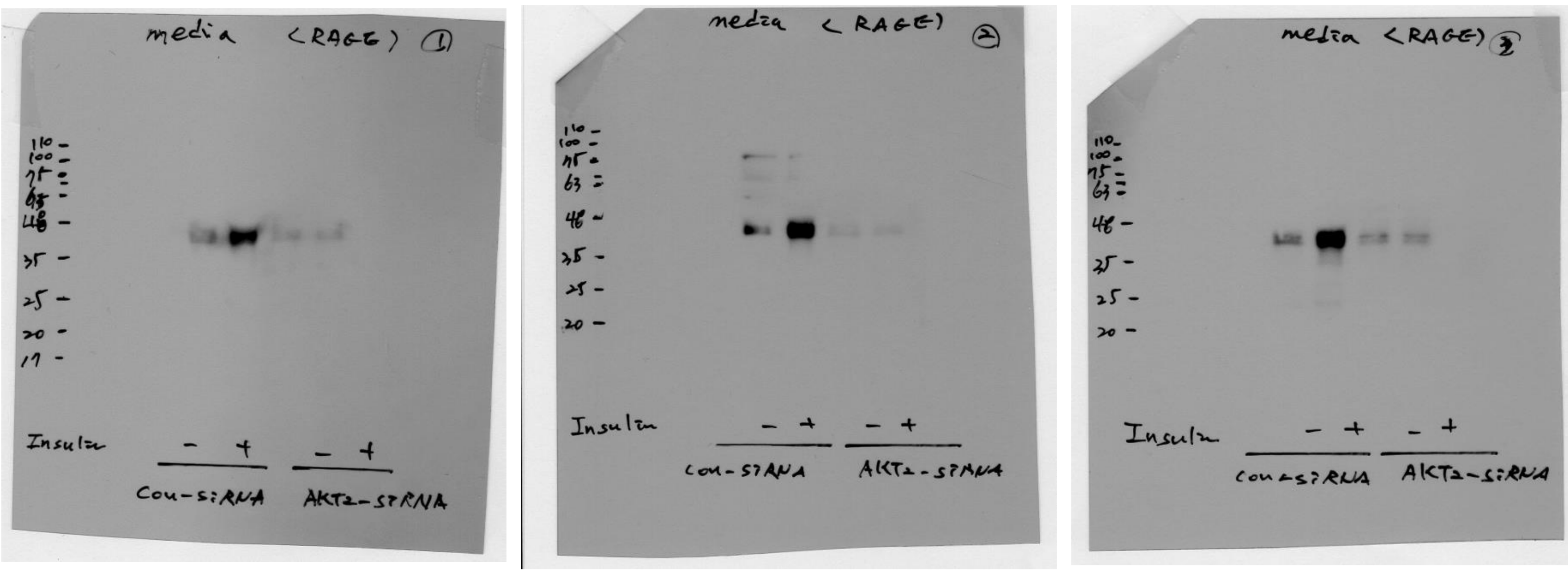

# Figure 5B lower panel

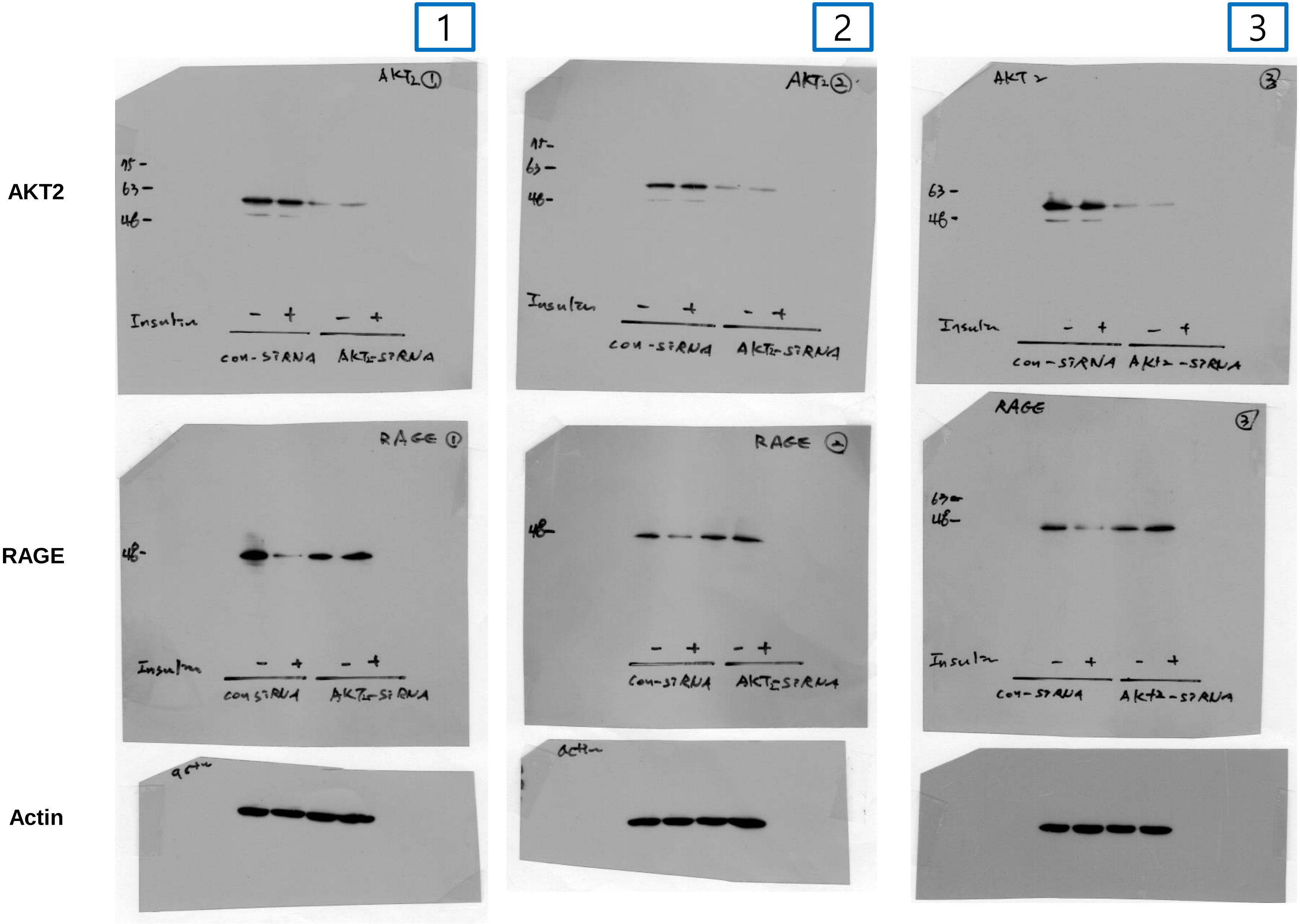

Figure 5C

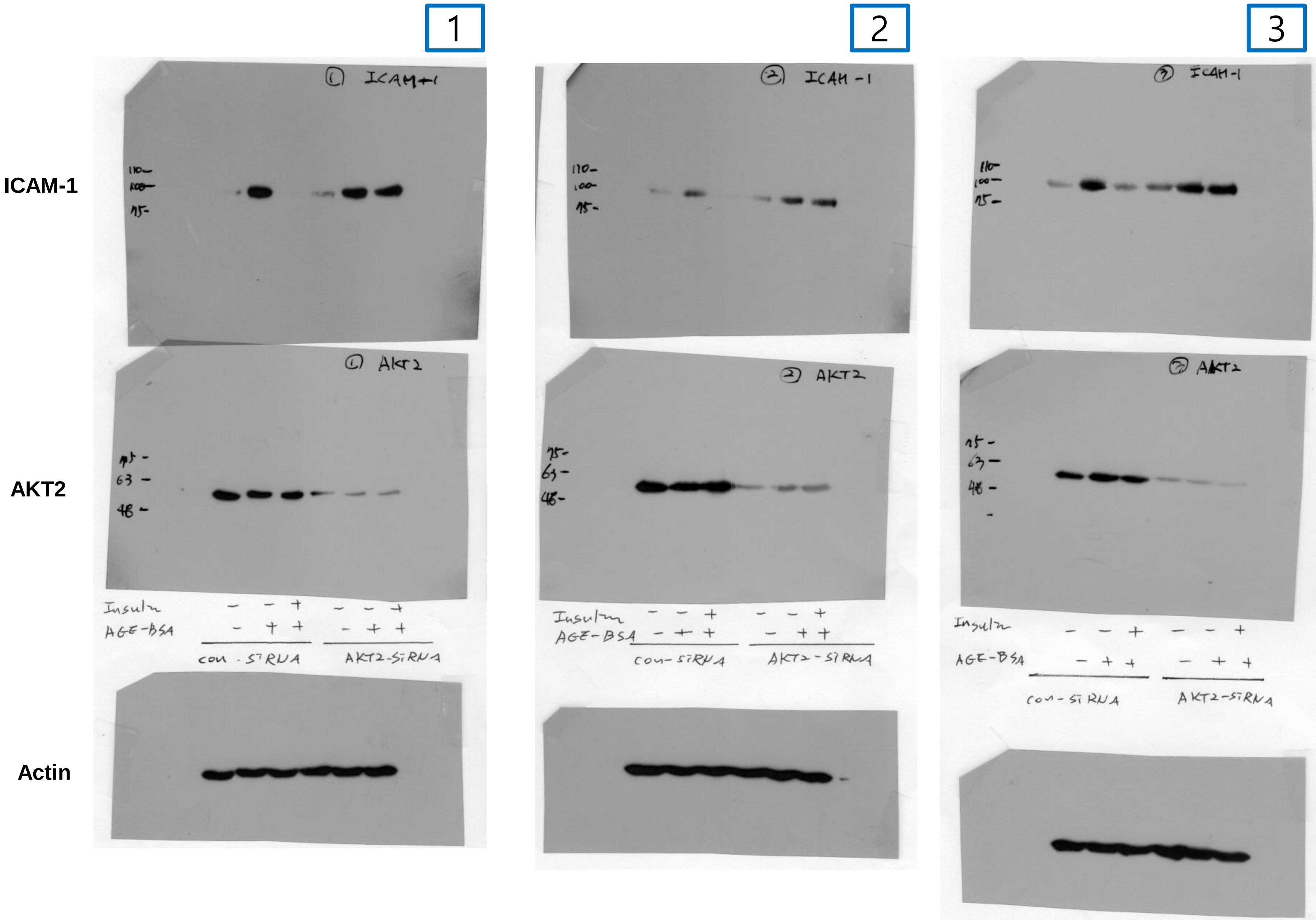

Figure 5C

4

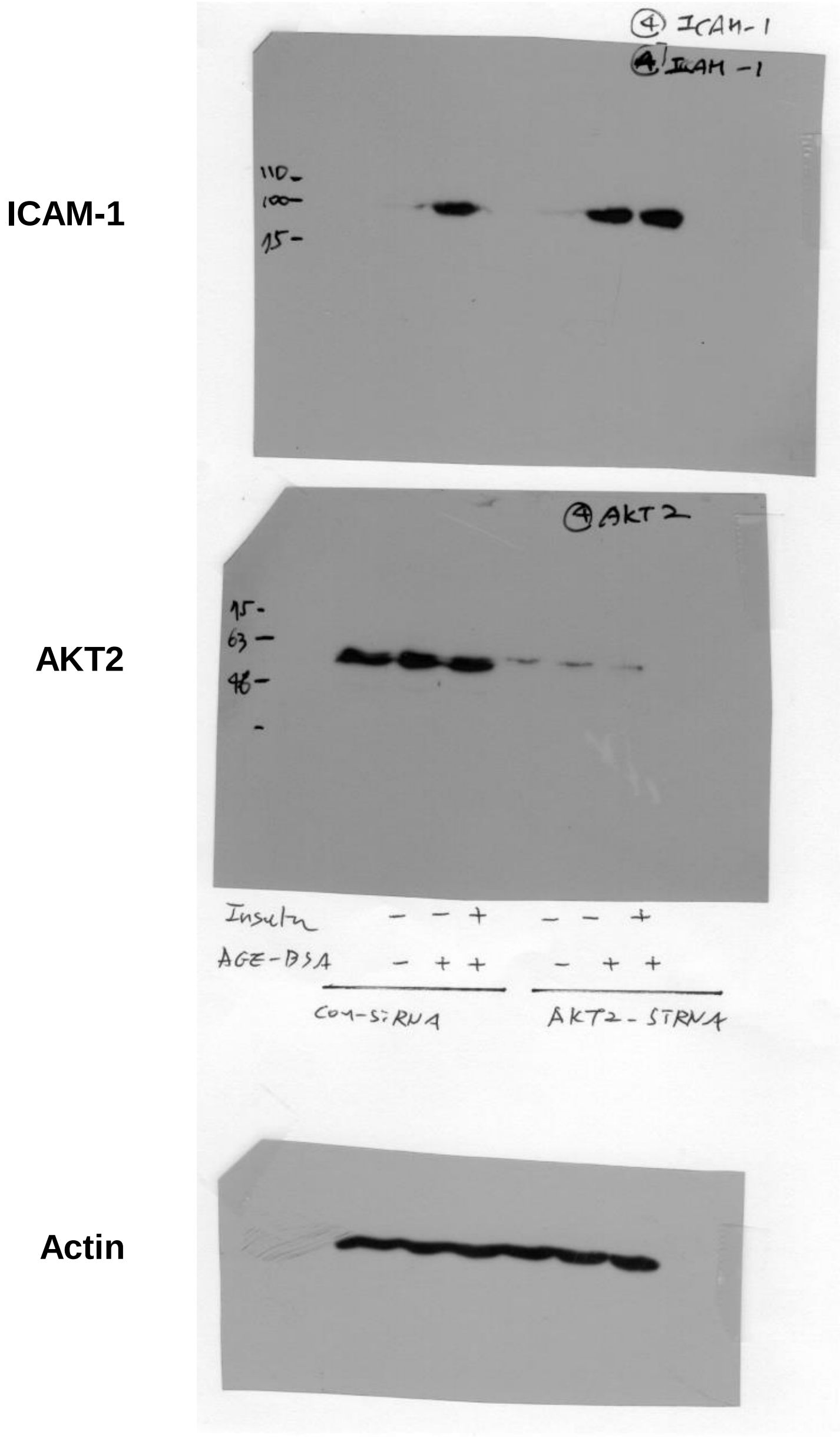

Figure 6A upper panel

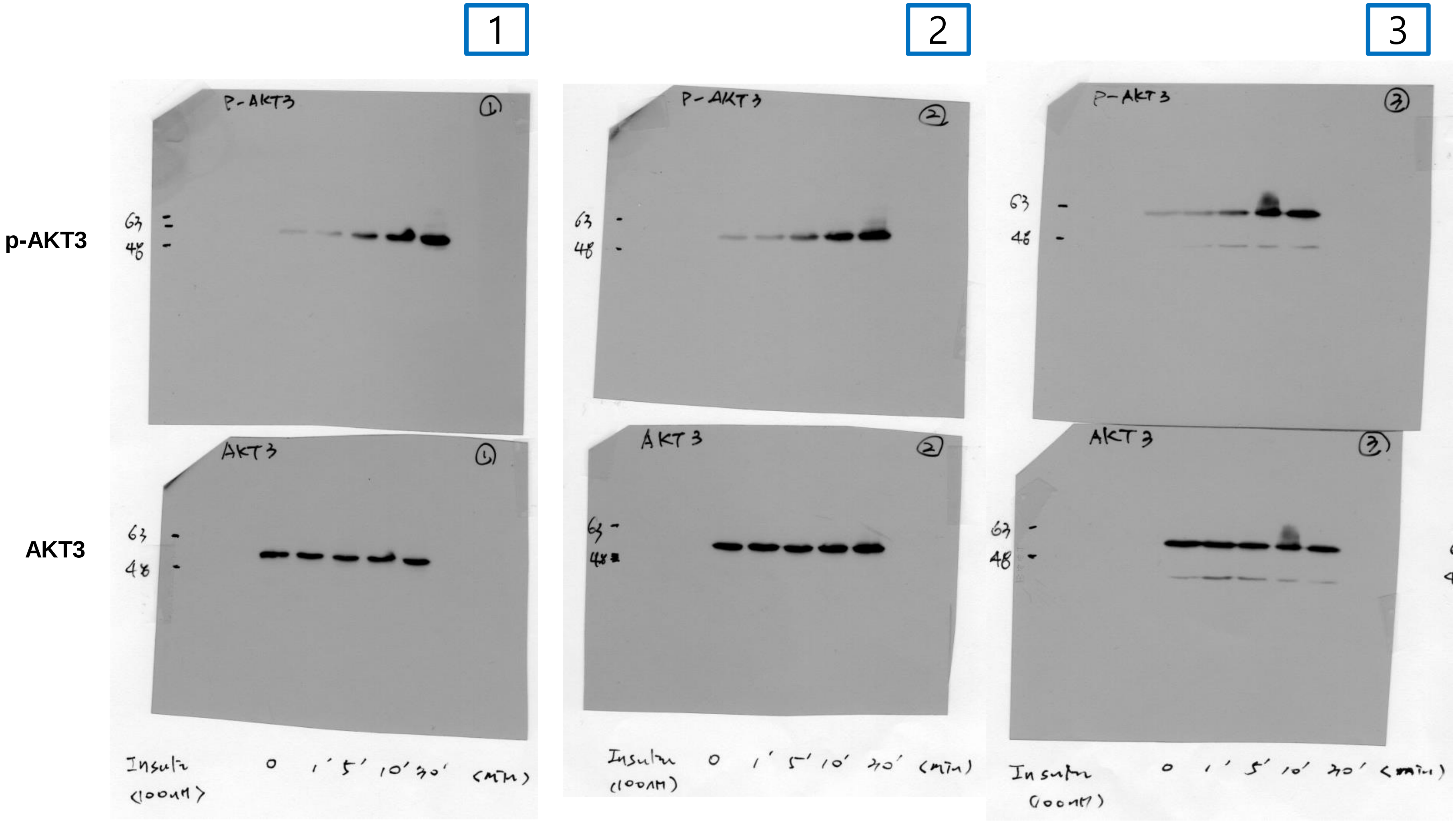

Figure 6A lower panel

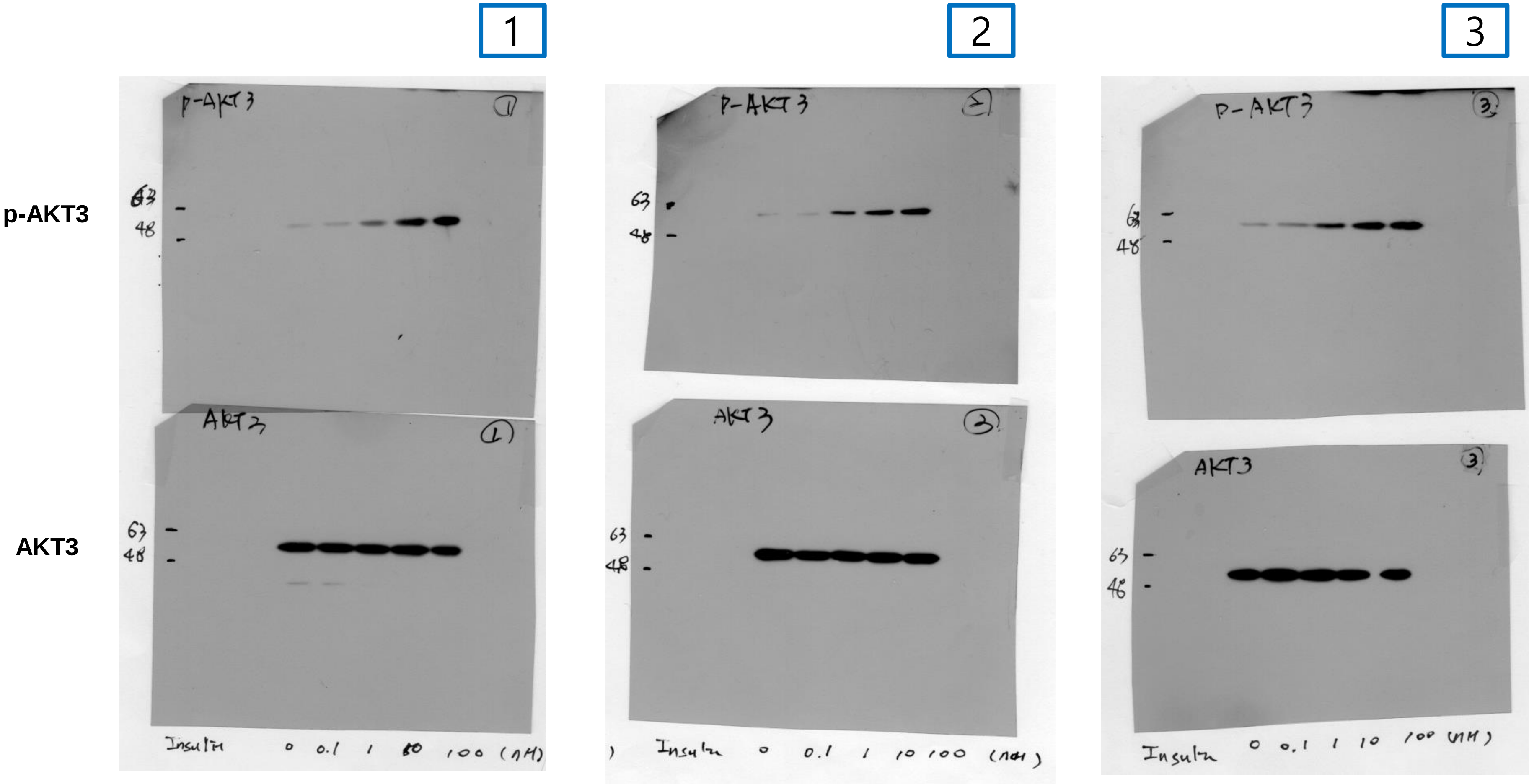

# Figure 6B upper panel

1 2 3

RAGE

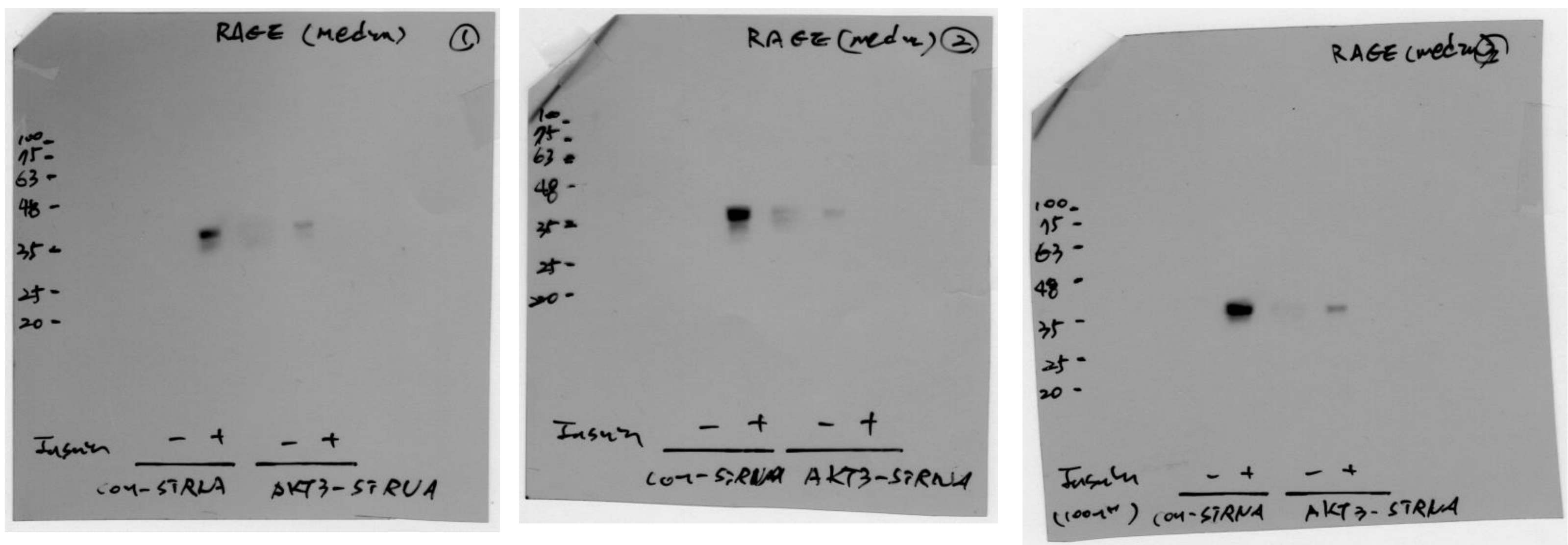

Figure 6B lower panel

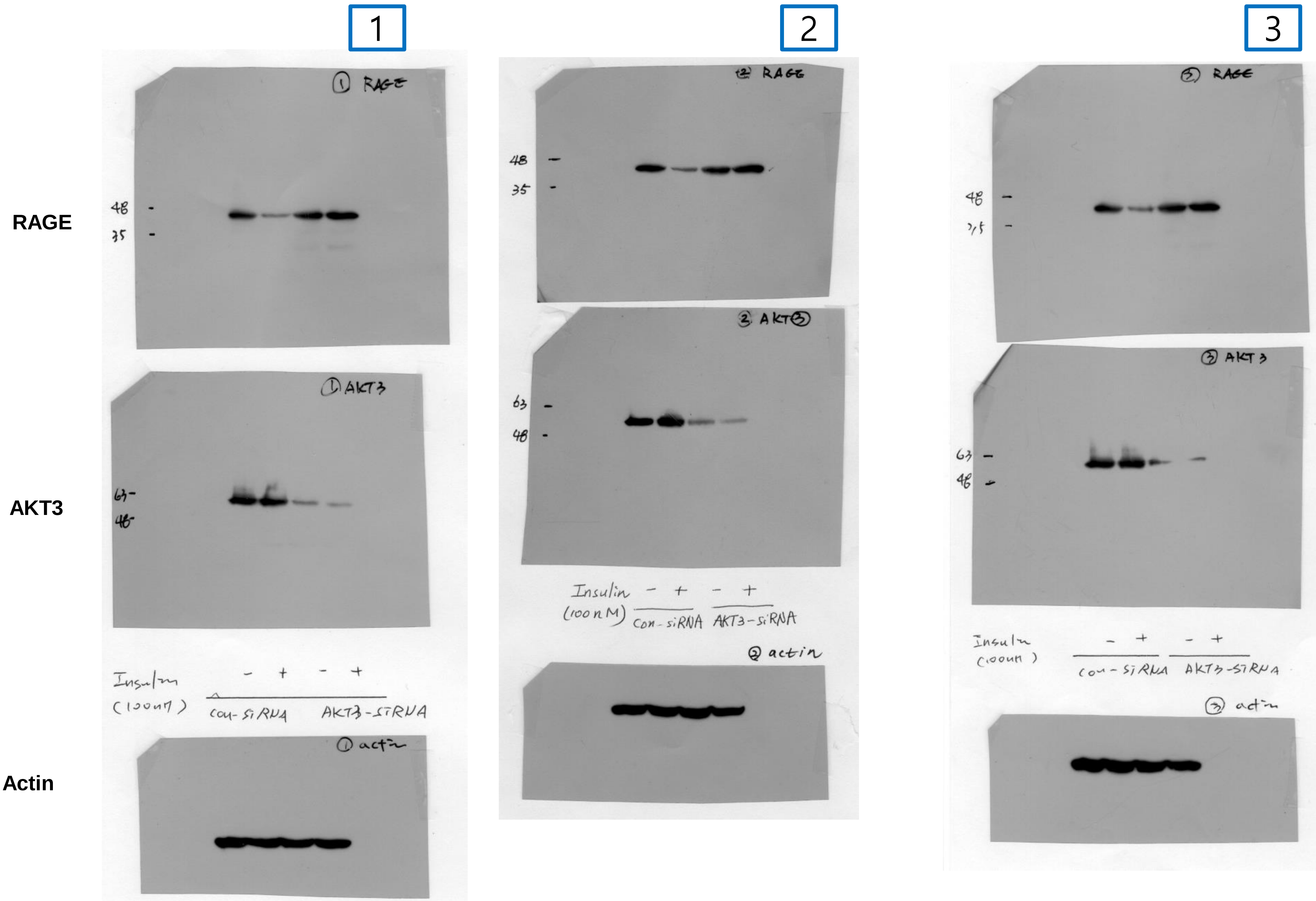

# Figure 6C

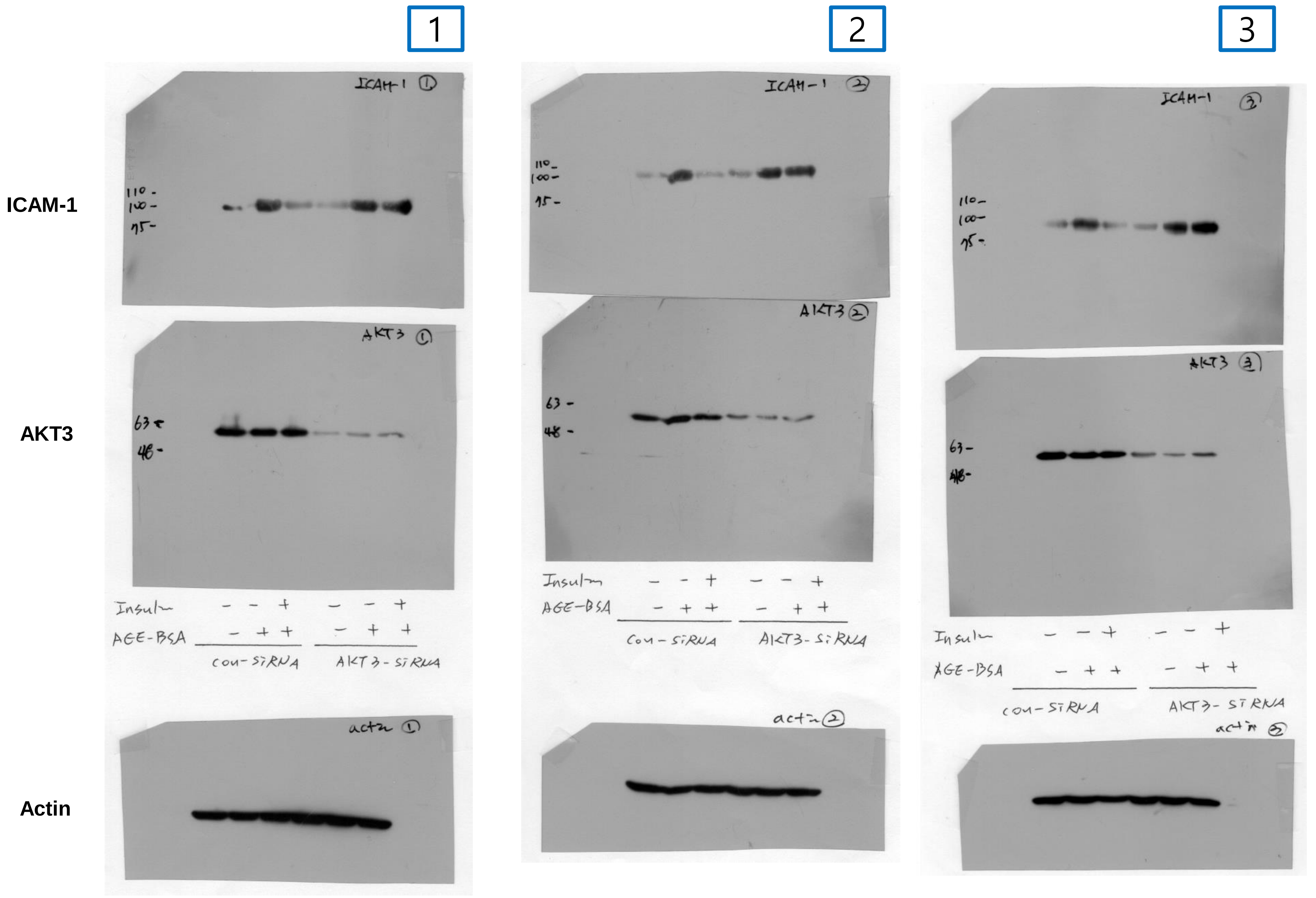

Figure 6C

4

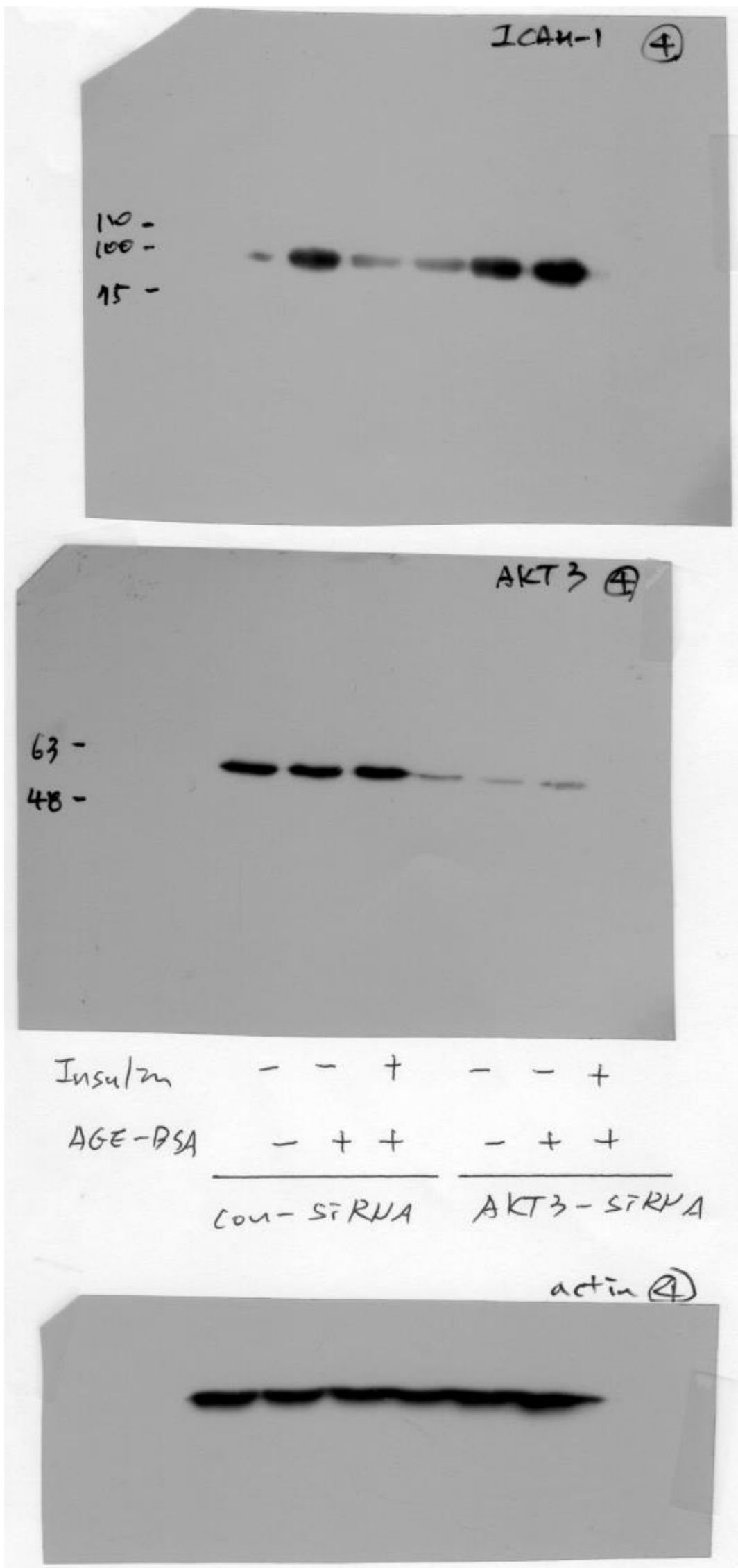


ICAM-1

AKT3

Actin

Figure 7A (b)

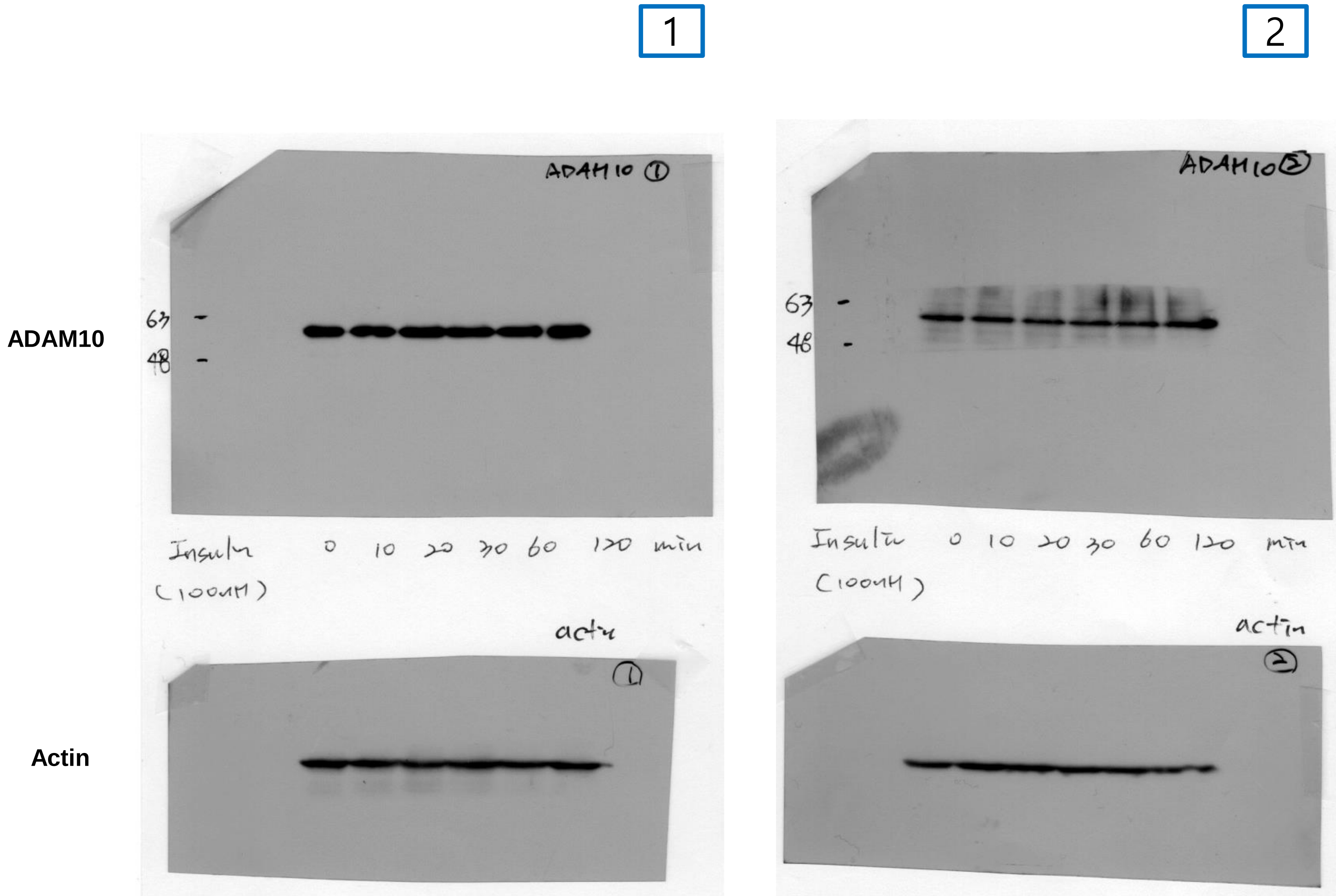
1
2
ADAM10
Actin
ADAM10 ①
ADAM10 ②
63
48
Insulin
(100nM)
0
10
20
30
60
120
min
actin ①
actin ②

Figure 7A (b)

3

ADAM10

Actin

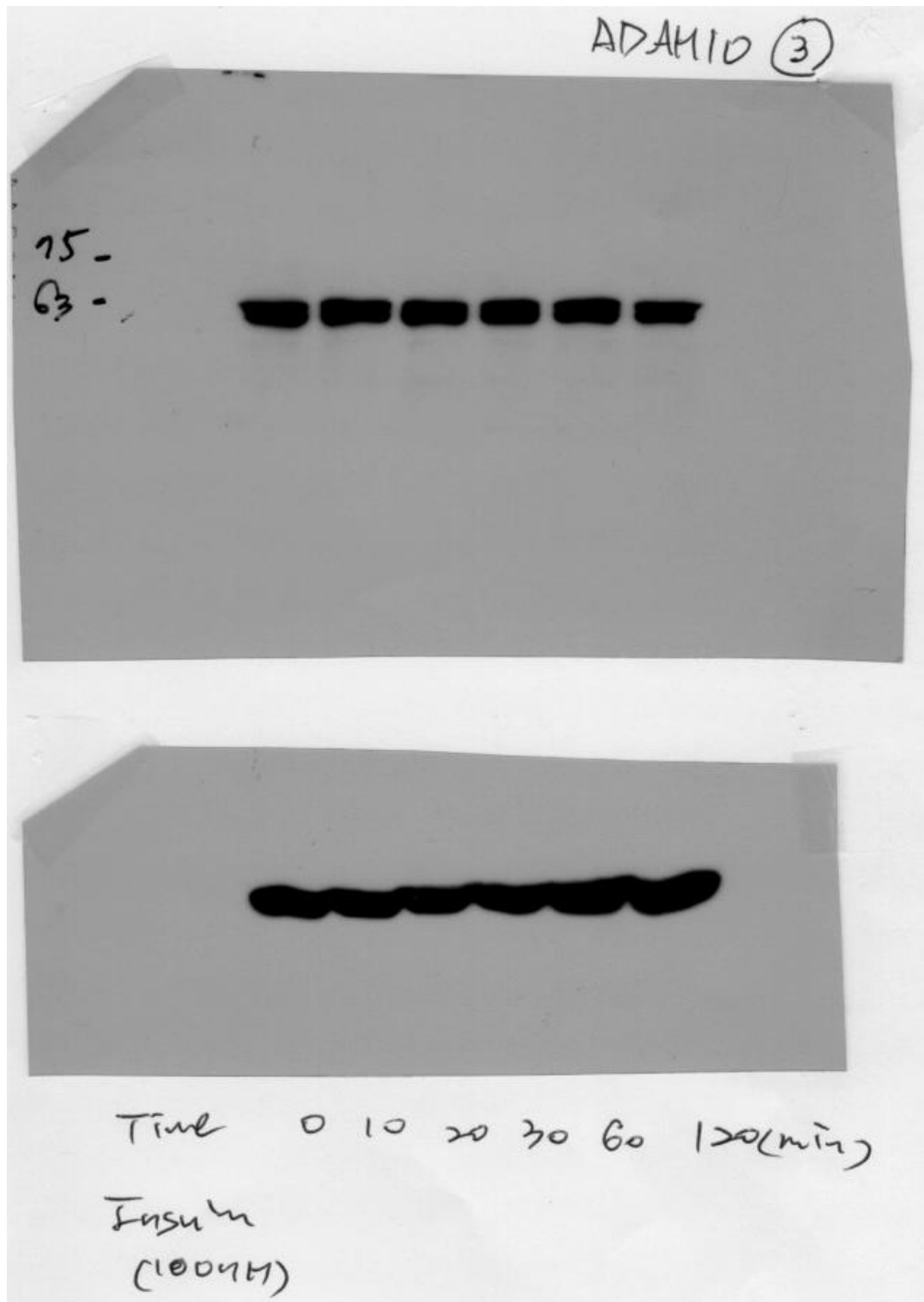

Figure 7B upper panel

RAGE

1

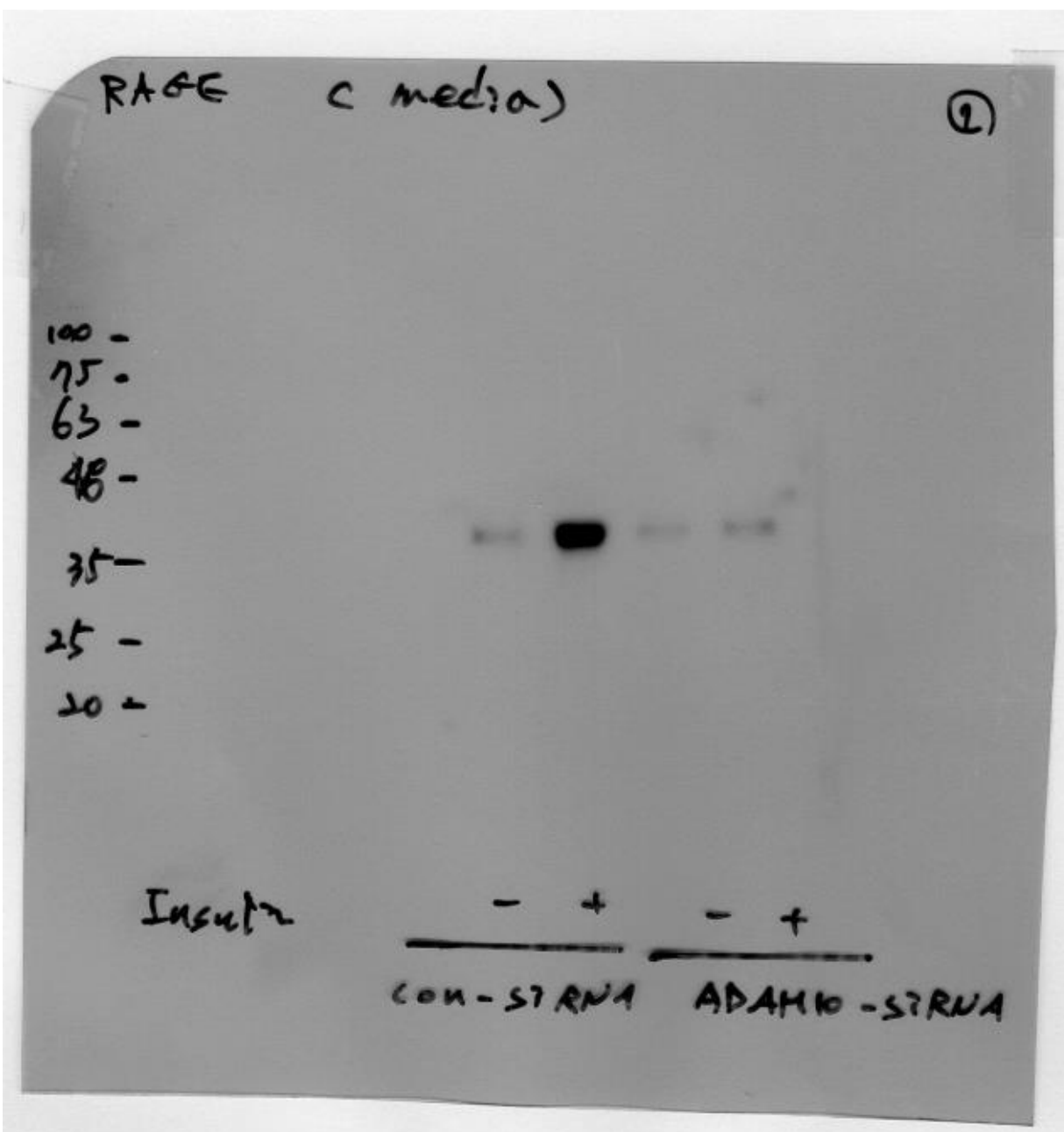


2

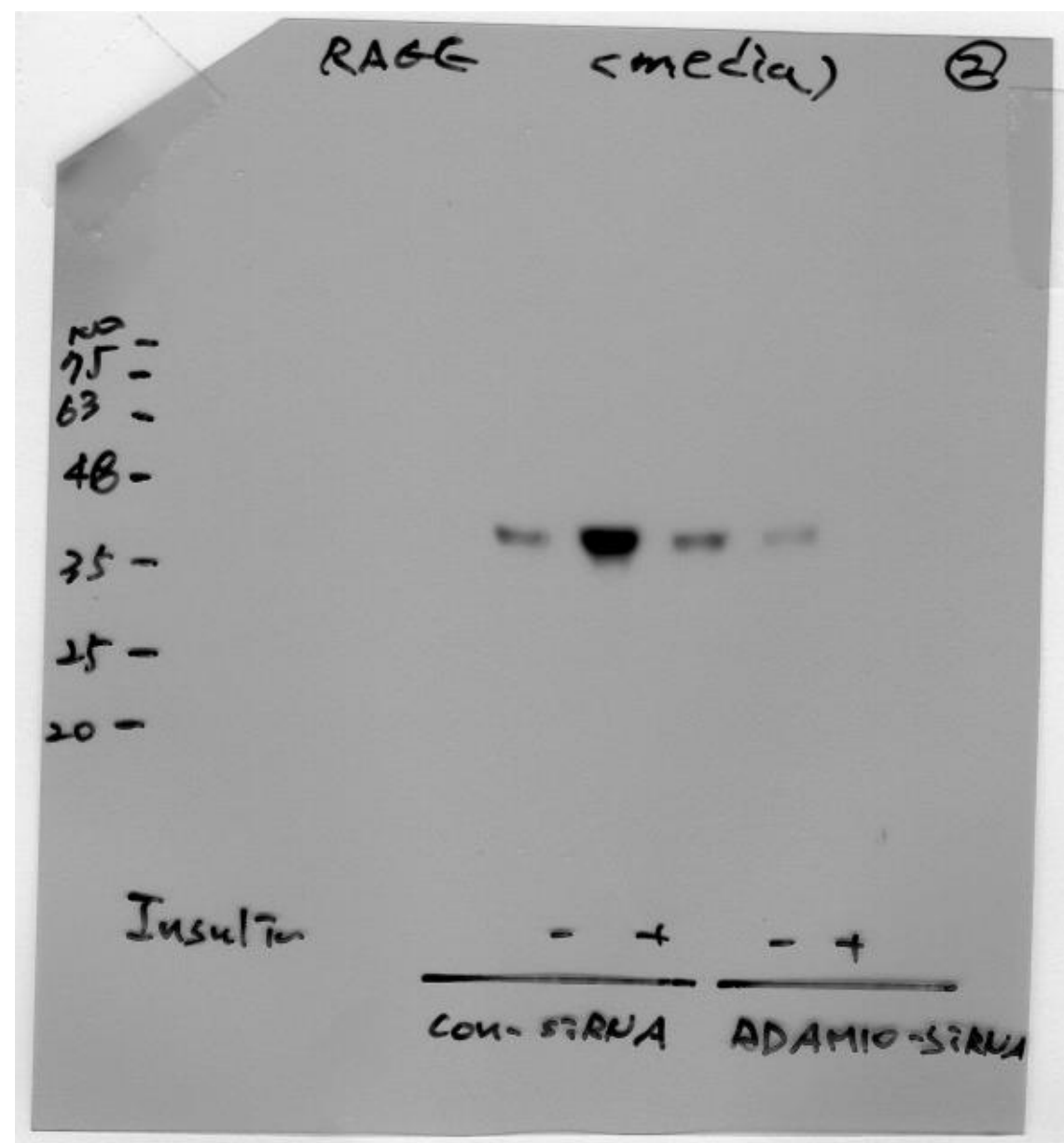


3

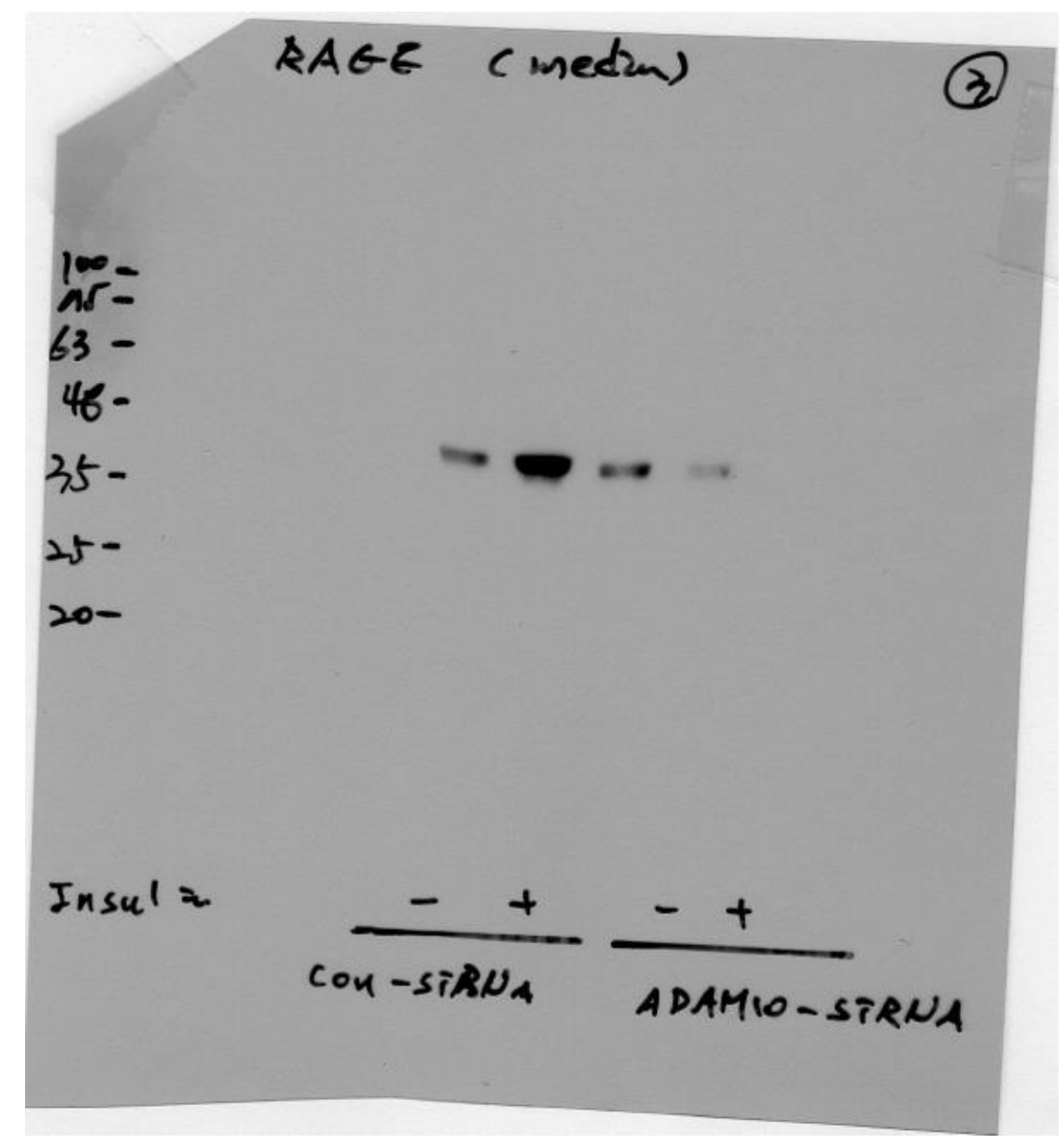

# Figure 7B lower panel

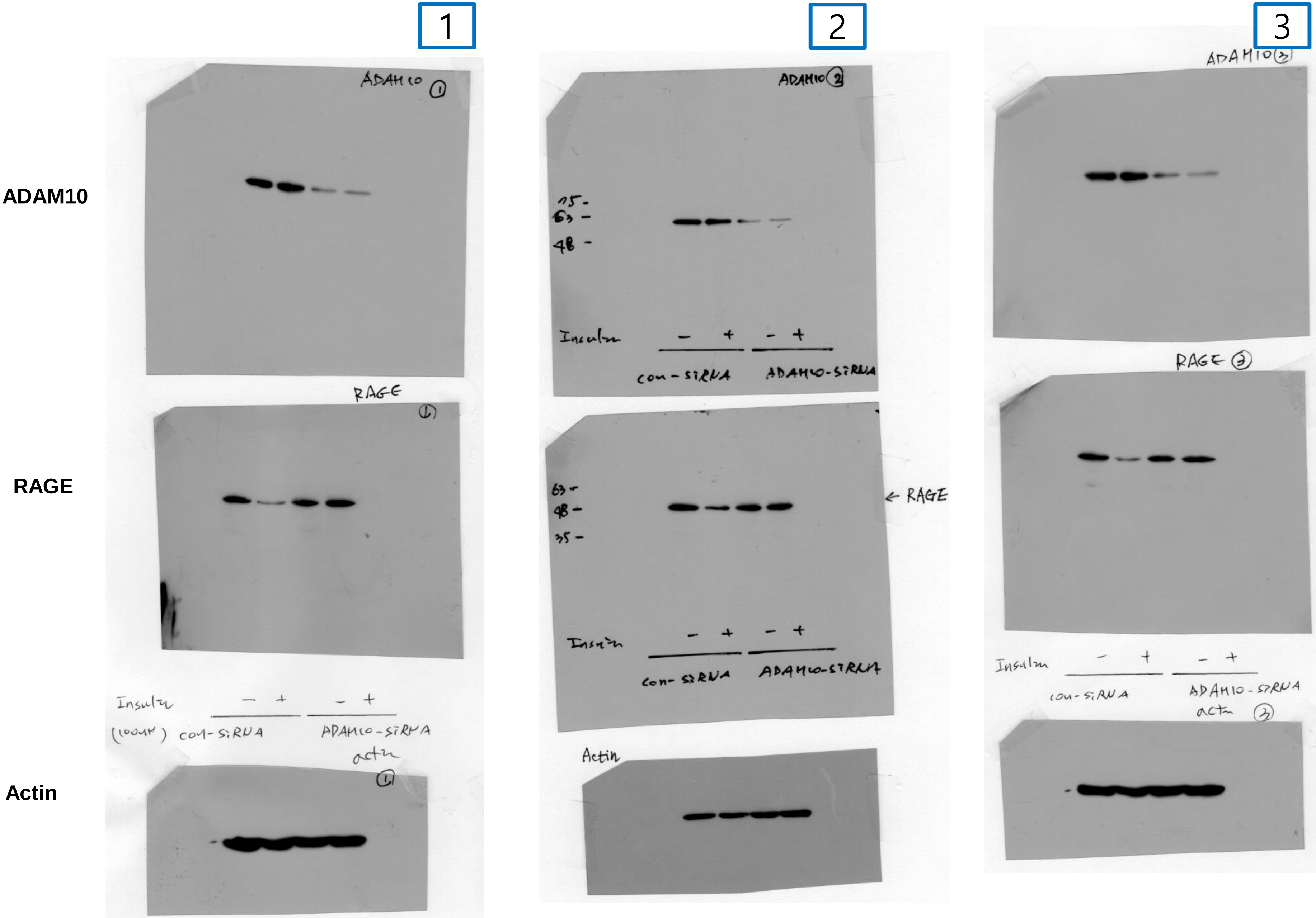

# Figure 7C

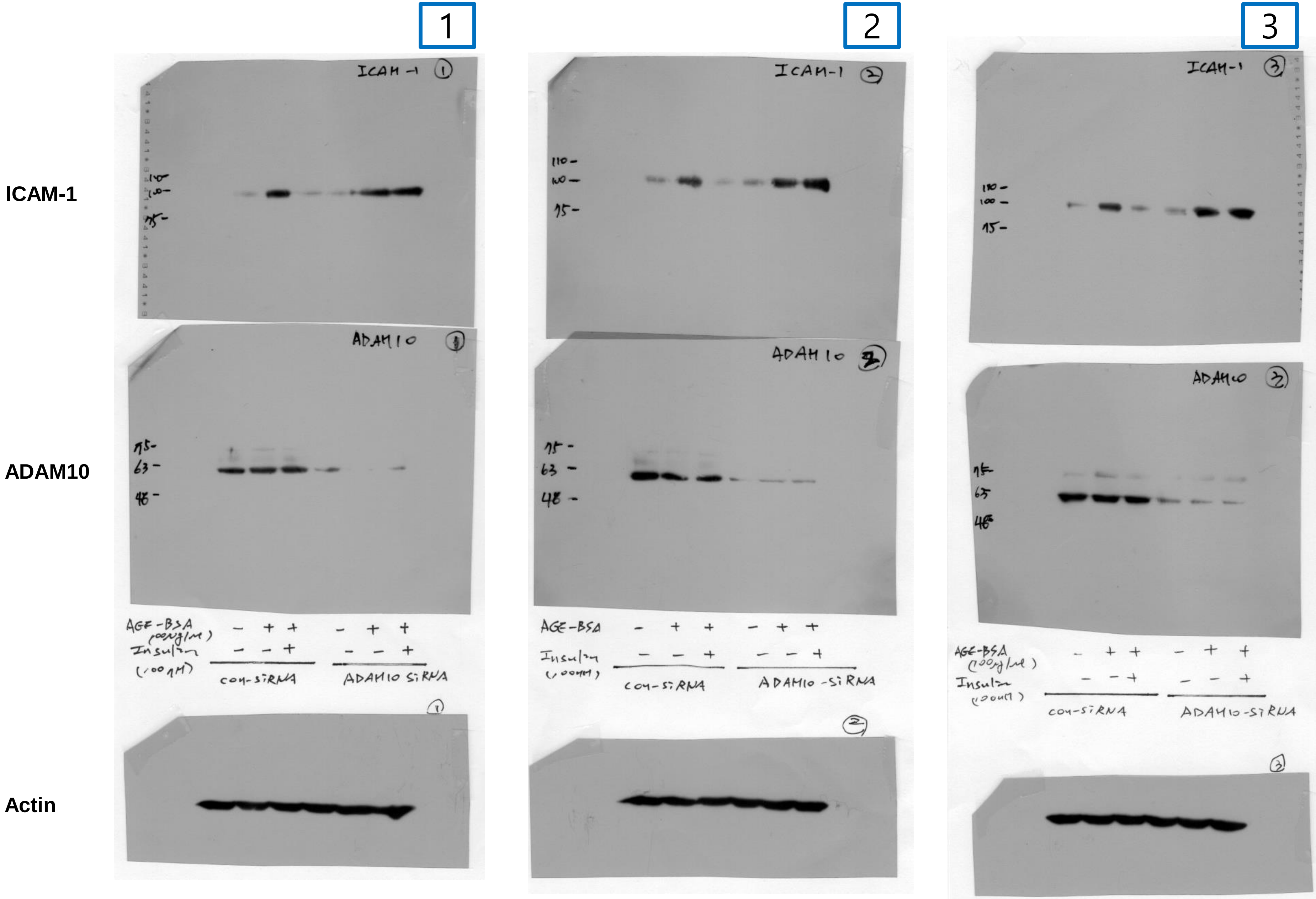

Figure 7C

ICAM-1

ADAM10

Actin

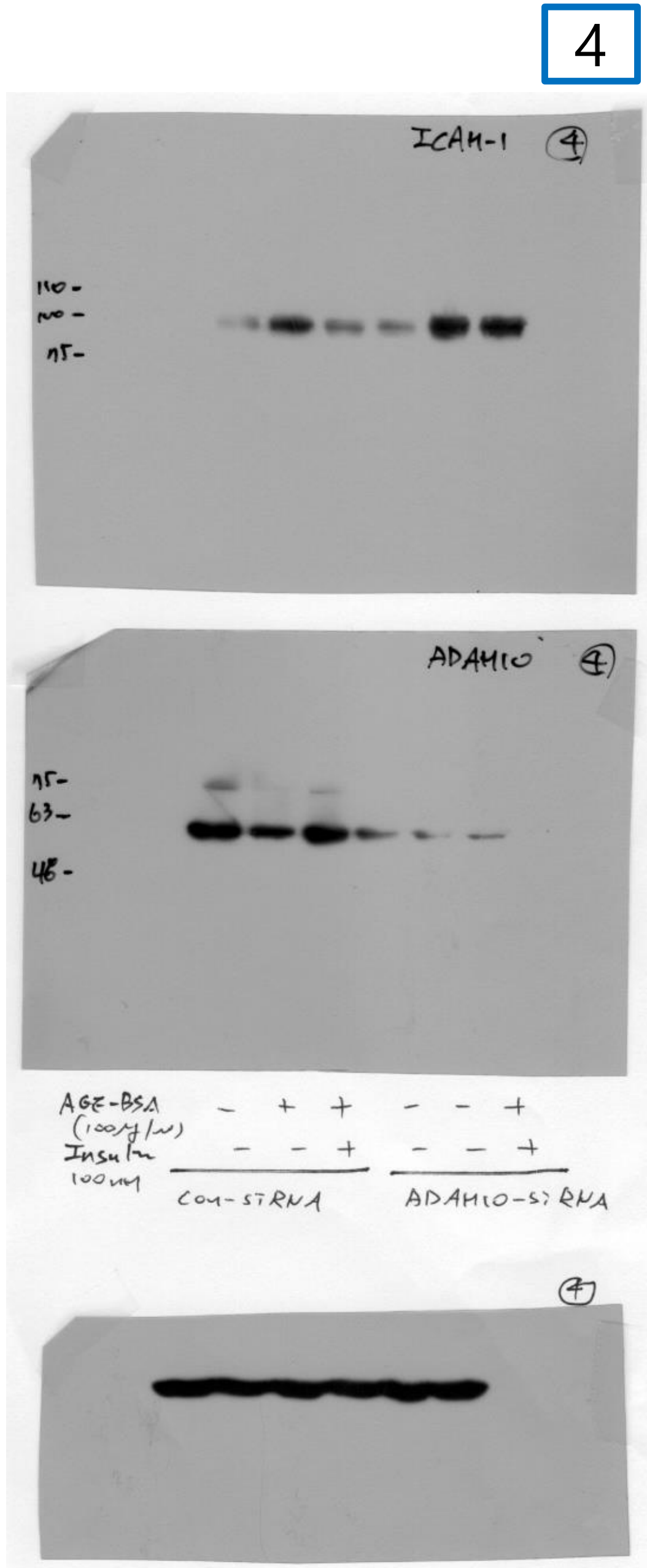

Figure 8B(b)

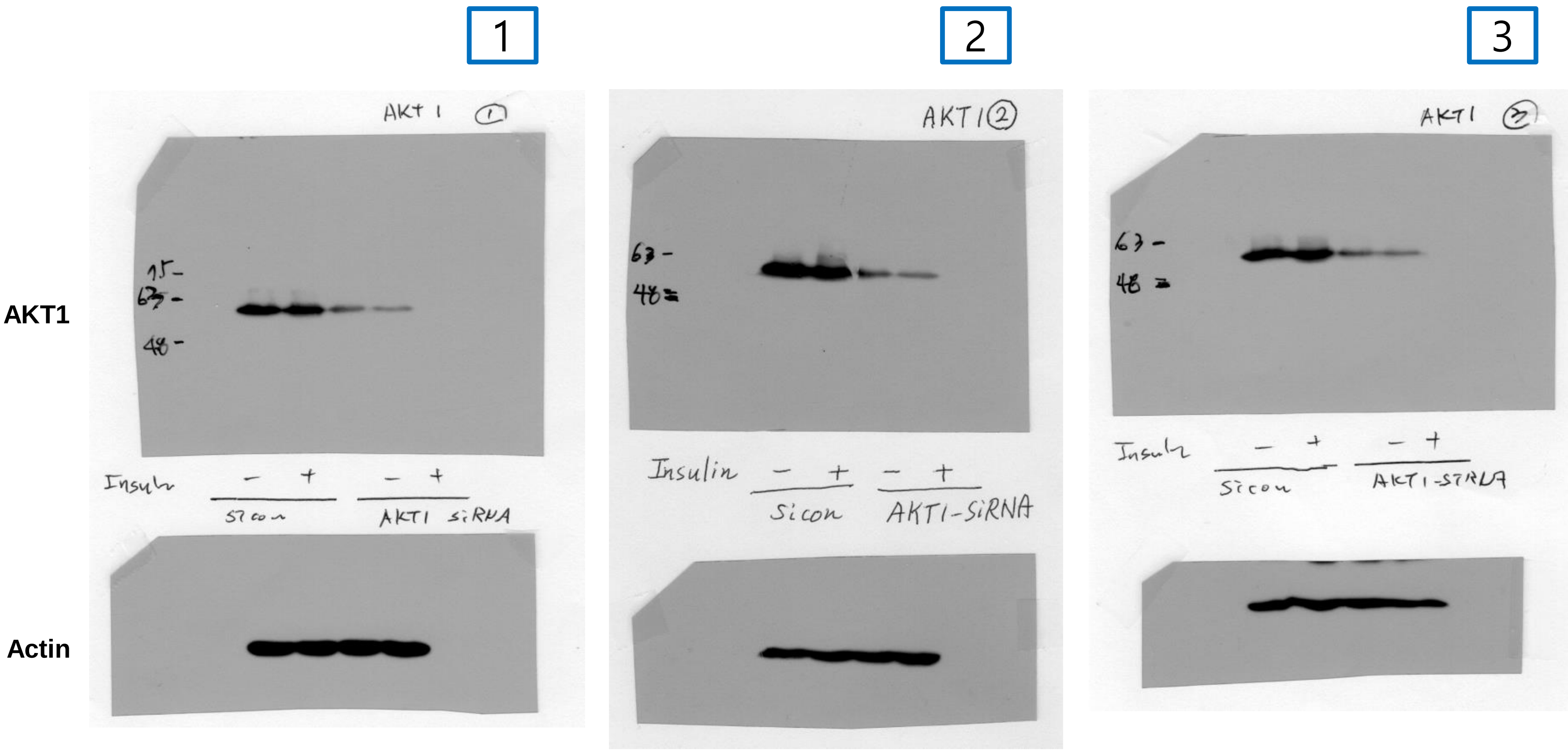

Figure 8C(b)

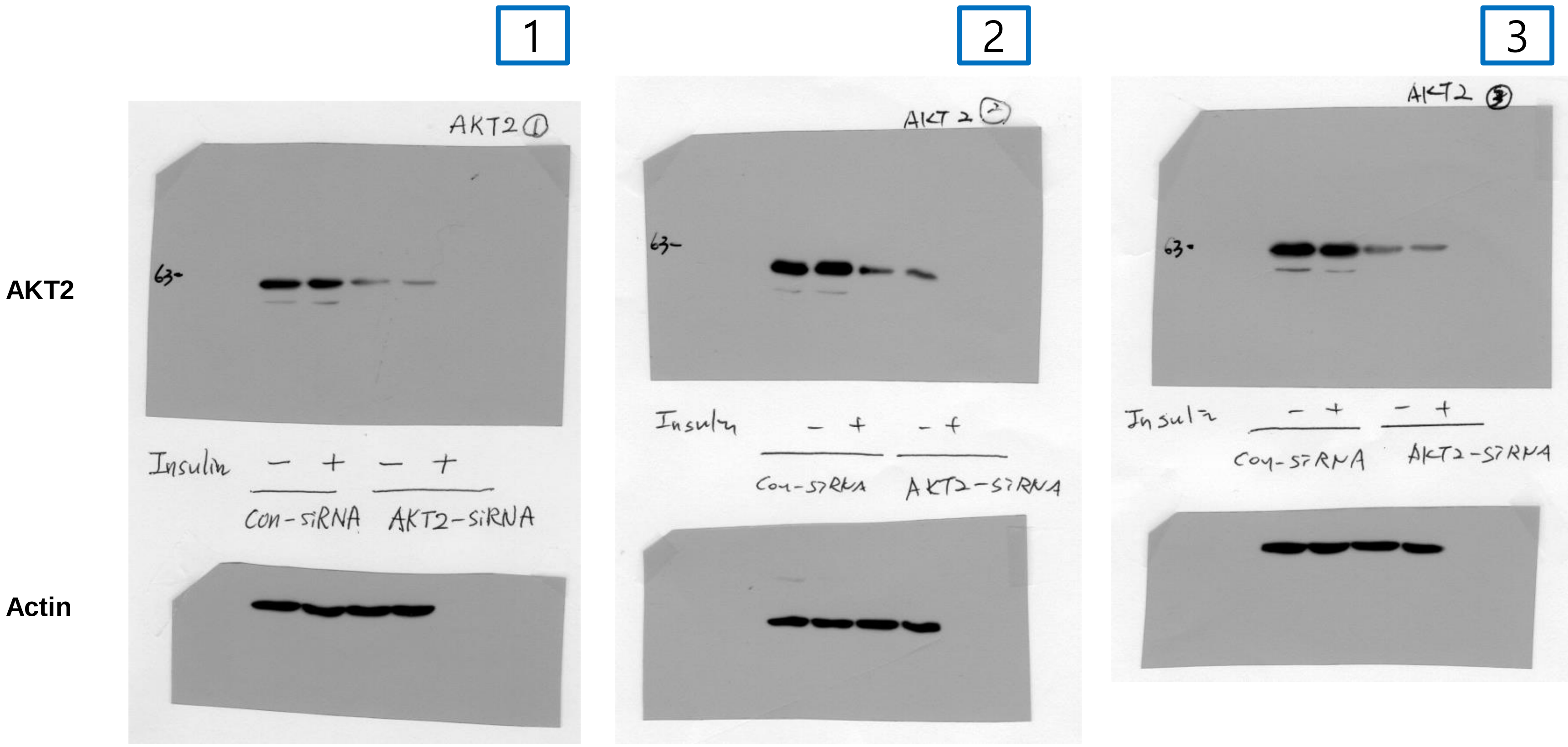
1
2
3
AKT2
Actin
AKT2 ①
63-
Insulin − + − +
Con-siRNA
AKT2-siRNA
AKT2 ②
AKT2 ③

Figure 8D(b)

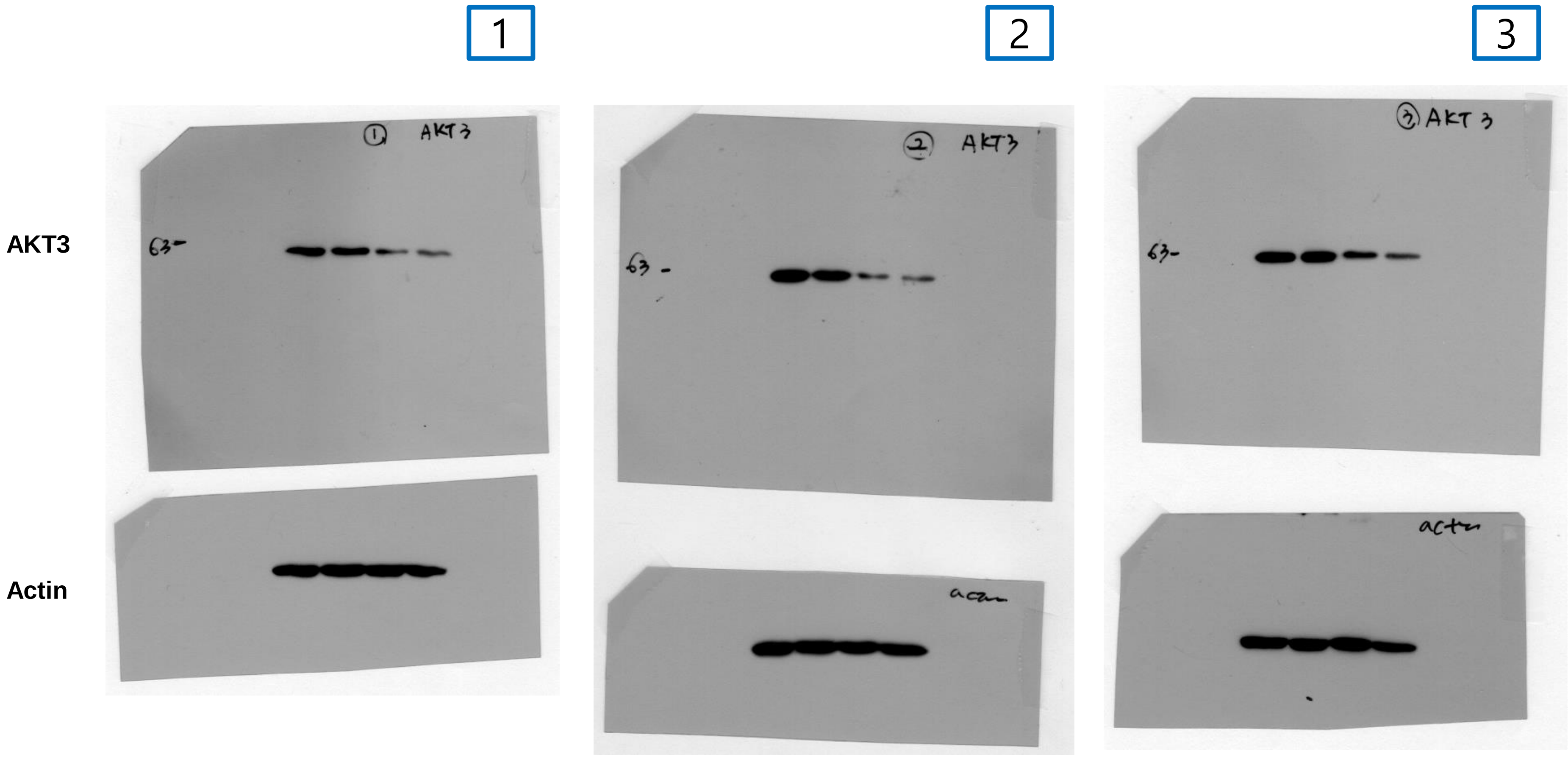

Figure 9A: Lt panel - Input sample

**ADAM10**

**Rab14**

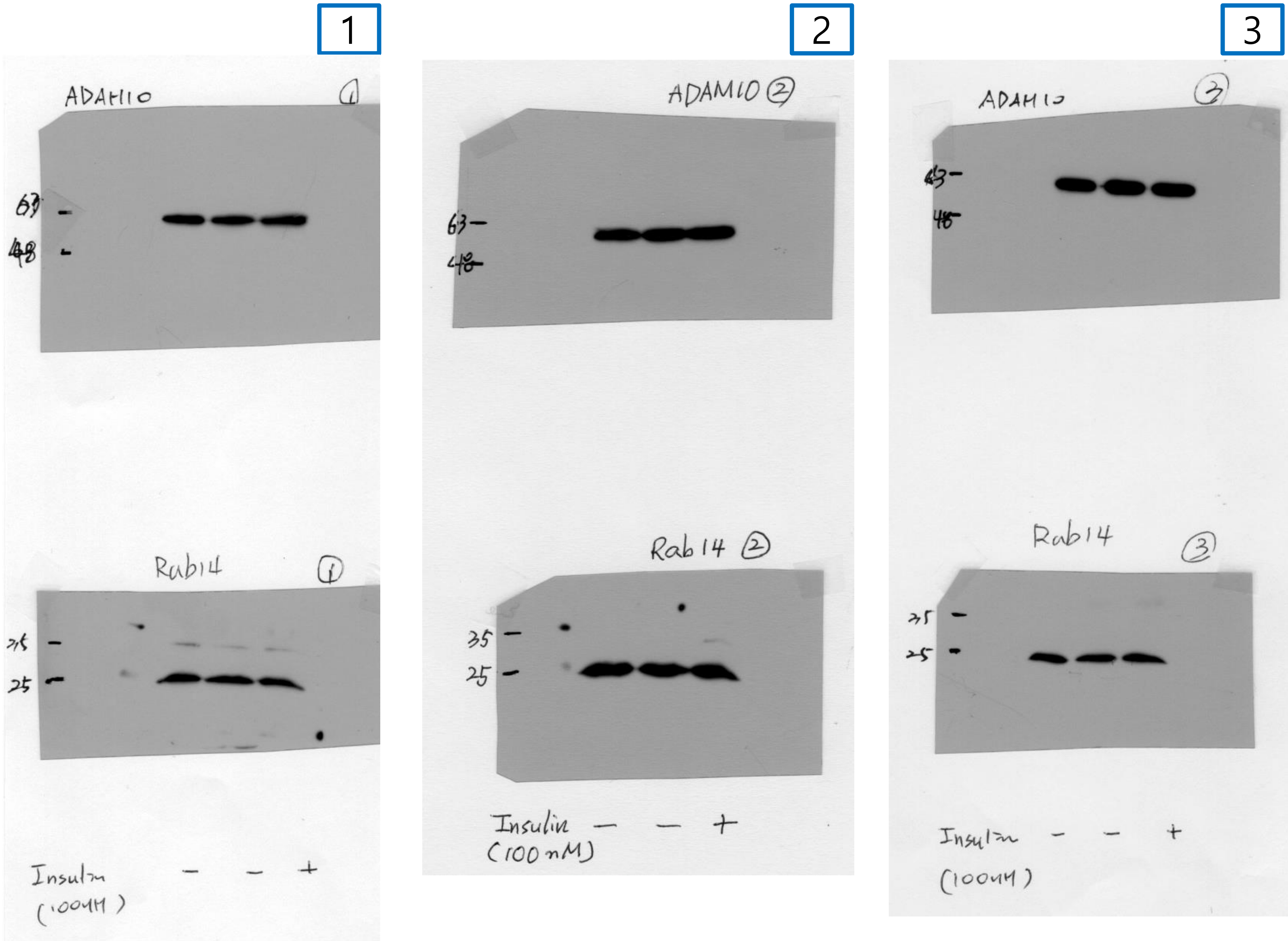

# Figure 9A: Lt panel - Input sample

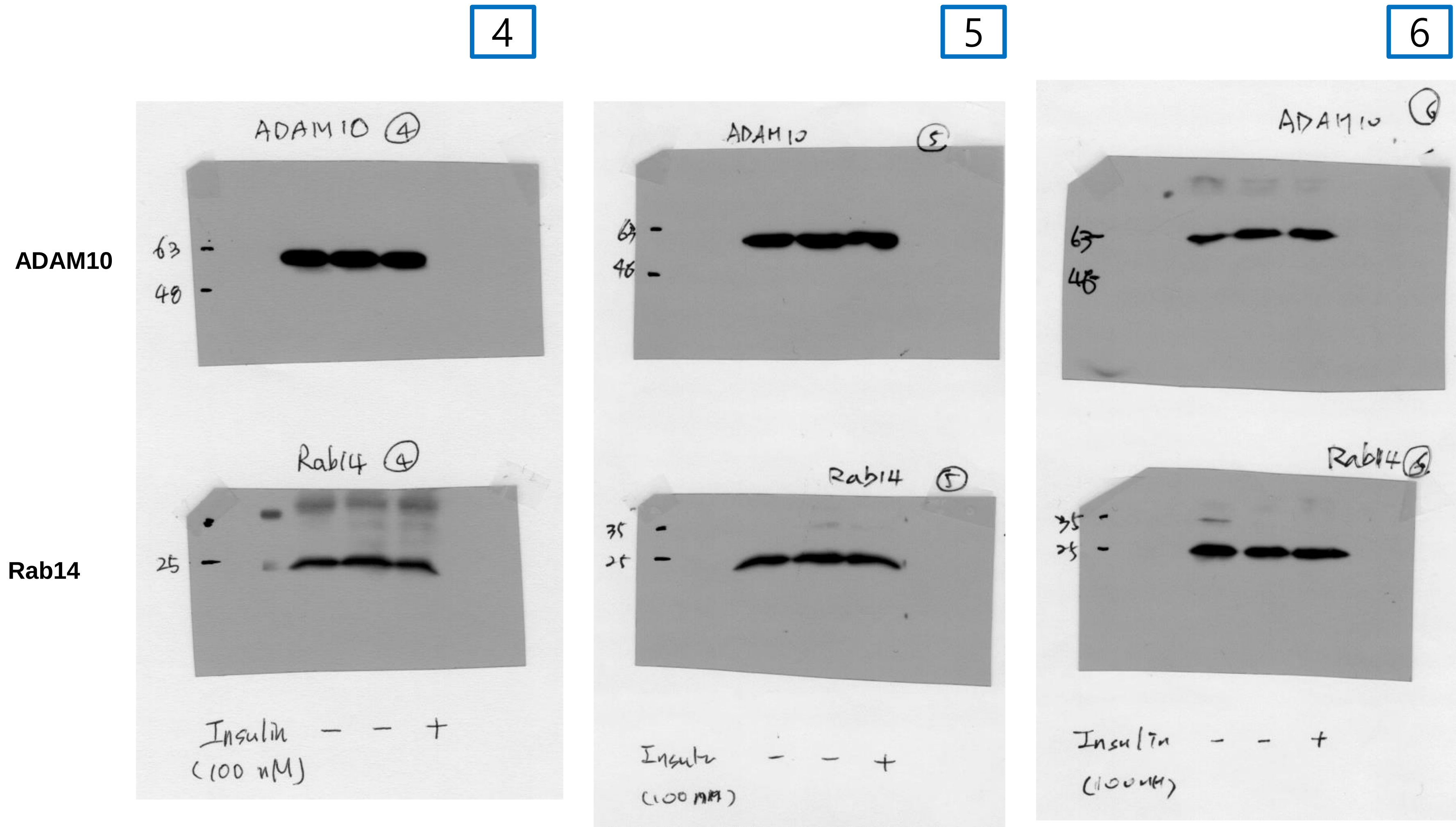

# Figure 9A: Rt panel - IP sample

ADAM10

Rab14

1

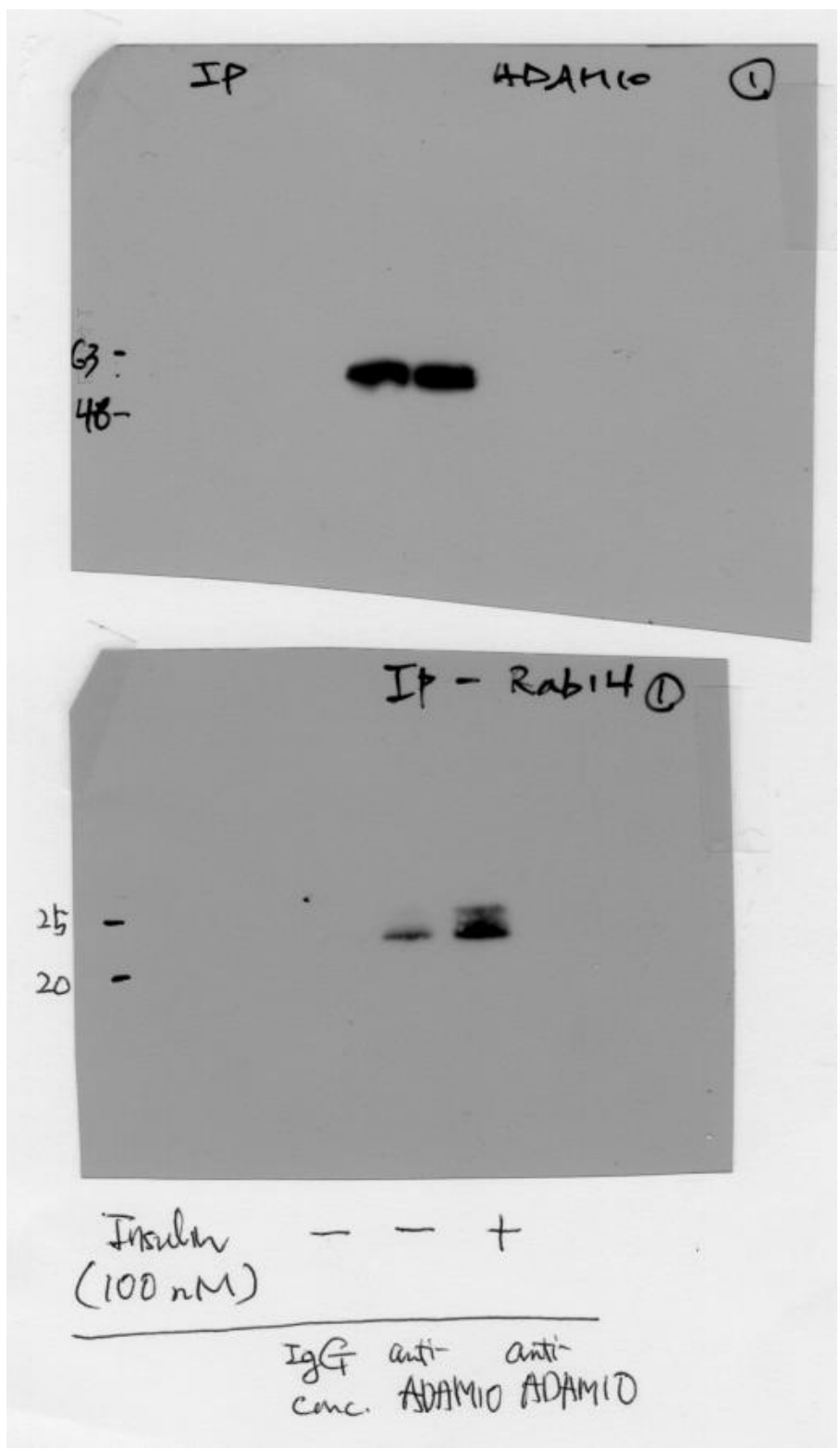


2

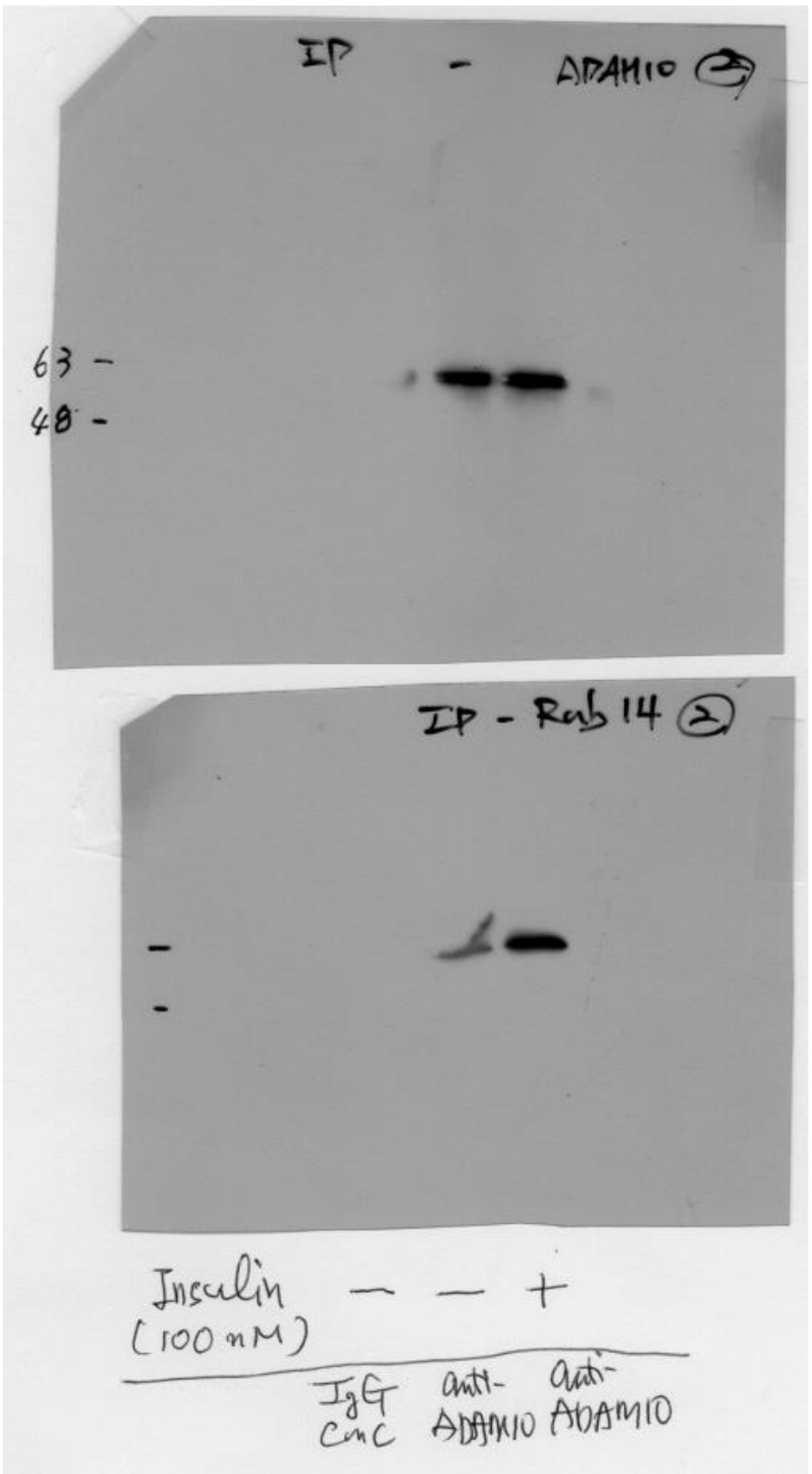


3

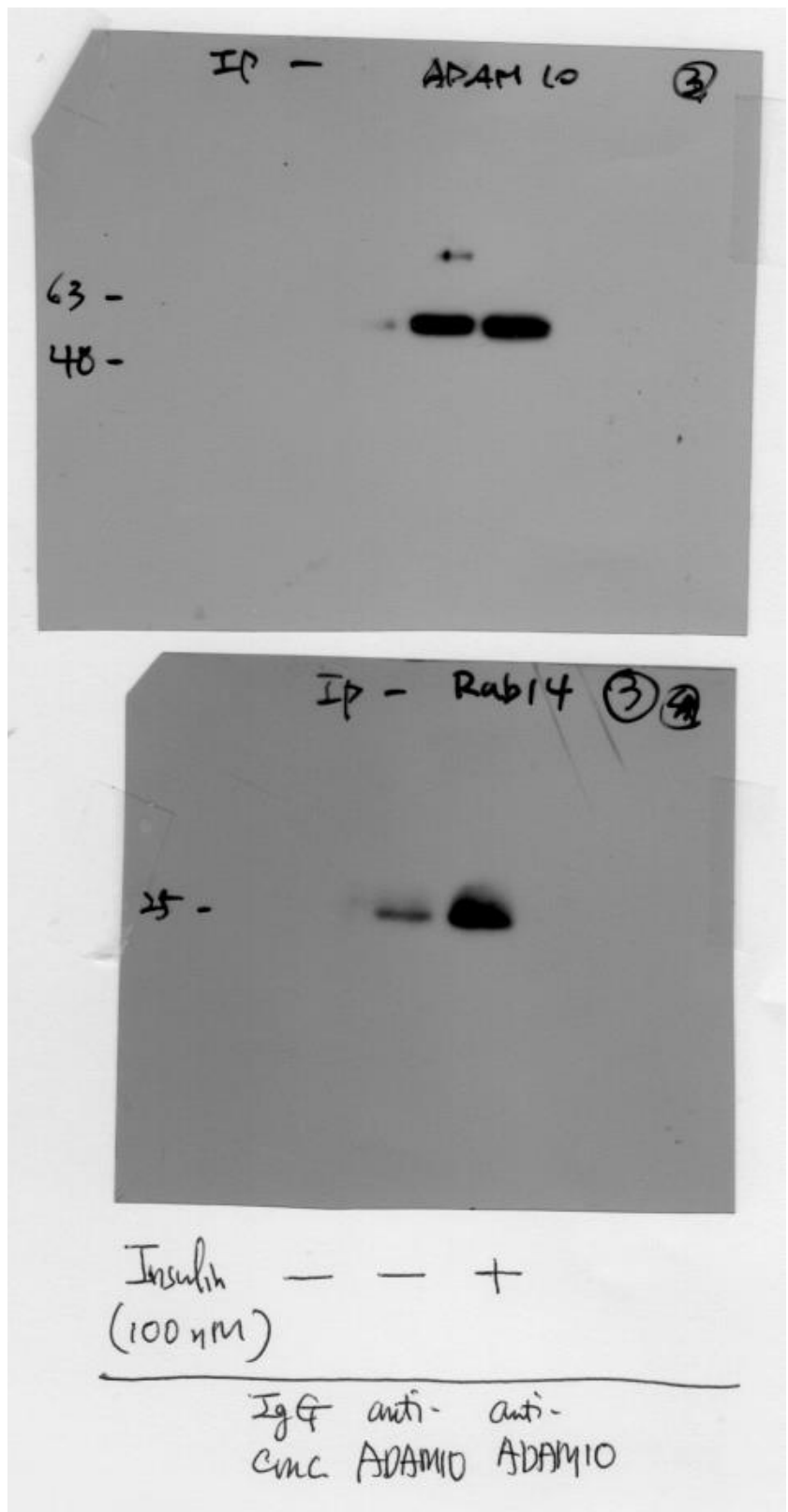

# Figure 9A: Rt panel - IP sample

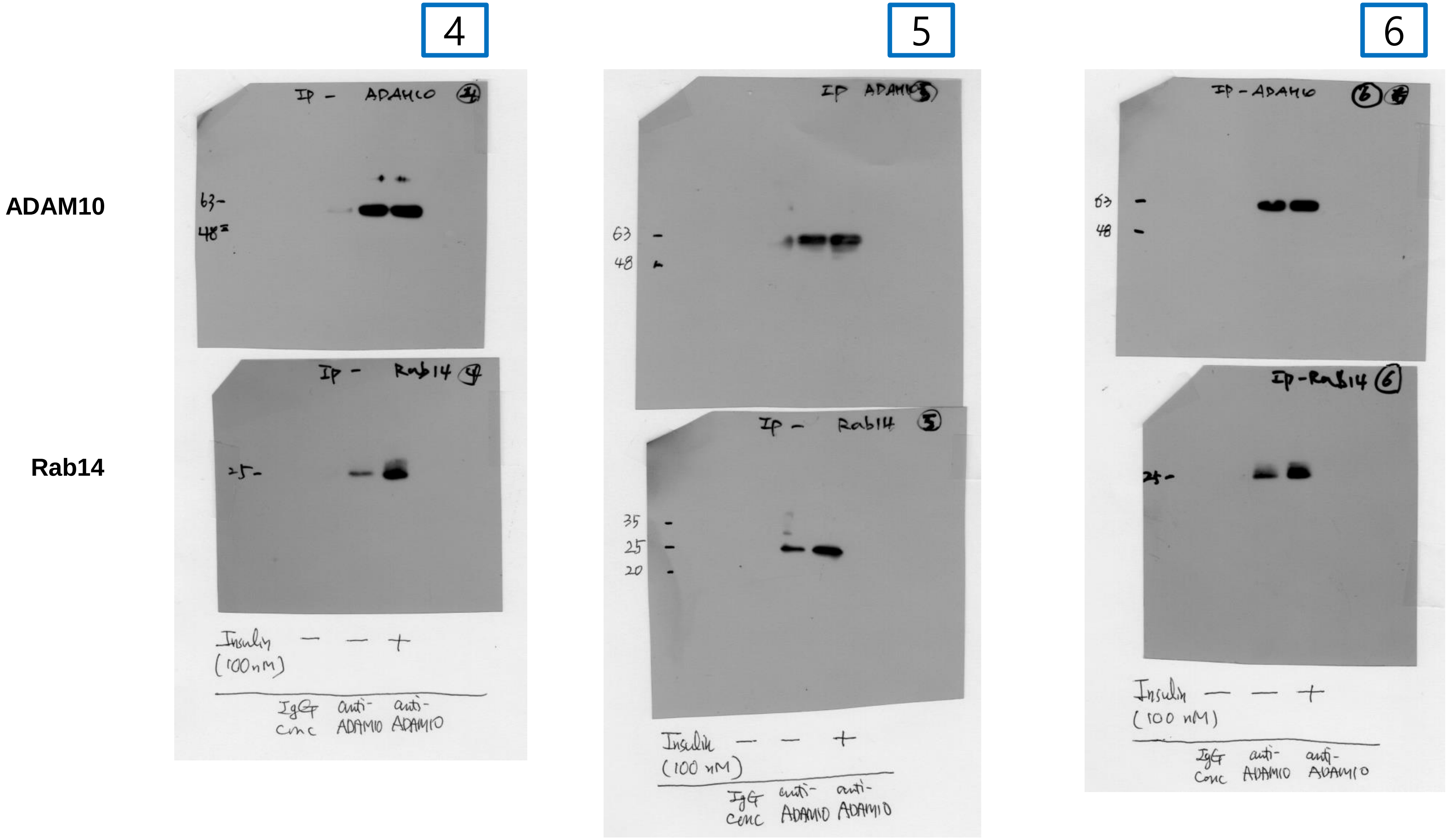

# Figure 9B - Whole cell lysates

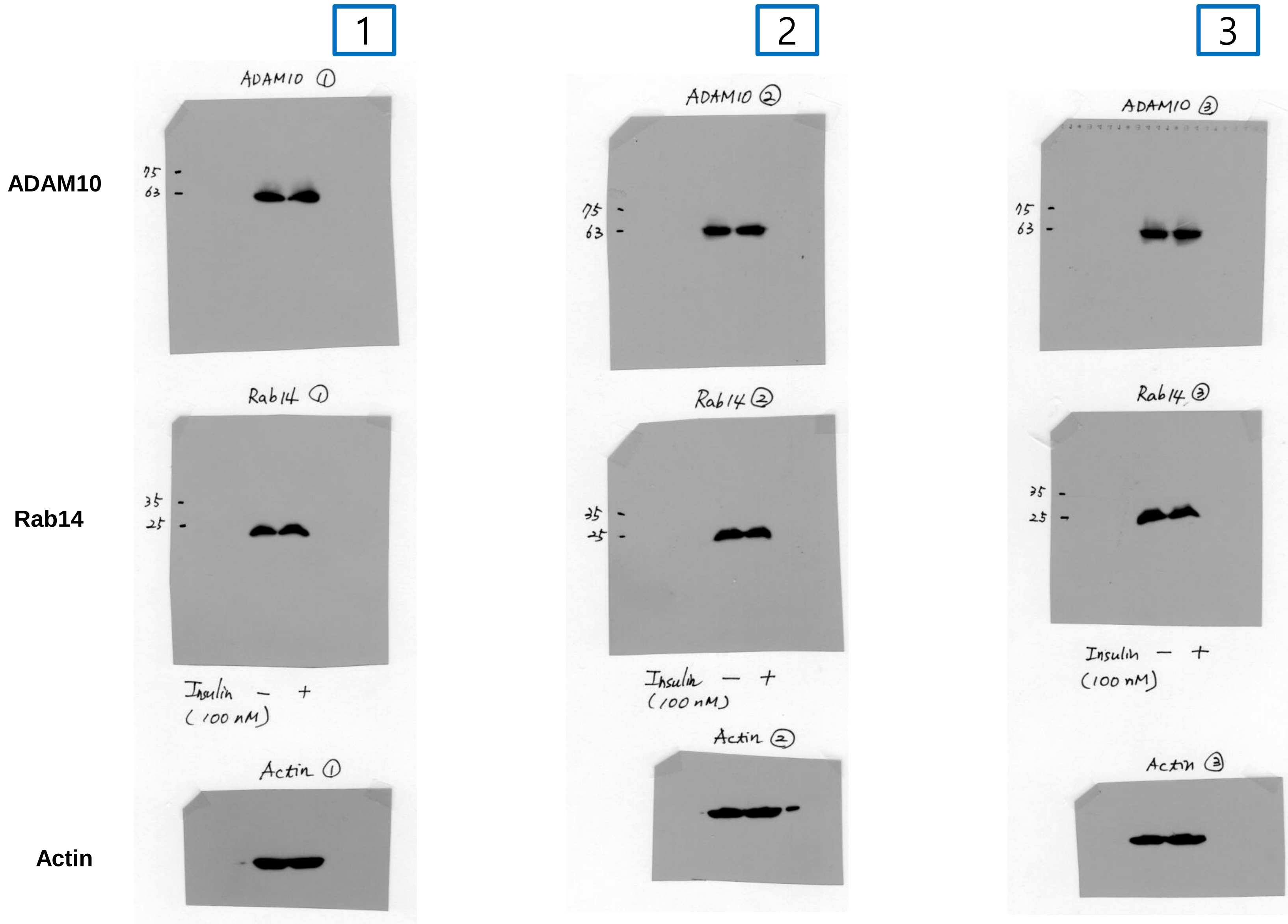

Figure 9B - Whole cell lysates

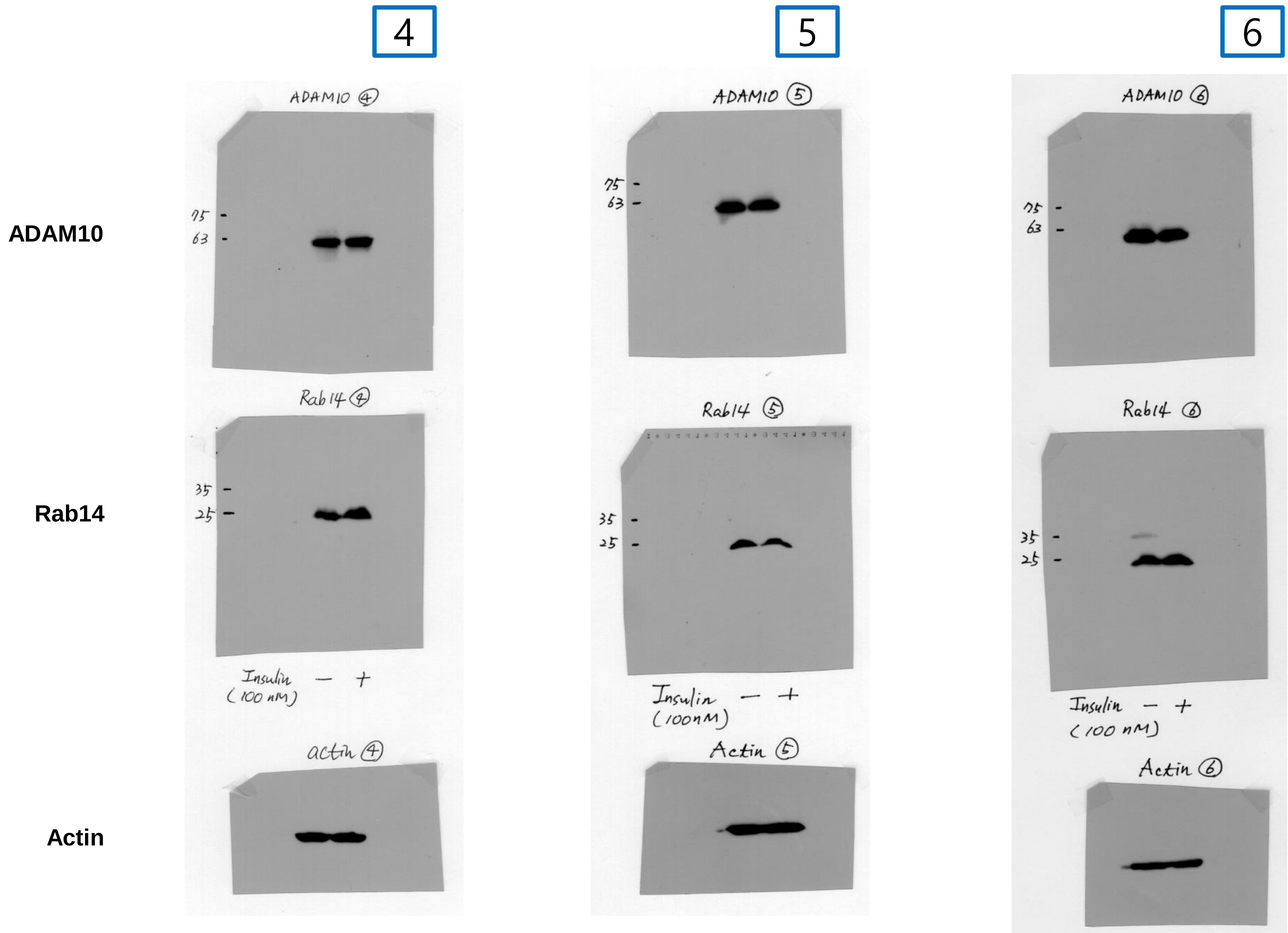

# Figure 9B - Intracellular proteins

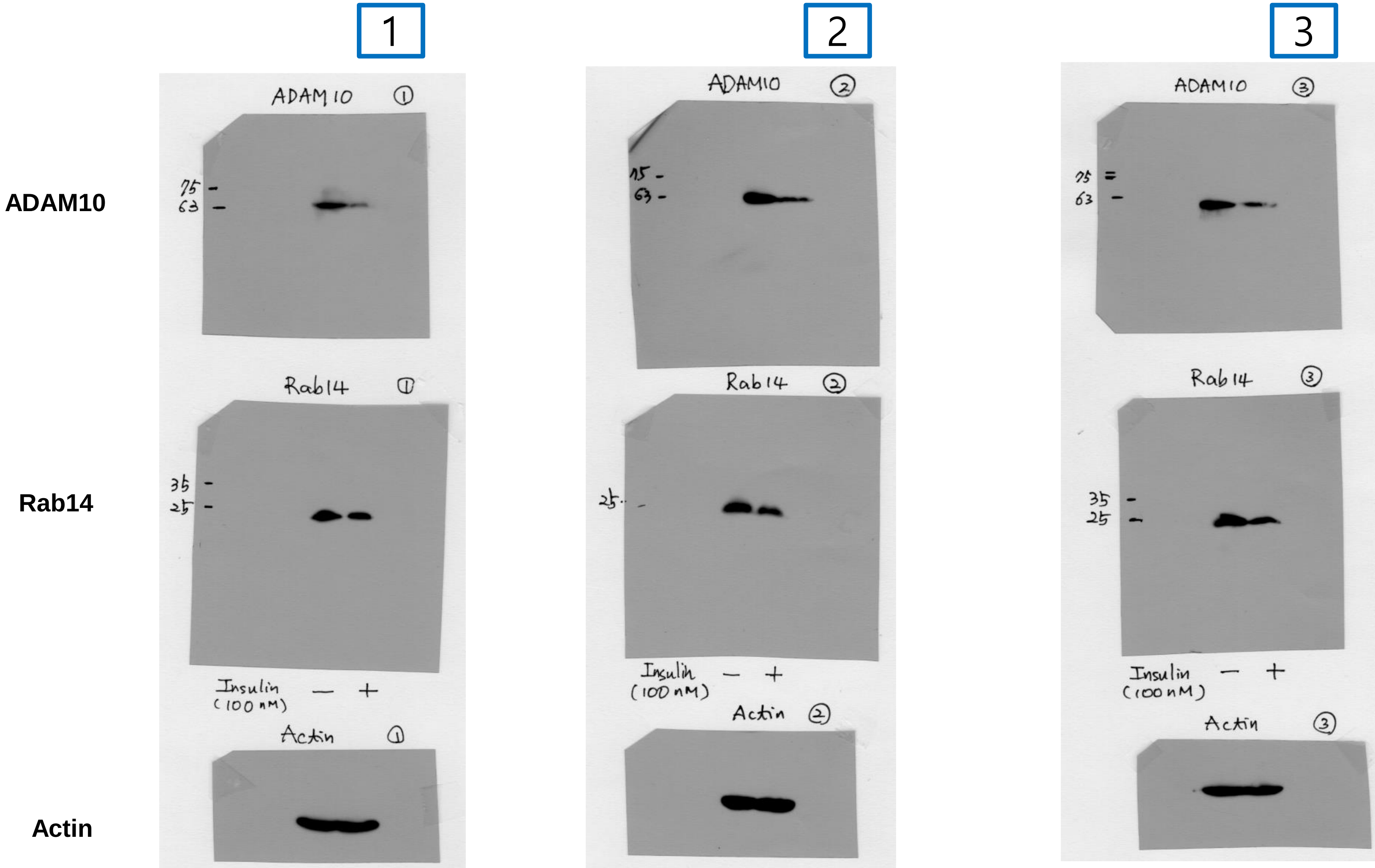

# Figure 9B - Intracellular proteins

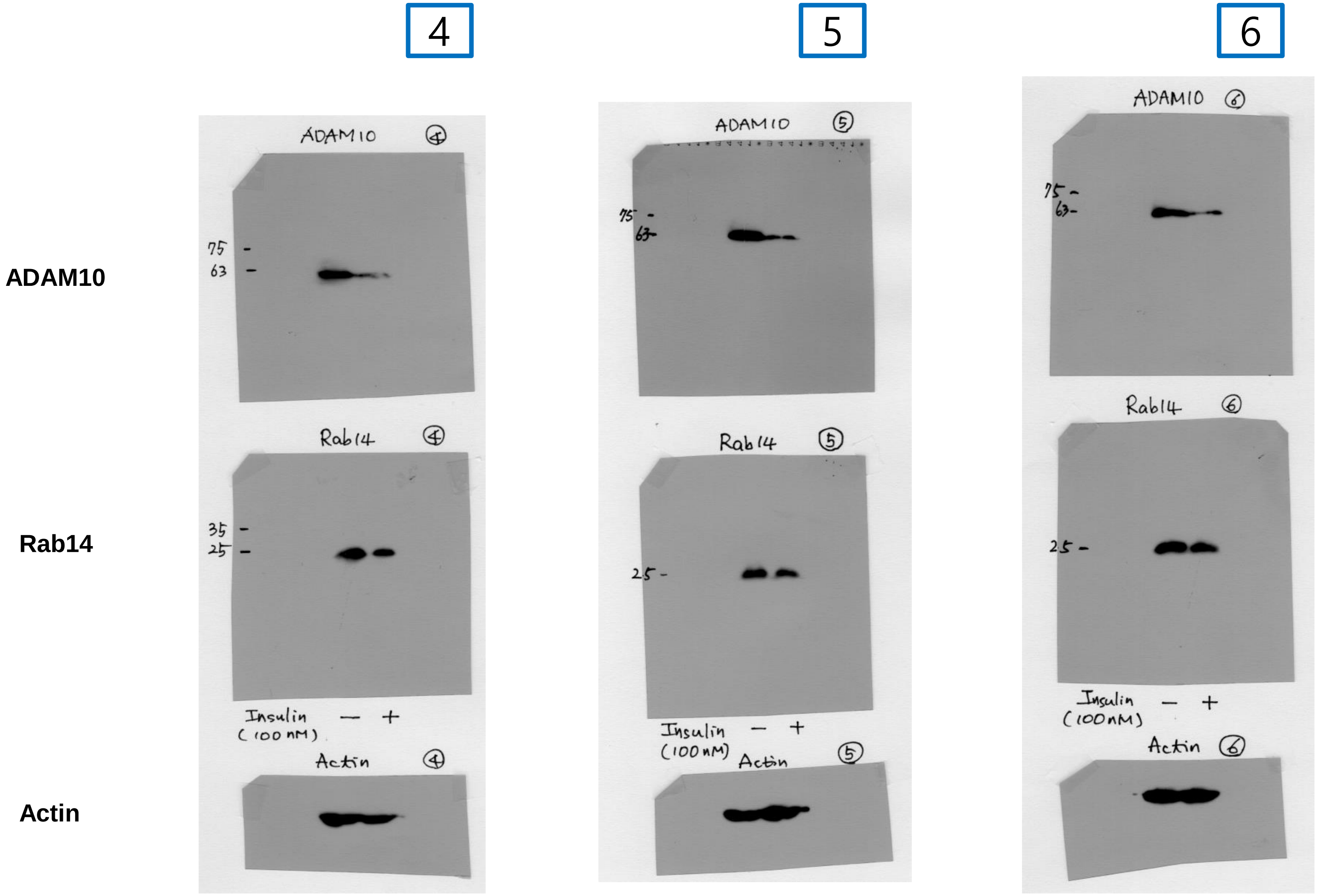

# Figure 9B - Cell surface proteins

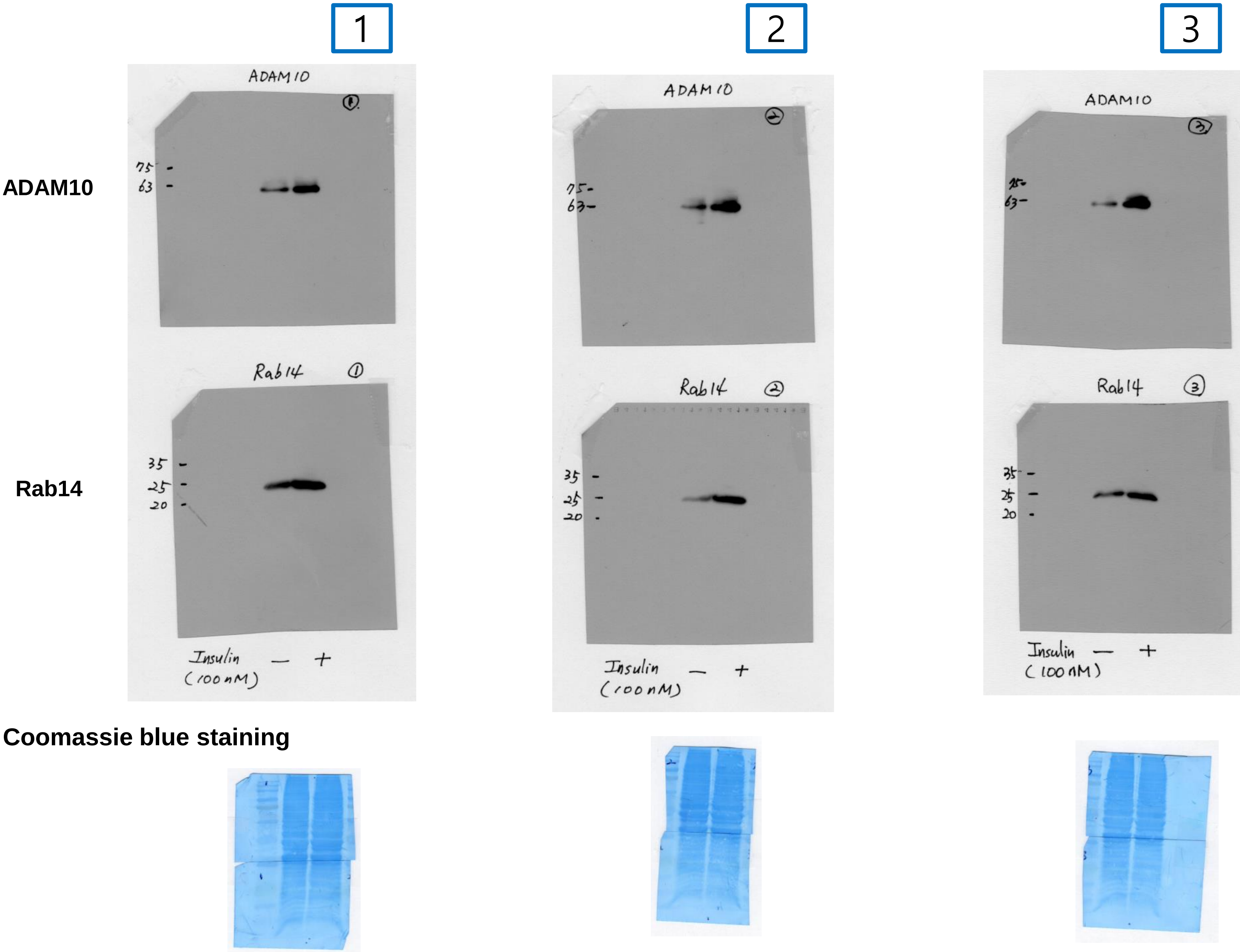

# Figure 9B - Cell surface proteins

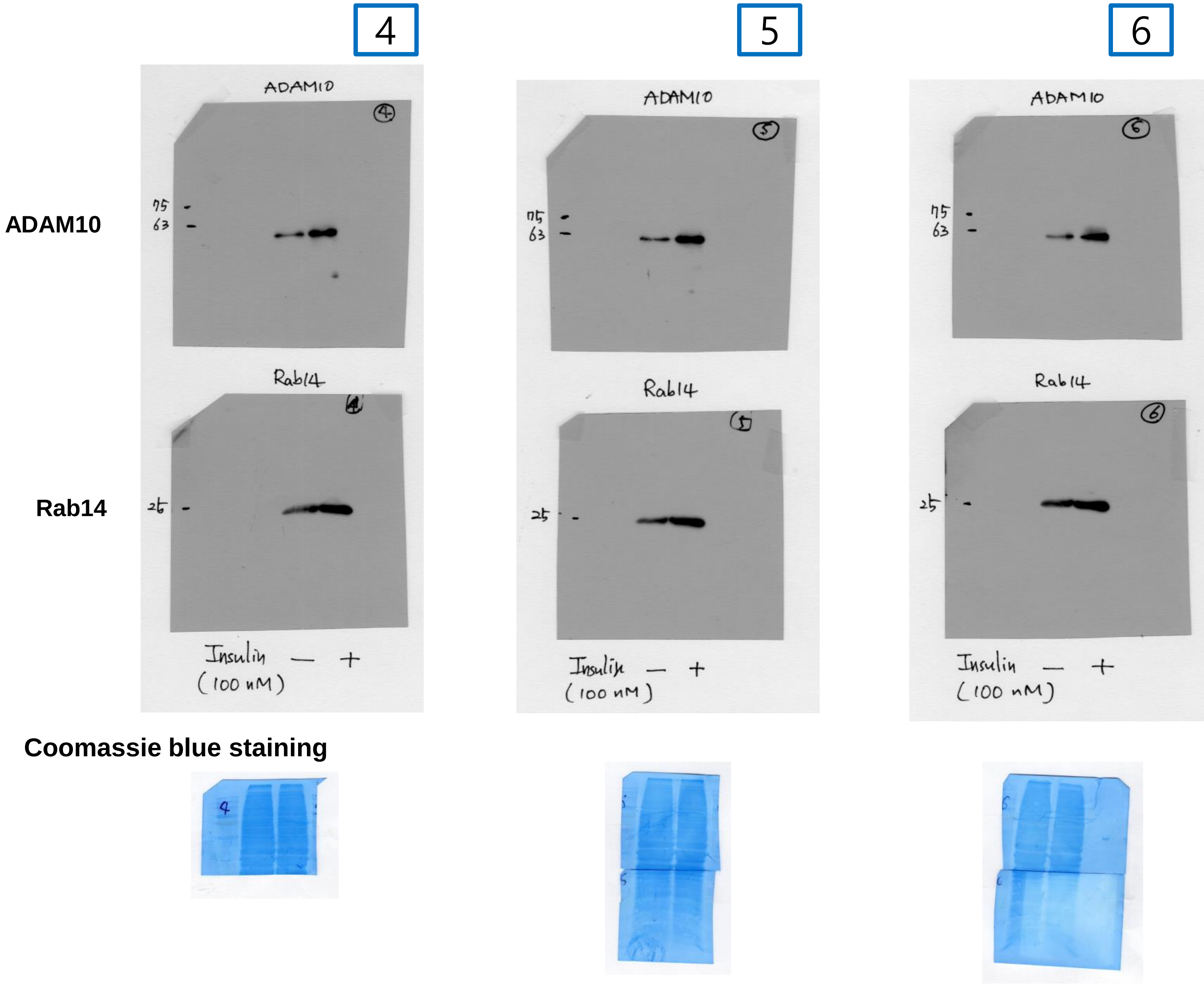

# Figure 10A (b)

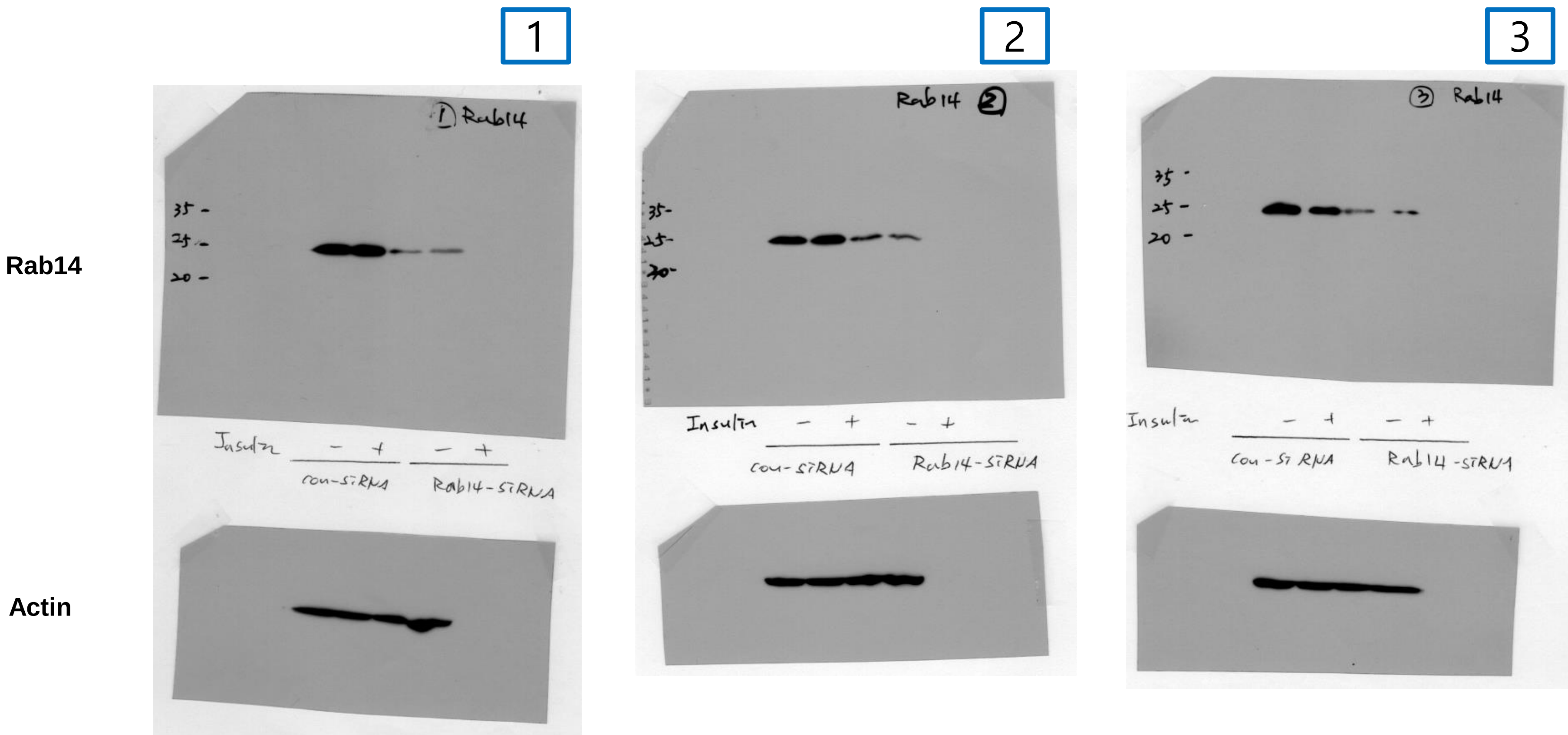

1
2
3
Rab14
Actin
① Rab14
35
25
20
Jasulin
− + − +
con-siRNA
Rab14-siRNA
Rab14 ②
35
25
30
Insulin
− + − +
con-siRNA
Rab14-siRNA
③ Rab14
35
25
20
Insulin
− + − +
Con-Si RNA
Rab14-siRNA

# Figure 10B upper panel

RAGE

1

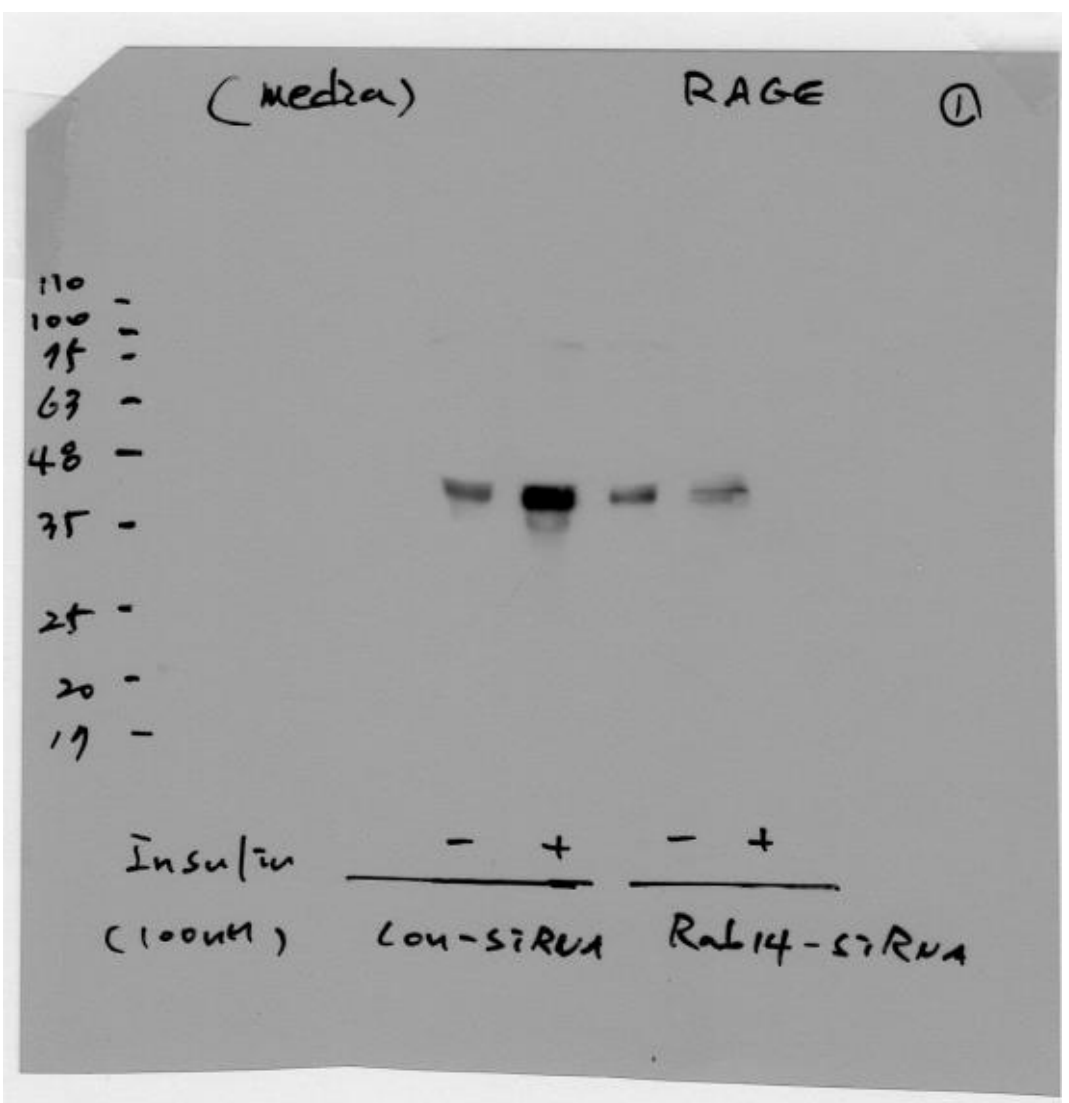


2

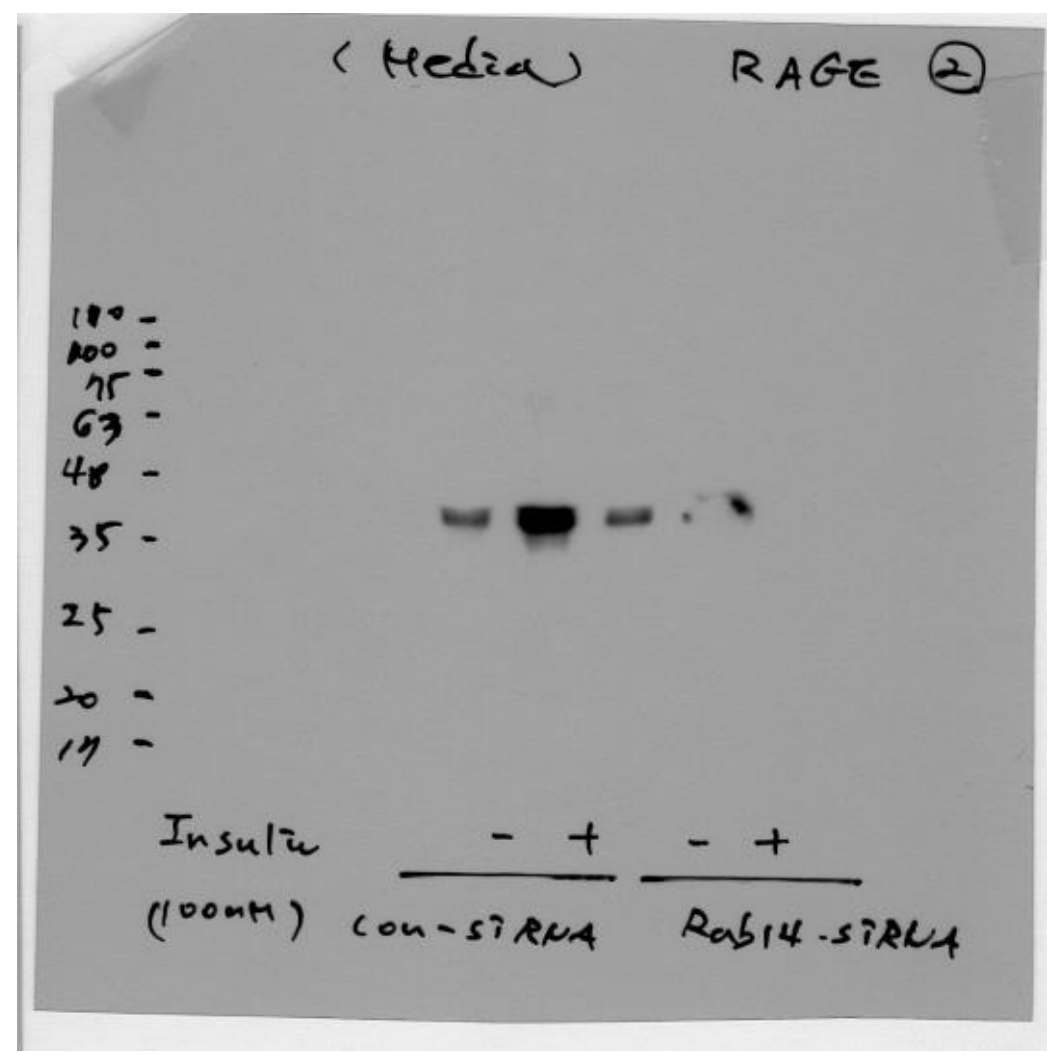


3

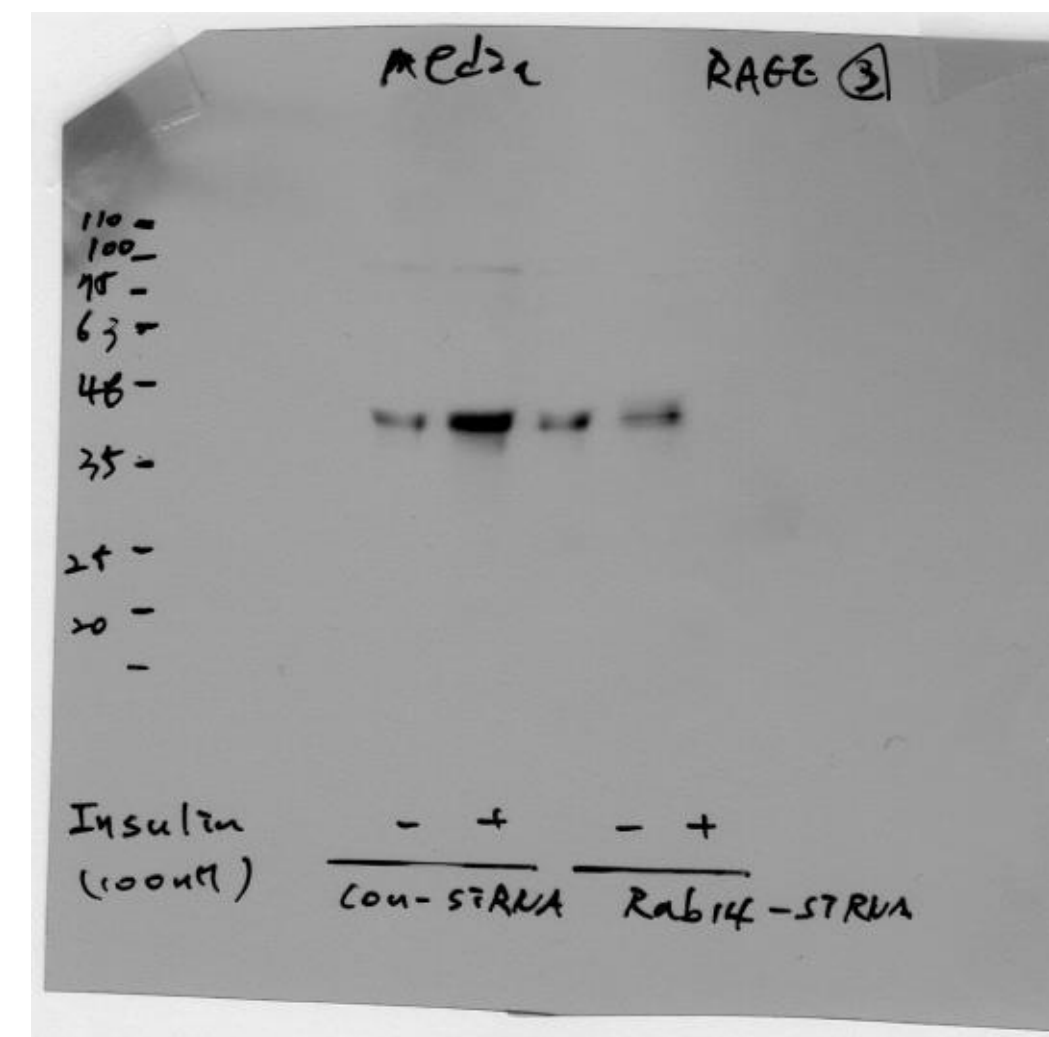

Figure 10B lower panel

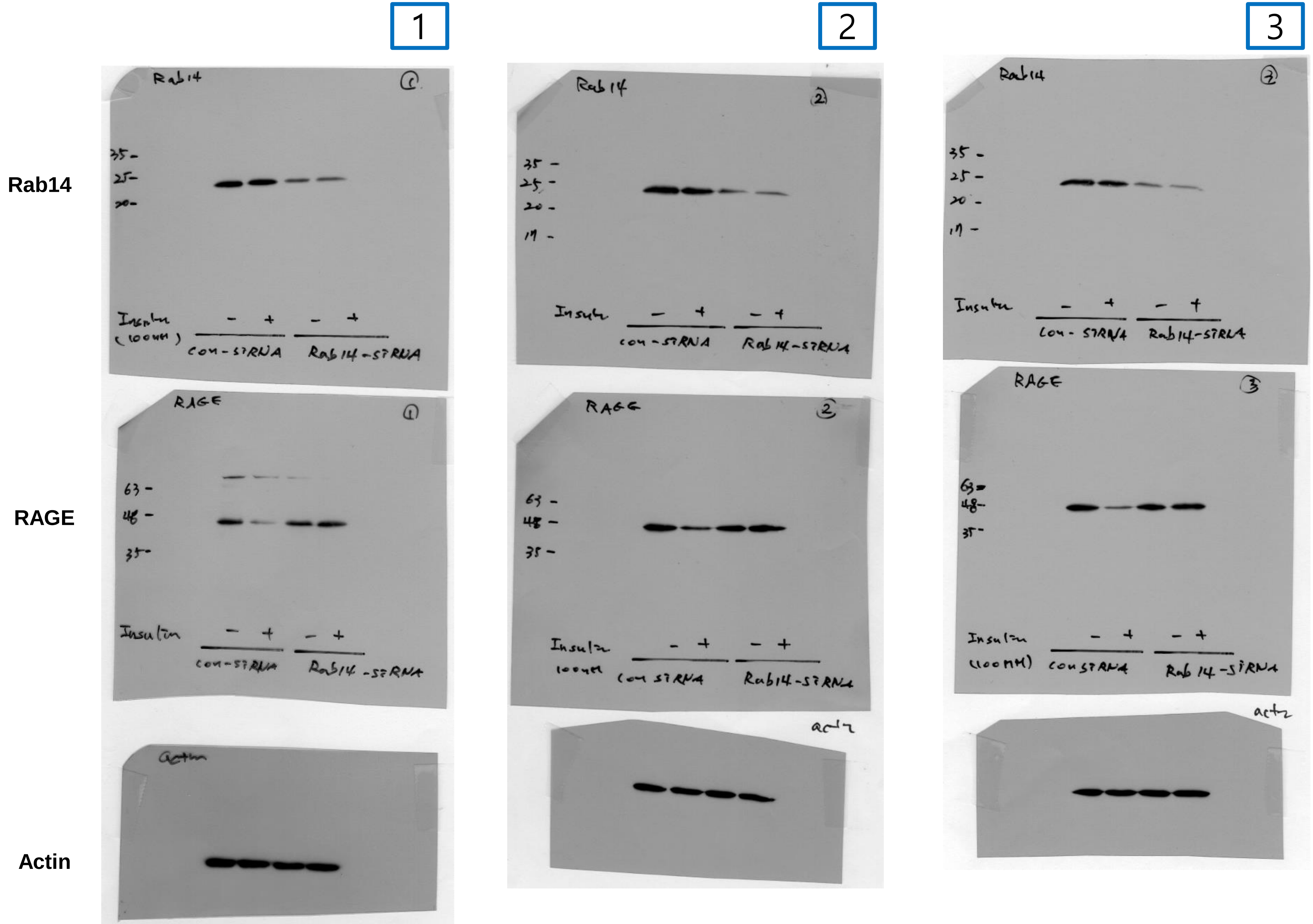

Figure 10C

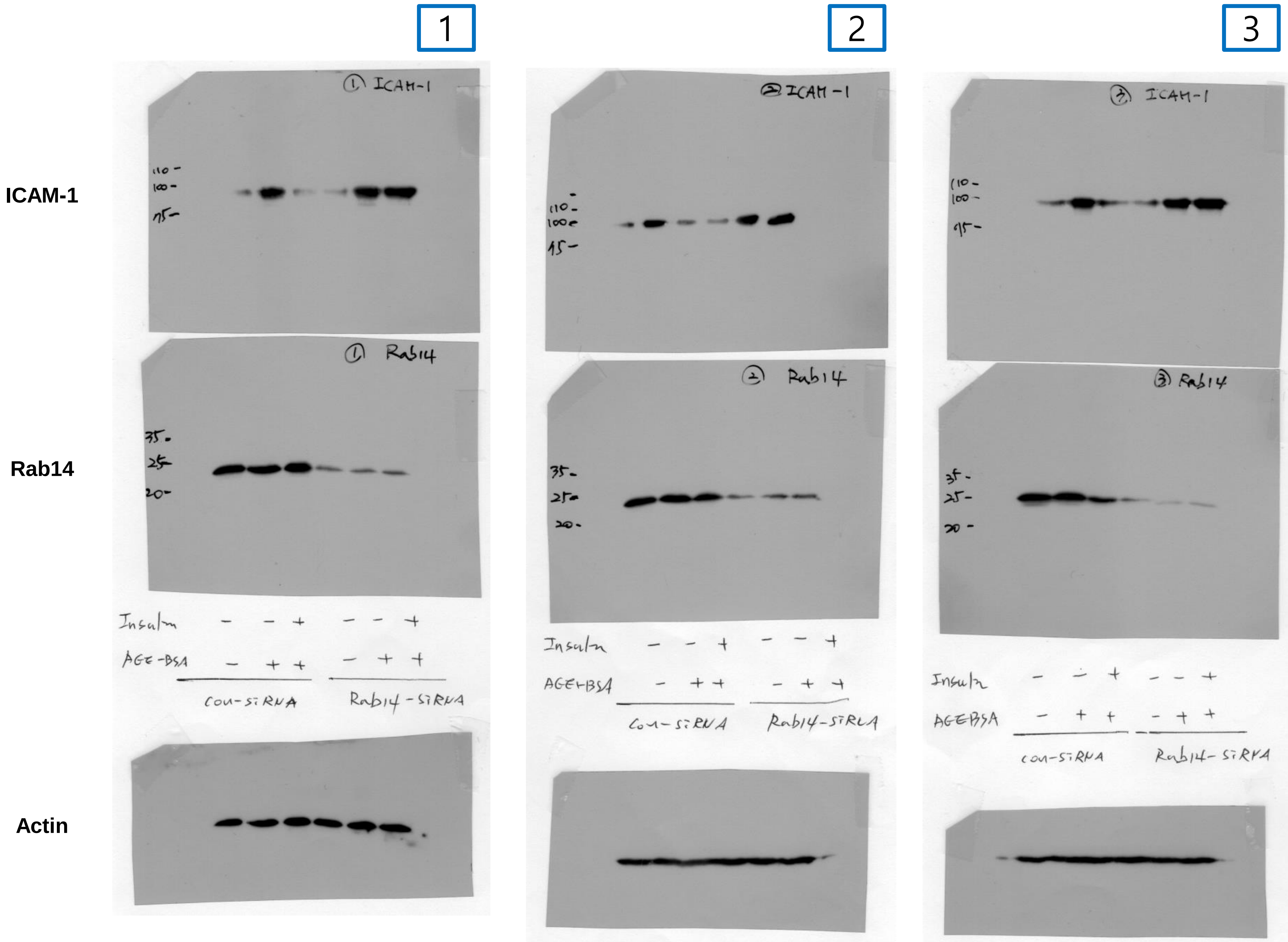

Figure 10C

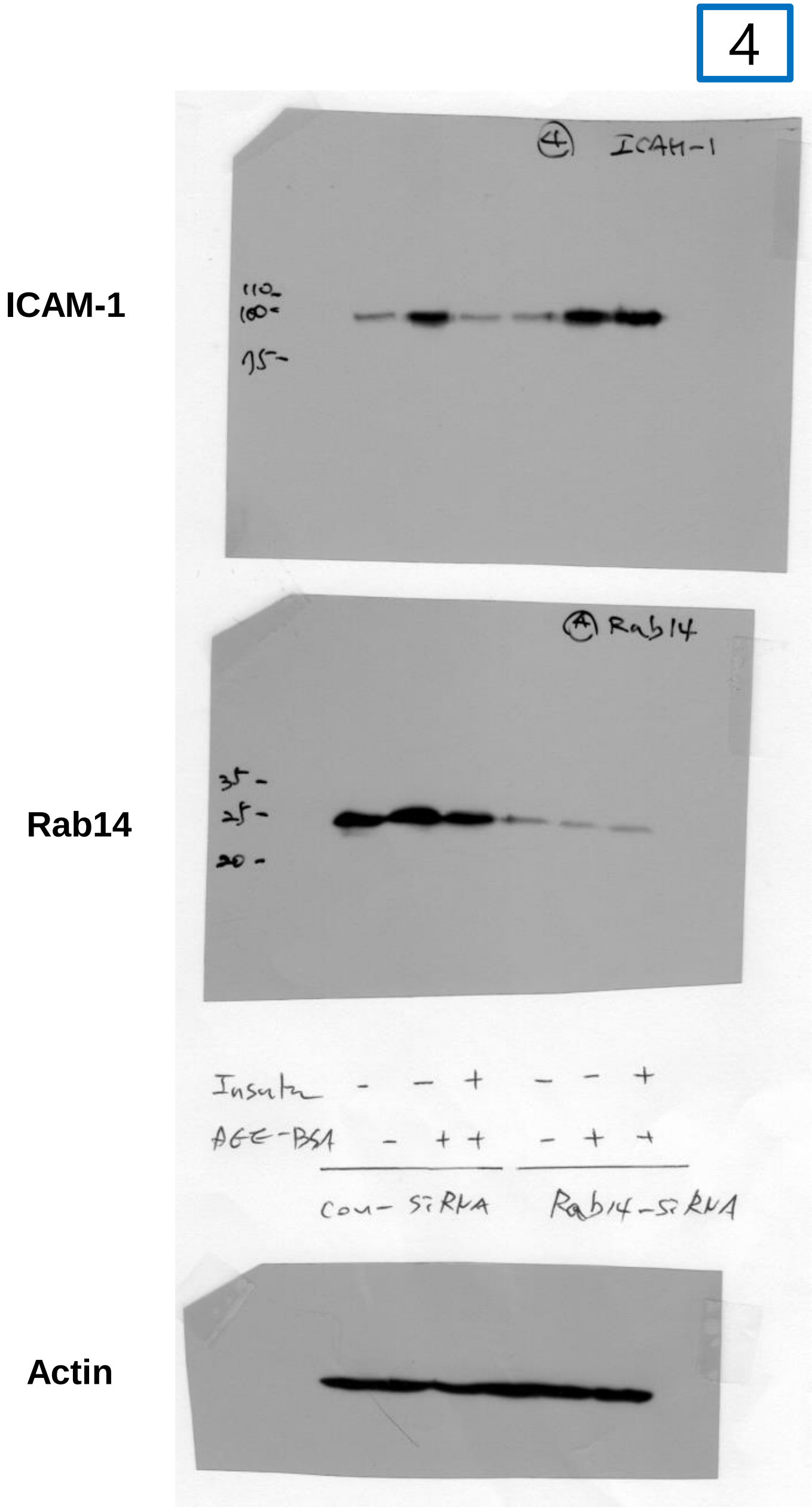
4
ICAM-1
Rab14
Actin
ICAM-1
Rab14
110
100
75
35
25
20
Insulin
AGE-BSA
