# Supplementary material for: Insulin enhances RAGE ectodomain shedding by inducing Rab14-dependent ADAM10 cell surface trafficking in human aortic endothelial cells
Source: PLoS One. 2026 Sep 18;21(9):e0358445. doi: 10.1371/journal.pone.0358445 (PMC13588500; doi:10.1371/journal.pone.0358445)

Insulin enhances RAGE ectodomain shedding by inducing Rab14-dependent ADAM10 cell surface trafficking in human aortic endothelial cells

Chung Hee Baek[a,1], Hyosang Kim[a,1], Soo Young Moon[b],
Eun Kyoung Lee[c], Won Seok Yang[a,*]

- IF staining Images -

Fig 7A (a)

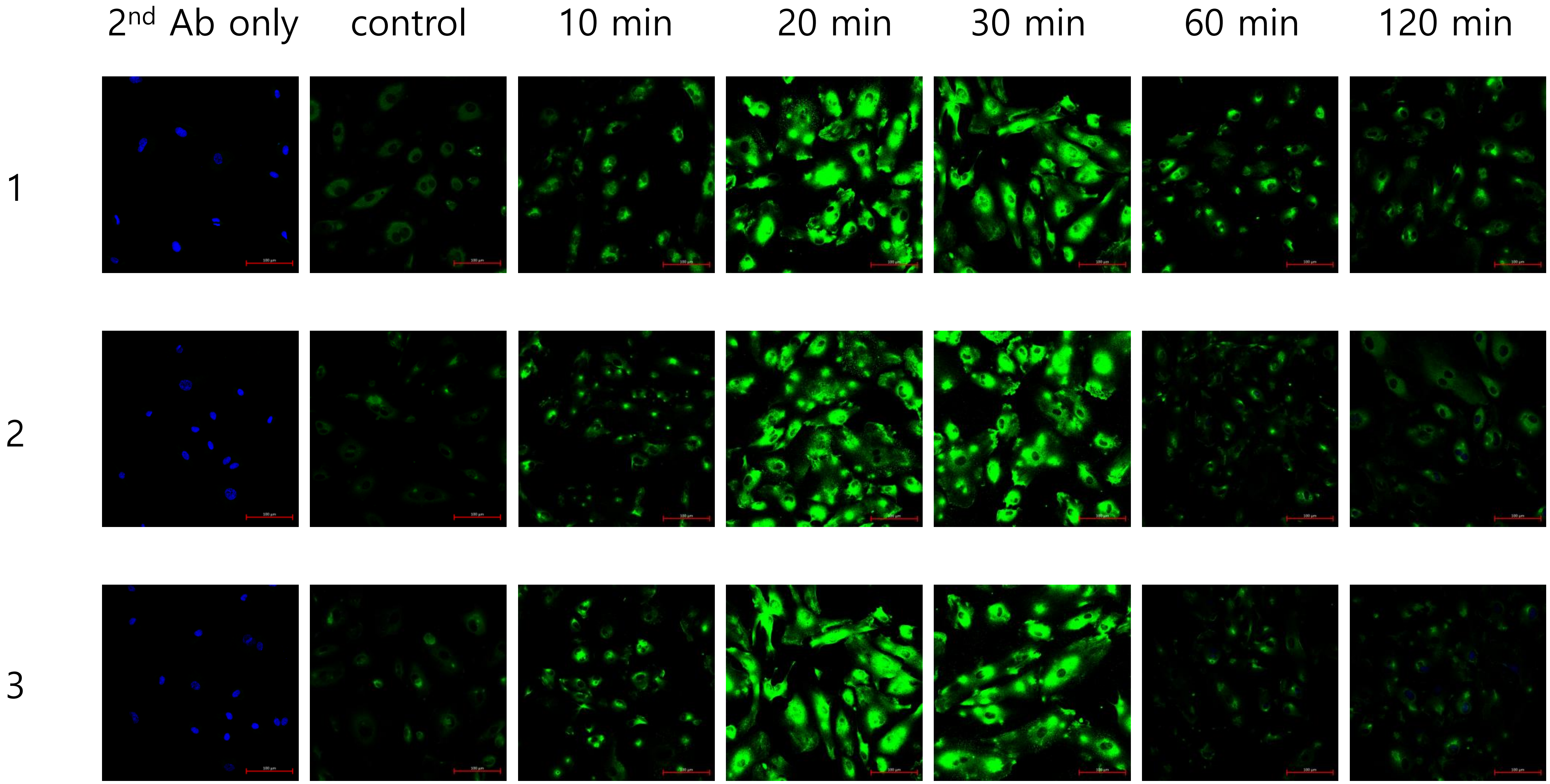

Fig 8A

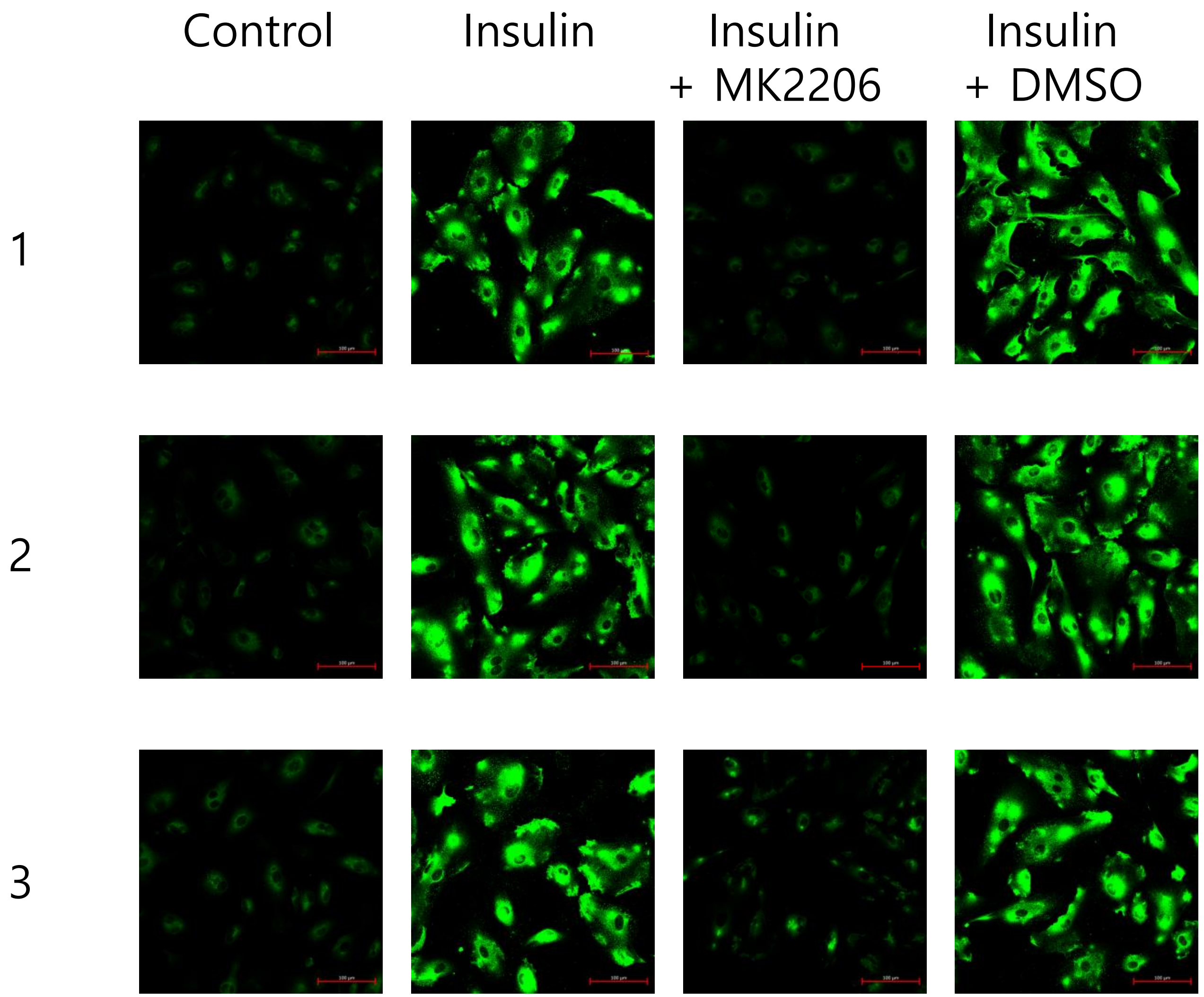

Fig 8B

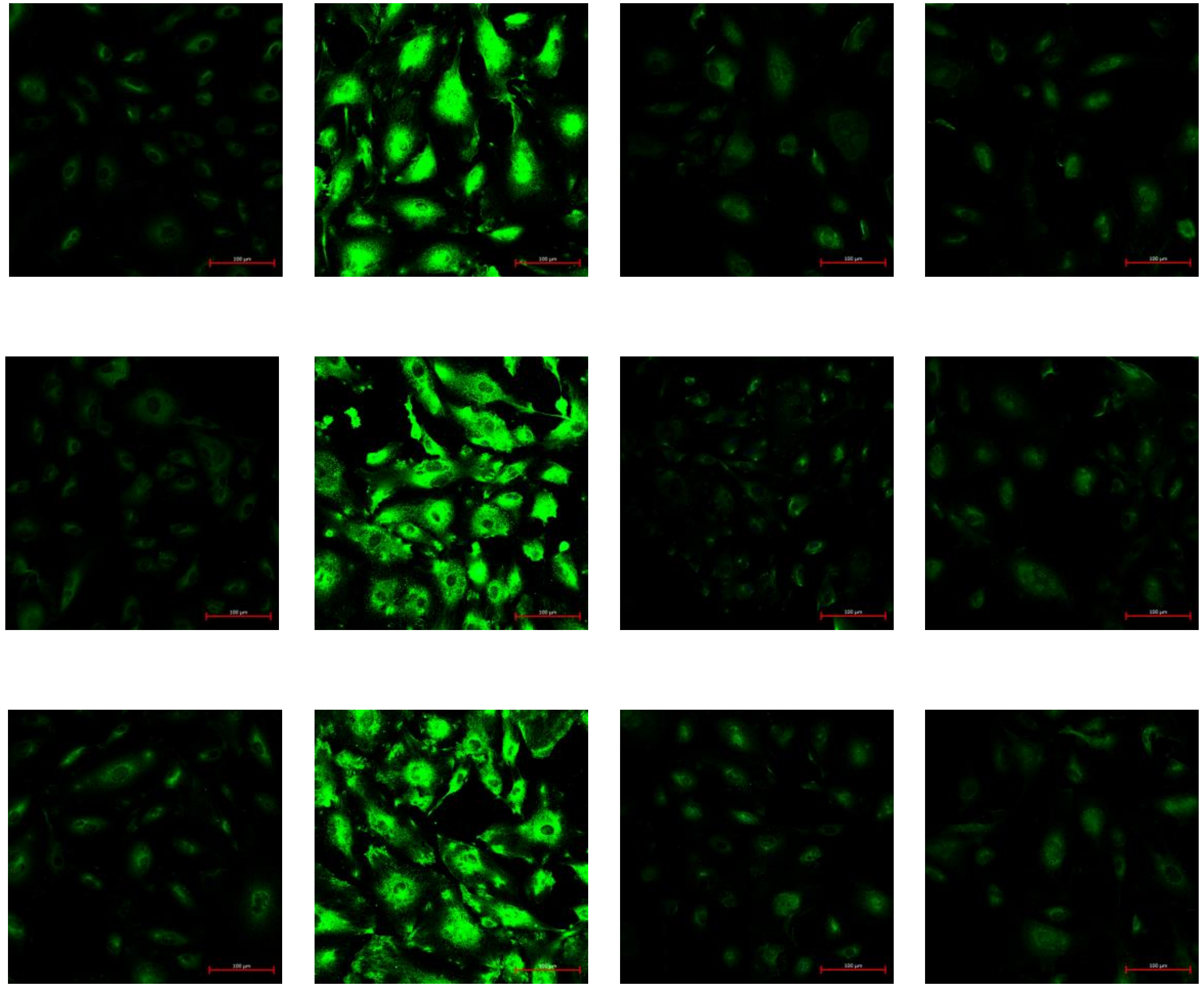

Fig 8C

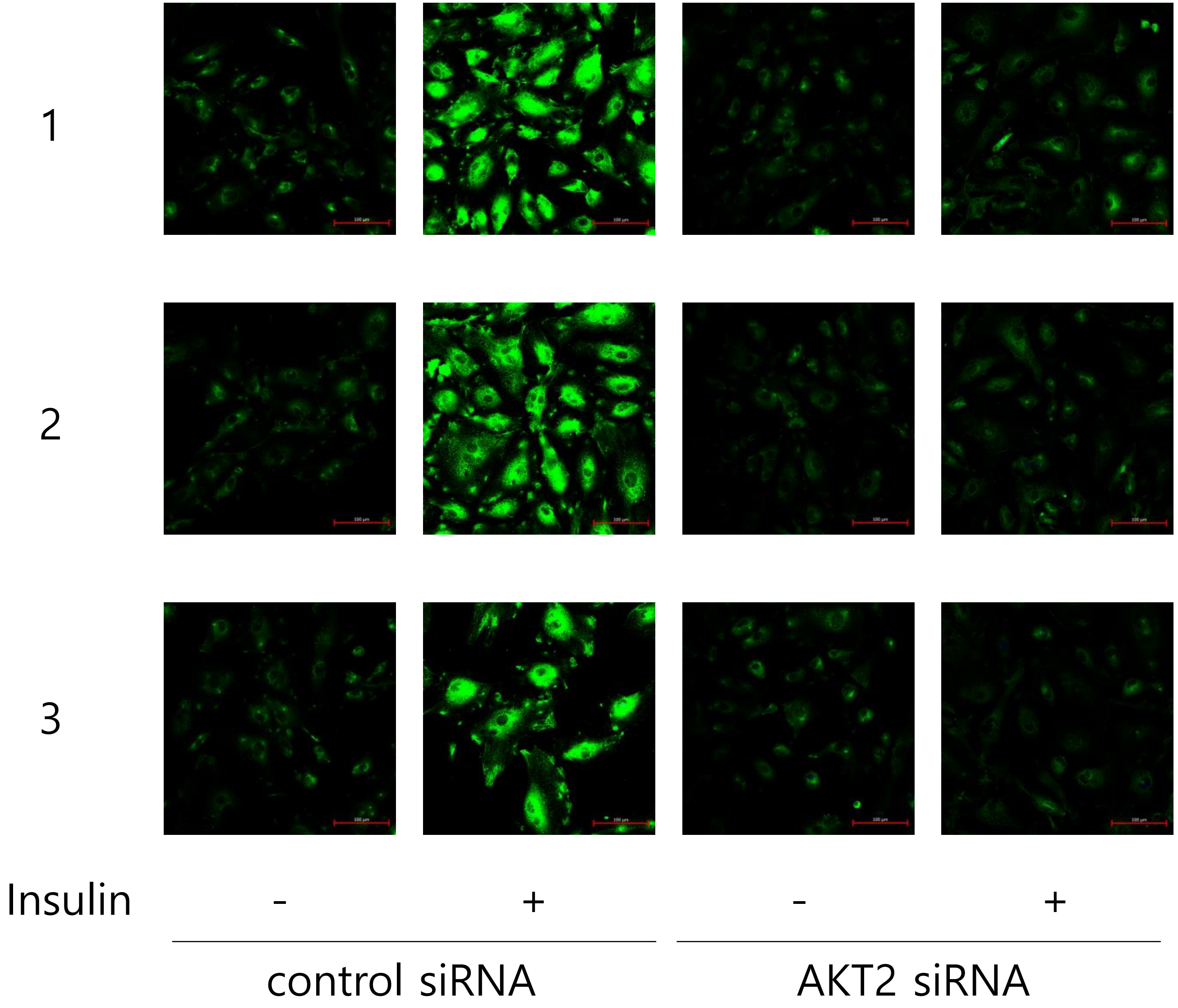

Fig 8D

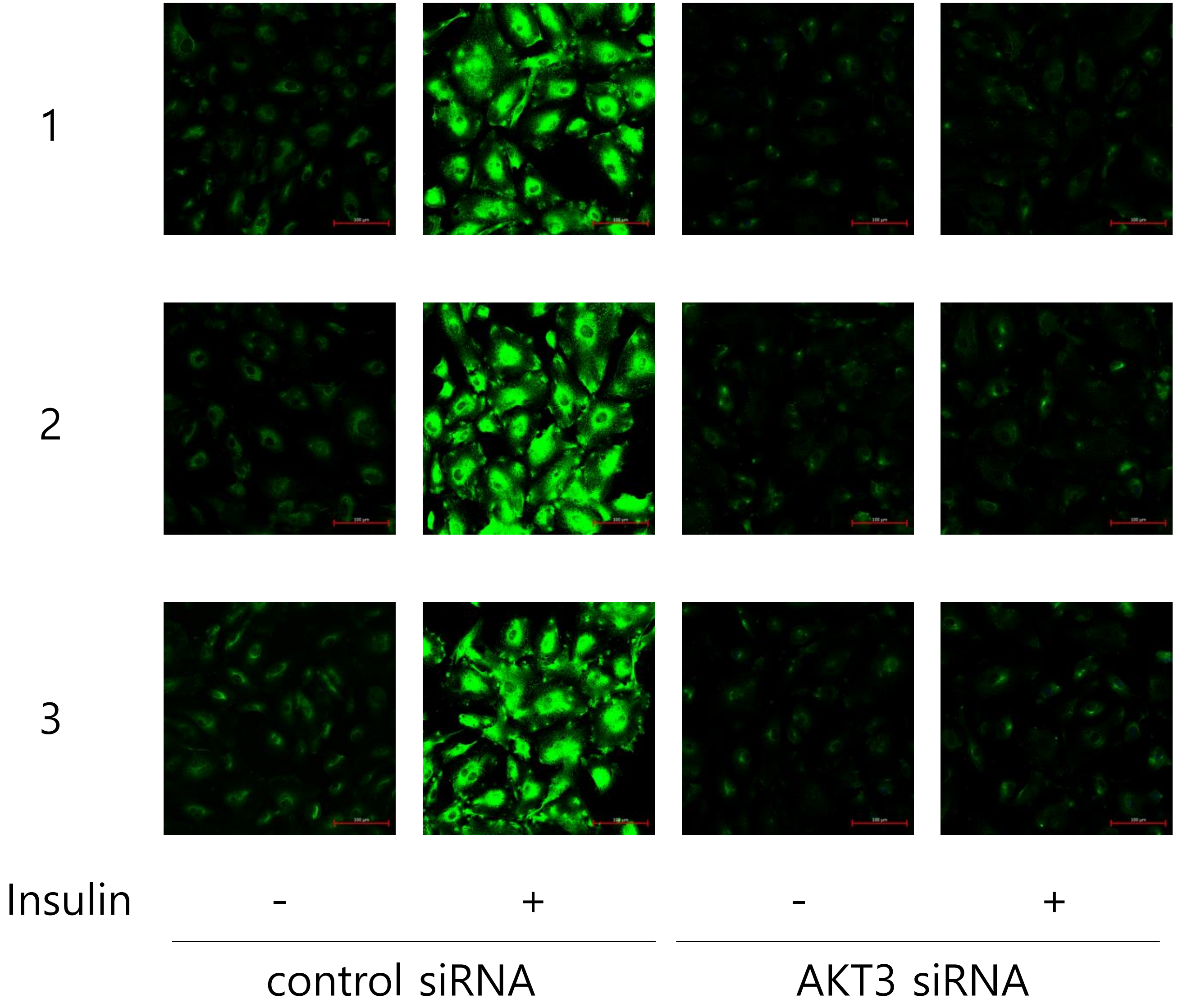

Fig 10A

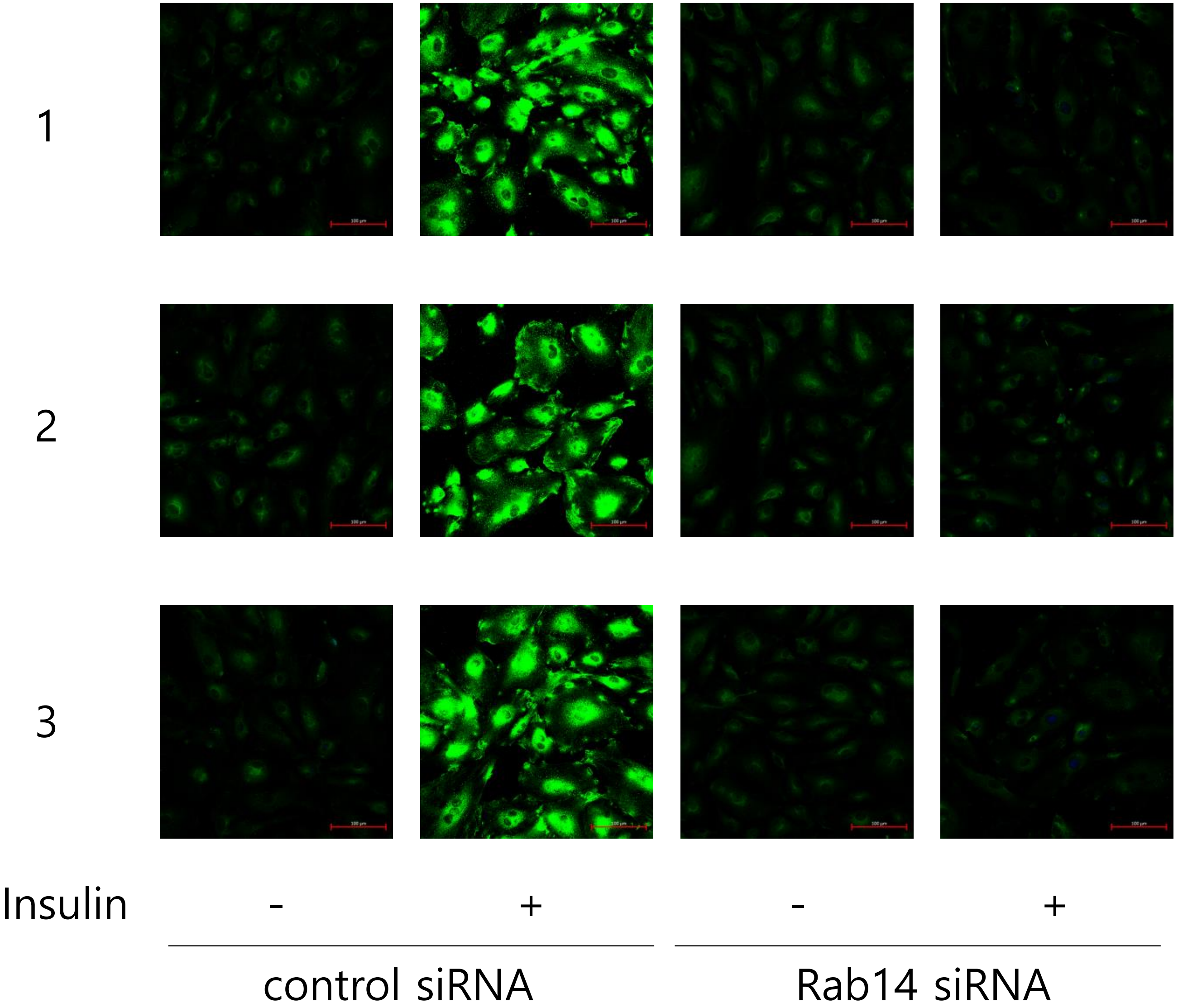

Supplement: Supplementary file 2 [file pone.0358445.s002.pdf]
